# Crystal structures of six miscellaneous products arising from the oxidation of precursors *R*^1^*R*^2^*R*^3^P*E*Au*X* (*R* = *tert*-butyl or isopropyl; *E* = S or Se; *X* = Cl, Br or I)

**DOI:** 10.1107/S2056989024010788

**Published:** 2024-11-28

**Authors:** Daniel Upmann, Dirk Bockfeld, Peter G. Jones

**Affiliations:** a Institut für Anorganische und Analytische Chemie, Technische Universität Braunschweig, Hagenring 30, D-38106 Braunschweig, Germany; Universität Greifswald, Germany

**Keywords:** crystal structure, gold, phosphane chalcogenides, secondary inter­actions

## Abstract

Compound **1**, (disulfane-1,2-di­yl)bis­(*tert*-butyl­diiso­propyl­phospho­nium) bis­[tetra­chlorido­aurate(III)], (^*t*^Bu^*i*^Pr_2_P)_2_S_2_·[AuCl_4_]_2_, contains the first structurally characterized dication of the form {(*R*_3_P)_2_*E*}_2_^2+^. The ions are linked by S⋯Cl contacts and C—H_methine_⋯Cl hydrogen bonds to form ribbons of residues parallel to the *a* axis. Compound **2** is bis­(di-*tert*-butyl­iso­propyl­phosphine sulfide-κ*S*)gold(I) triiodide/di­iodido­aurate(I)(0.905/0.095), [Au(C_11_H_25_PS)_2_][AuI_2_]_0.095_(I_3_)_0.905_, or [(^*t*^Bu_2_^*i*^PrPS)_2_Au]I_3_ with 9.5% of the triiodide replaced by di­iodido­aurate(I). Chains of alternating anions and cations parallel to [110] are formed by two S⋯I contacts. Compound **3** is bis­(*tert*-butyl­diiso­propyl­phosphine sulfide-κ*S*)gold(I) triiodide/di­iodido­aurate(I)(0.875/0.125), [Au(C_10_H_23_PS)_2_][AuI_2_]_0.125_(I_3_)_0.875_ or [(^*t*^Bu^*i*^Pr_2_PS)_2_Au]I_3_ with 12.5% of the triiodide replaced by di­iodo­aurate(I). Chains parallel to [101] are formed by two S⋯I contacts. Compound **4**, bis­(di-*tert*-butyl­iso­propyl­phosphine sulfide-κ*S*)gold(I) hepta­iodide, [Au(C_11_H_25_PS)_2_]I_7_ or [(^*t*^Bu_2_^*i*^PrPS)_2_Au]I_3_·2I_2_, is formally the bis-diiodine adduct of **3**, uncontaminated by di­iodido­aurate(I). The cations and anions display crystallographic twofold symmetry. The unbranched I_7_^−^ groups, I—I⋯I—I—I⋯I—I, are bent at the third and fifth atoms. The anions are linked by two S⋯I contacts to form a layer structure parallel to the *bc* plane. In all three structures **2**–**4**, there are also weak C—H_methine_⋯I contacts. Compound **5**, di­bromido­(di-*tert*-butyl­dithio­phosphato-κ^2^*S*,*S*′)gold(III), [AuBr_2_(C_8_H_18_PS_2_)] or [AuBr_2_(^*t*^Bu_2_PS_2_)], contains a four-membered chelate ring. It crystallizes with imposed mirror symmetry. An S⋯Br contact links the mol­ecules to form corrugated layers parallel to the *bc* plane. In compound **6**, di-*tert*-but­yl{[di-*tert*-but­yl(hy­droxy)phosphan­yl]diselan­yl}phosphine oxide tetra­bromido­aurate(III), (C_16_H_37_O_2_P_2_Se_2_)[AuBr_4_] or [(^*t*^Bu_2_OPSe)_2_H][AuBr_4_], the cation has a central diselenide unit, and also displays an intra­cationic hydrogen bond O—H⋯O. Two Se⋯Br contacts link the residues to form zigzag chains parallel to [201]. The problem of determining whether an *E*⋯*X* contact (*E* = chalcogen, *X* = halogen) represents a halogen bond or a chalcogen bond is discussed.

## Abstract 2

Various compounds involving phosphane chalcogenides (or their derivatives) and gold have been characterized; their packing is analysed in terms of weak hydrogen bonds and halogen⋯chalcogen contacts.

## 1. Chemical context

In parts 6–8 of this series, we presented the structures of gold(I) complexes [(*R*^1^*R*^2^*R*^3^P*E*)Au*X*] (Upmann *et al.*, 2024*a* ▸), gold(III) complexes [(*R*^1^*R*^2^*R*^3^P*E*)Au*X*_3_] (Upmann *et al.*, 2024*b* ▸) and the further oxidized phospho­nium gold(III) deriv­atives (*R*^1^*R*^2^*R*^3^P*EX*)^+^ [Au*X*_4_]^−^ (Upmann *et al.*, 2024*c* ▸), where the *R* groups are *tert*-butyl or isopropyl, the chalcogens *E* are S or Se, and the halogens *X* are Cl or Br. The iodido-Au^I^ derivatives are not oxidizable in this way. The two steps [(*R*^1^*R*^2^*R*^3^P*E*)Au*X*] → [(*R*^1^*R*^2^*R*^3^P*E*)Au*X*_3_] → (*R*^1^*R*^2^*R*^3^P*EX*)^+^[Au*X*_4_]^−^ each correspond to the addition of two halogen atoms per gold atom. Mixed-valence compounds of the form [(*R*^1^*R*^2^*R*^3^P*E*)_2_Au]^+^[Au*X*_4_]^−^ were also isolated (Part 9; Up­mann *et al.*, 2024*d* ▸), corresponding to the addition of one halogen atom per gold atom of the Au^I^ precursors. Some syntheses, however, failed completely, some led to ‘wrong’ products or decomposition products and some formed mixtures that were difficult to separate. Several of these miscellaneous products, some obtained only in low yields and/or as mixtures, were however characterized by X-ray structure analysis, and the structures of six of these are presented here.
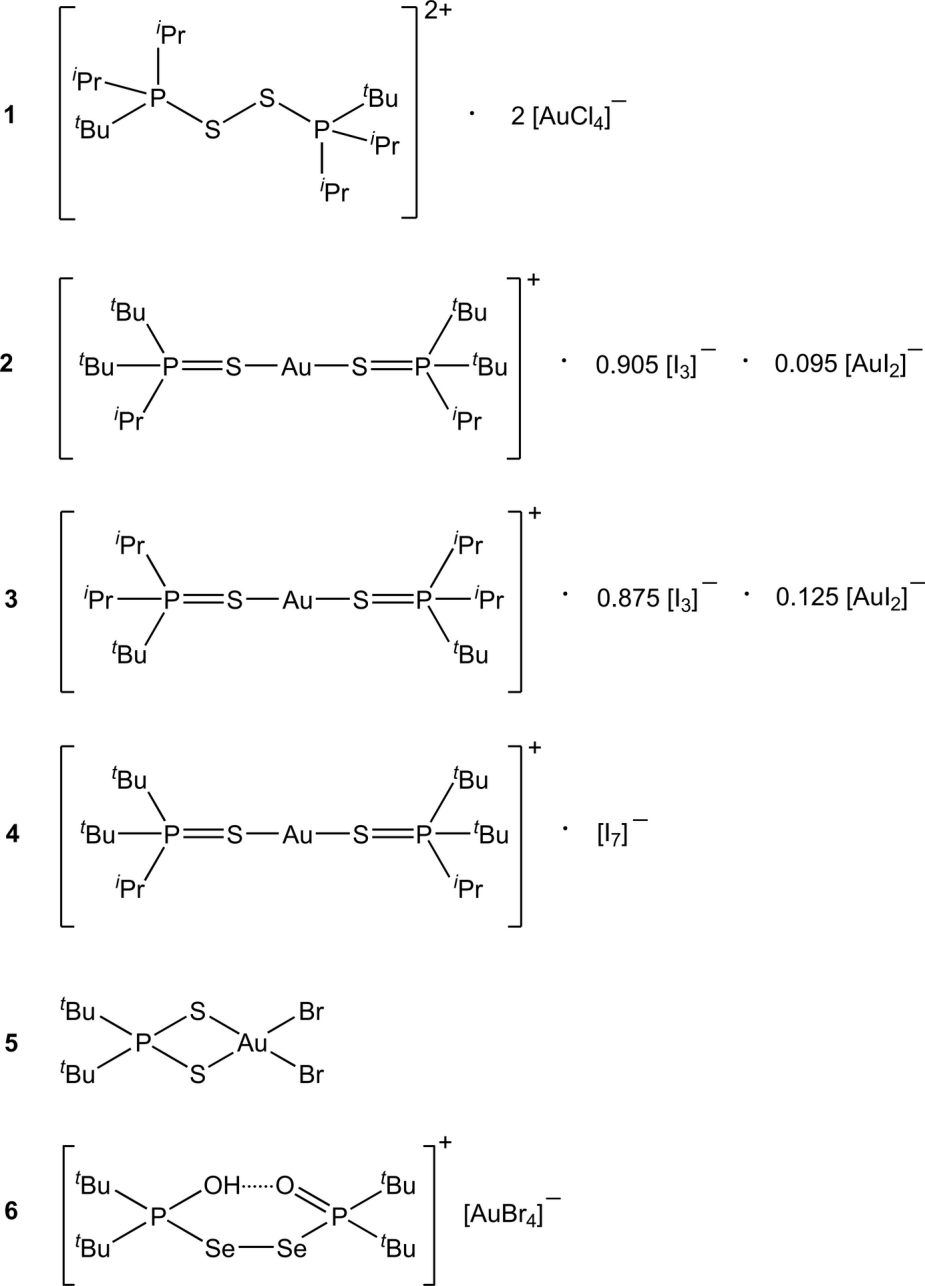


## 2. Structural commentary

**Figure 1:**
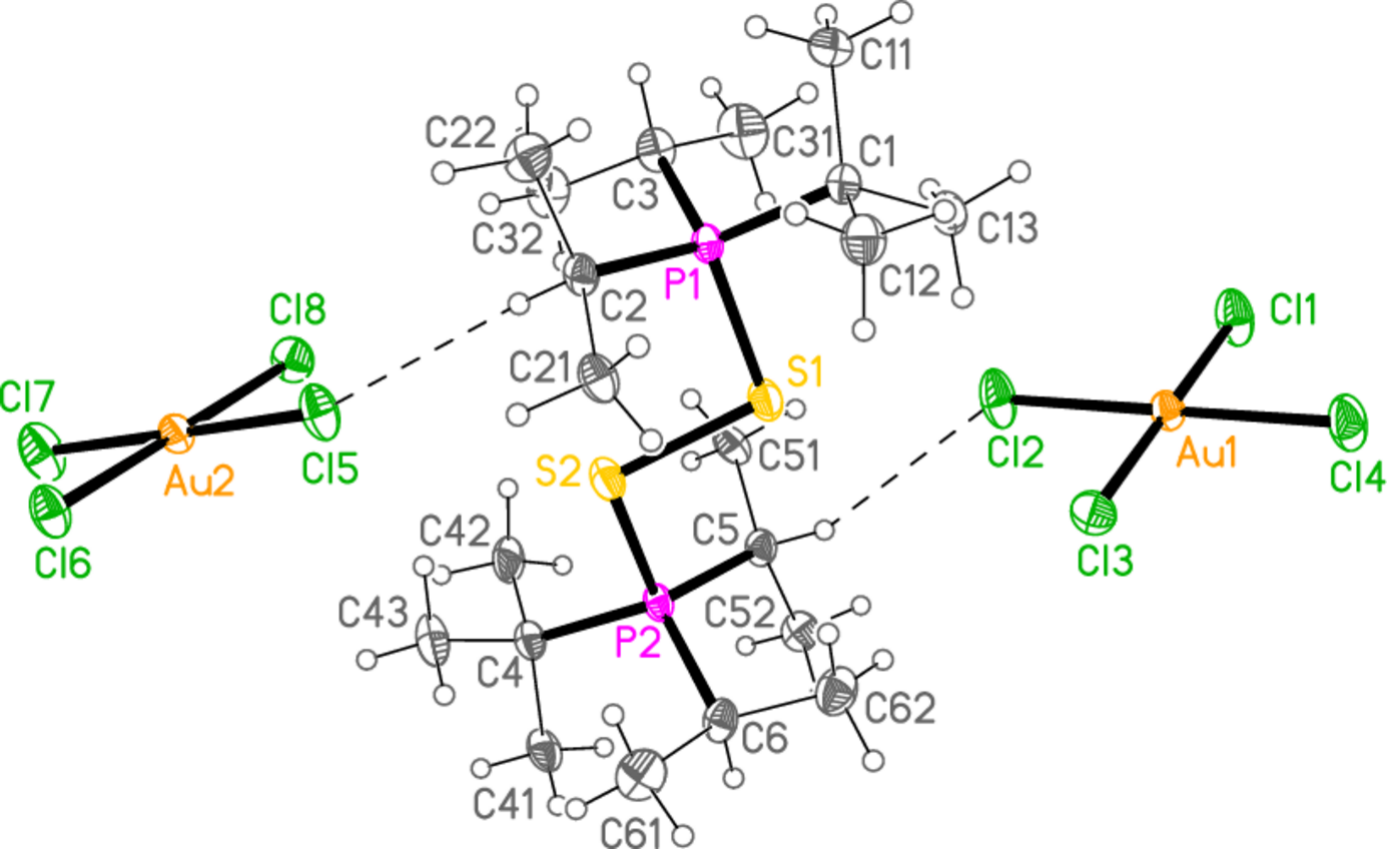
The structure of compound **1** in the crystal. The dashed lines indicate H⋯Cl contacts.

All compounds crystallized solvent-free. Selected mol­ecular dimensions are given in Tables 1 ▸–6 ▸ ▸ ▸ ▸ ▸. The structures are shown in Figs. 1 ▸–6 ▸ ▸ ▸ ▸ ▸, with ellipsoids at the 50% level. The short contacts shown in these figures are discussed in *Supra­molecular features*. For simplicity we write the P—*E* bonds in the text as single bonds, although they are often written as double bonds P=*E* in older literature (and indeed in the scheme). Primes (′) are used to denote generalized or previously defined symmetry operators.

The structure of compound **1**, bis­(*tert*-butyl­diiso­propyl­phospho­nium)­disulfane di-tetra­chloro­aurate(III) (an alternative name is used in the *Abstract*), (^*t*^Bu^*i*^Pr_2_P)_2_S_2_·[AuCl_4_]_2_, which crystallizes in space group *P*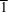 with *Z* = 2, is shown in Fig. 1 ▸. It corresponds to the addition of three halogen atoms per gold atom of the gold(I) precursor and thus completes the set of 1–4 electron oxidations of the gold(I) complexes [(*R*^1^*R*^2^*R*^3^P*E*)Au*X*]. This is the first isolated compound containing a dication of the form {(*R*_3_P)_2_*E*}_2_^2+^, but the compound {(Ph_3_P)_2_S}_2_(BF_4_)_2_ was identified in solution by Blankespoor *et al.* (1983 ▸). Bond lengths and angles in the P—S—S—P moiety may be considered normal, with P—S = 2.095 (2) and 2.097 (2), S—S = 2.121 (2) Å, and P—S—S angles of 99.30 (8) and 100.59 (8)°. The phospho­nium groups are anti­periplanar across the S—S bond, with a torsion angle of 165.51 (8)°; the sequences C1—P1—S1—S2 and C4—P2—S2—S1 are also anti­periplanar. The steric crowding of the bulky alkyl groups forces a close approach of some hydrogen atoms to the sulfur atoms, with H⋯S as short as 2.57 Å. These contacts are included in the table of hydrogen bonds (Table 7 ▸) for convenience, even if this does not reflect their true nature (similar considerations apply to short intra­molecular H⋯Au contacts for the gold derivatives discussed below).

**Figure 2:**
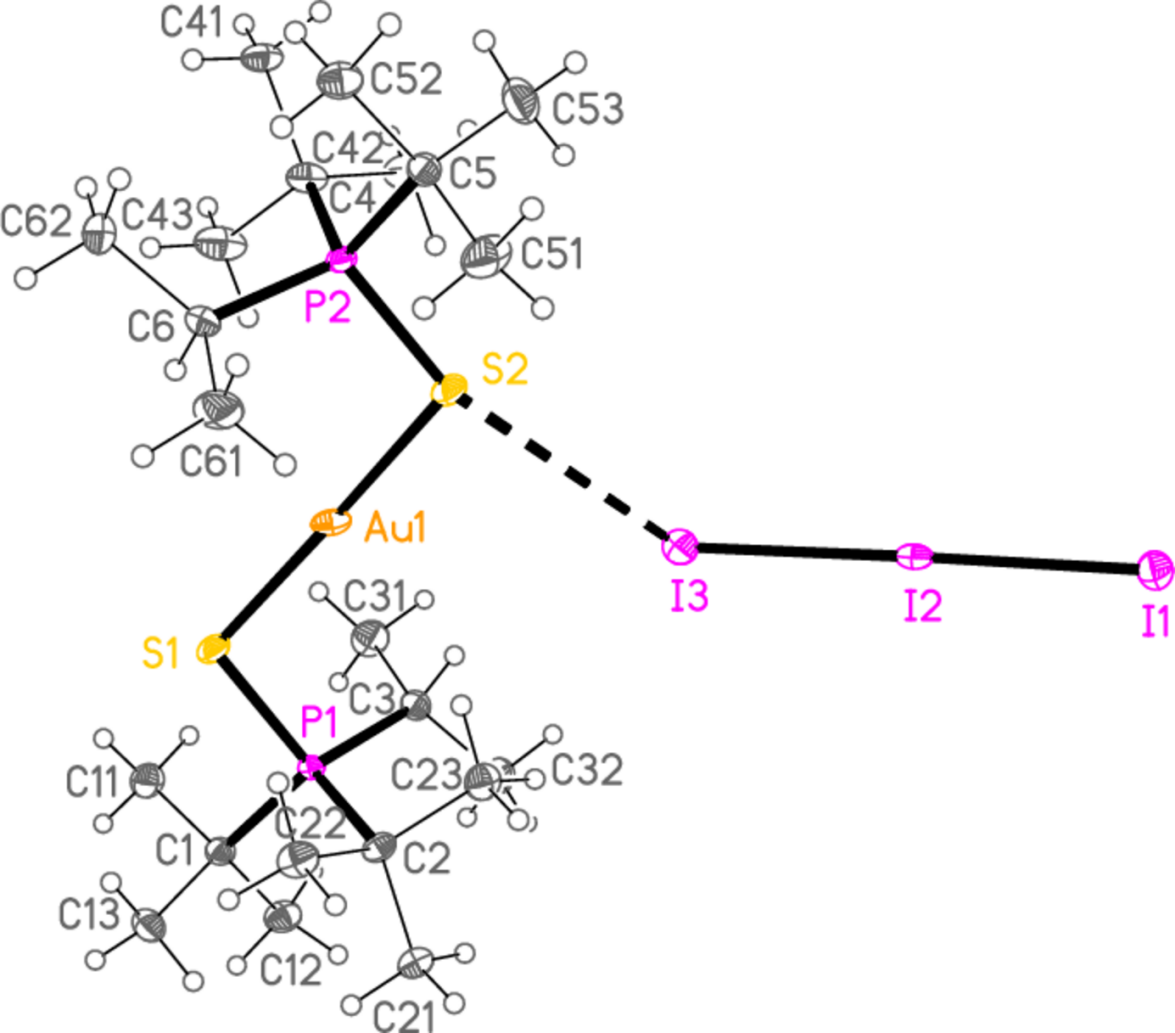
The structure of compound **2** in the crystal. The dashed line indicates an S⋯I contact. The minor disorder component (see text) is omitted.

**Figure 3:**
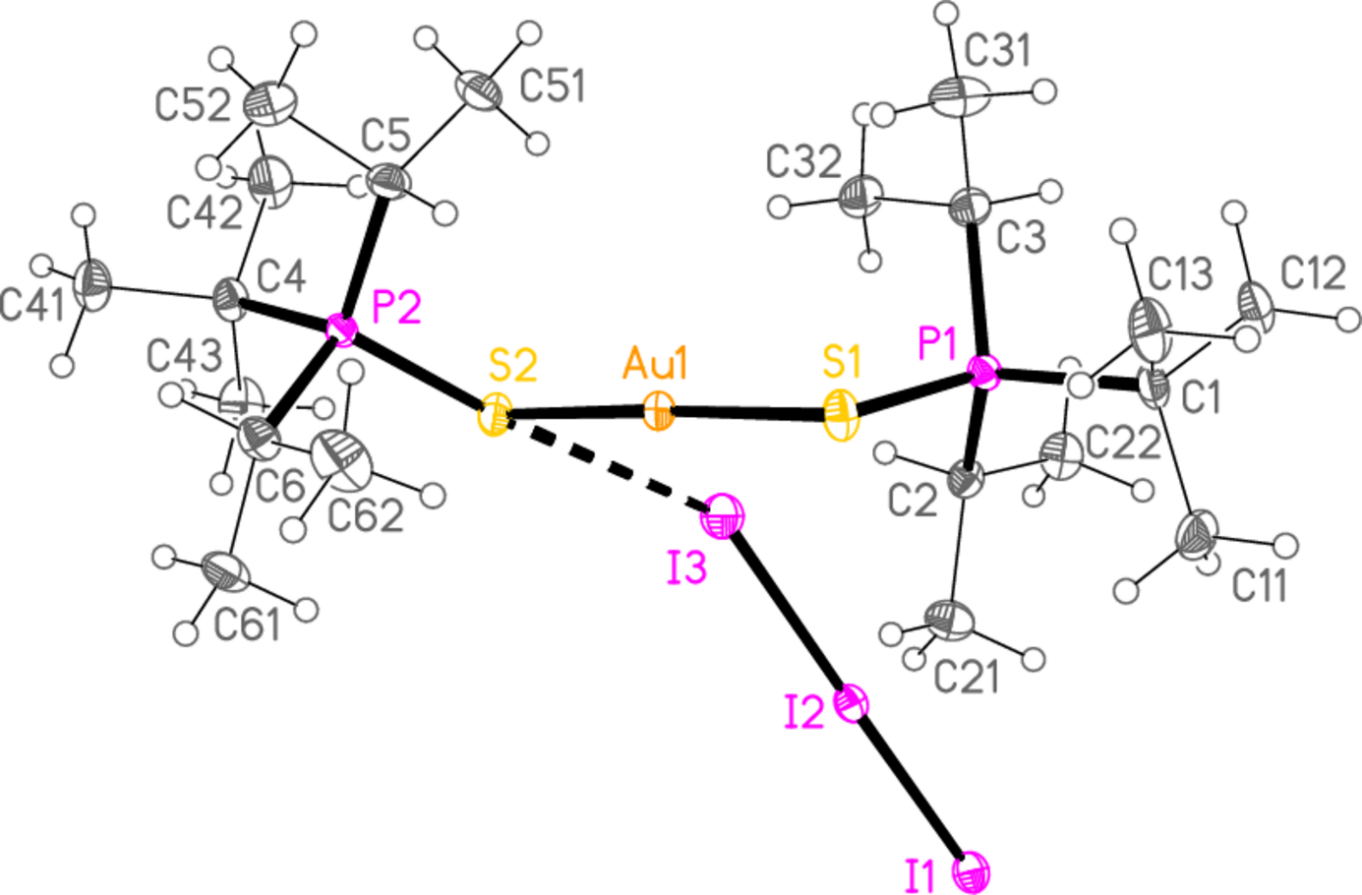
The structure of compound **3** in the crystal. The dashed line indicates an S⋯I contact. The minor disorder component (see text) is omitted.

**Figure 4:**
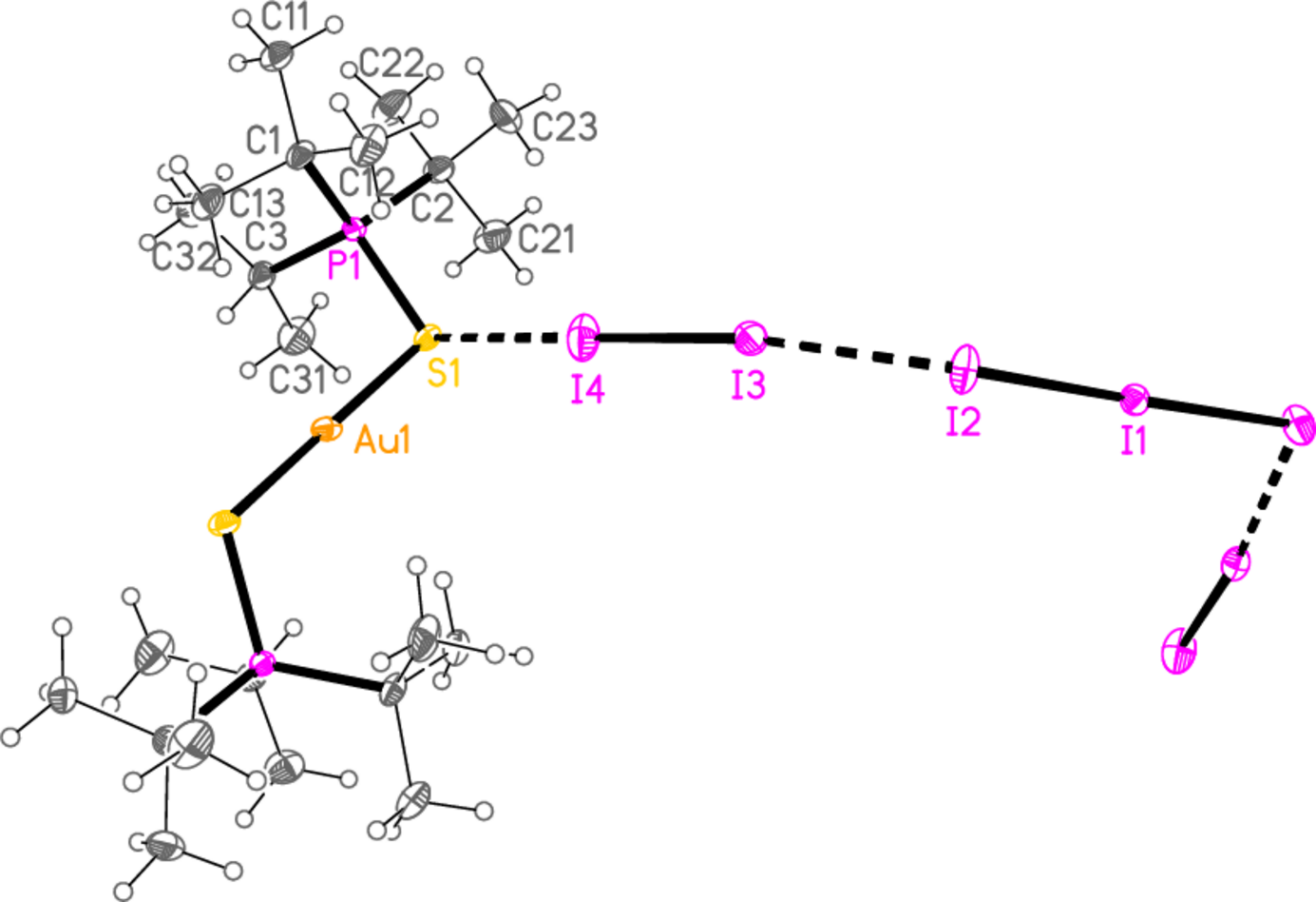
The structure of compound **4** in the crystal. The dashed lines indicate S⋯I contacts or the longer I—I bonds of the hepta­iodide.

Elemental iodine is generally not a sufficiently strong oxidizing agent to oxidize iodido­gold(I) complexes to tri­iodido­gold(III) complexes. Nevertheless, diiodine reacted with [(^*t*^Bu_2_^*i*^PrPS)AuI] to give a product that, based on the X-ray data (space group *P*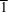, *Z* = 2), initially appeared to be the triiodide [(^*t*^Bu_2_^*i*^PrPS)_2_Au]I_3_; this is still formally an oxidation, but of the coordinated iodide (for which the oxidation number is increased from −1 to −1/3) rather than of the gold(I) centre. The refinement was at first unsatisfactory. A disorder model was then developed that involved replacement of 9.5% of the triiodide anion by the approximately isosteric di­iodo­aurate(I), corresponding to product **2**. We noted similar effects in the structures of Ph_3_PSAuI and Ph_3_PSAuI·0.5I_2_, which were contaminated by Ph_3_PS·I_2_ and Ph_3_PS·1.5I_2_ respectively (Taouss *et al.*, 2015 ▸). Fig. 2 ▸ shows the structure without its minor disorder component. The corresponding reaction of elemental iodine with [(^*t*^Bu^*i*^Pr_2_PS)AuI] led to the supposed triiodide [(^*t*^Bu^*i*^Pr_2_PS)_2_Au]I_3_, but again a small amount of triiodide (12.5%) was replaced by di­iodo­aurate(I) in the product **3** (space group *P*2_1_/*n*, *Z* = 4). Fig. 3 ▸ shows the structure without its minor disorder component. The bis-diiodine adduct of **3**, [(^*t*^Bu_2_^*i*^PrPS)_2_Au](I_7_) or [(^*t*^Bu_2_^*i*^PrPS)_2_Au]I_3_·2I_2_**4** (Fig. 4 ▸) was also obtained, which showed no contamination by di­iodo­aurate(I). It crystallizes in space group *Pccn* with *Z* = 4. The gold atom lies on a twofold axis (0.75, 0.25, *z*) and I1, the central atom of the hepta­iodide anion, on a twofold axis (0.75, 0.75, *z*).

The coordination geometry at the gold atoms of **2**–**4** is as expected linear. Bond lengths and angles between the heavier atoms (Å, °) are: Au—S = 2.2818 (5)–2.2990 (7), av. 2.2916; P—S = 2.0251 (13)–2.0444 (8), av. 2.0359; P—S—Au = 101.43 (4)–107.14 (3), av. 104.49. These compare well with the average values for analogous cations in the previous paper (Upmann *et al.*, 2024*d* ▸): Au—S = 2.2915, P—S = 2.0322 Å and P—S—Au = 104.05°, although the latter angle seems to be a ‘soft’ parameter that can vary appreciably. The ligands in **2** are almost ideally anti­periplanar across the S—Au—S moiety, with a torsion angle P—S⋯S—P of 179.21 (4)°. Structures **3** and **4** show appreciable deviation of this angle from 180°, with corres­ponding absolute torsion angles of 140.79 (5) and 143.88 (5)°, respectively. Dimensions of the linear triiodide components in **2**–**4** are normal, with bond lengths of 2.8754 (7)–2.9347 (2), av. 2.9151 Å.

The polyiodide region of compound **4** may be considered as an adduct of two diiodine mol­ecules with a triiodide ion, to form unbranched hepta­iodide (I_7_^−^) units of the idealized form I—I⋯I—I—I⋯I—I, bent at the atom I2 and its symmetry-equivalent. The bond lengths are I3—I4 = 2.7501 (3), I1—I2 = 2.9204 (2) and the longer I2⋯I3 = 3.2674 (2) Å (this latter distance is however less than the upper ‘Coppens limit’ for I—I bonds, see *Database survey)*. For comparison, the I—I bond length in solid diiodine at 100 K is 2.7179 (2) Å, as determined by Bertolotti *et al.* (2014 ▸) using a multipole refinement; this value is very close to the long-quoted 2.715 (6) Å of van Bolhuis *et al.* (1967 ▸). The torsion angle I3—I2⋯I2′—I3′ (omitting the central iodine I1) is −44.04 (1)°.

**Figure 5:**
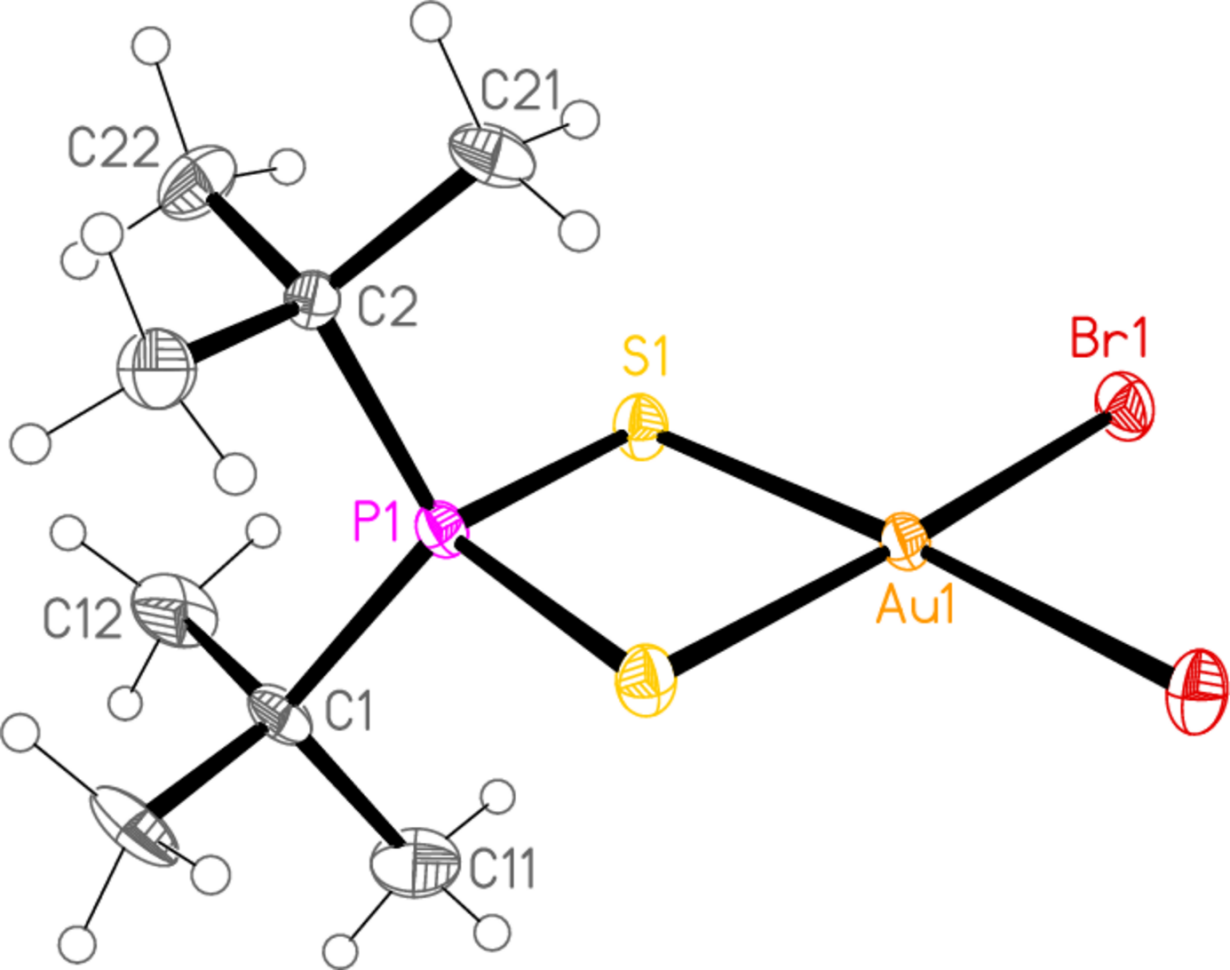
The structure of compound **5** in the crystal.

Compound **5**, di­bromido­(di-*tert*-butyl­dithio­phosphato-κ^2^*S*,*S*′)gold(III), [Au(^*t*^Bu_2_PS_2_)Br_2_], which contains a four-membered chelate ring (Fig. 5 ▸), was a minor product in the synthesis of ^*t*^Bu_3_PSAuBr (Upmann *et al.*, 2024*a* ▸). It crystallizes in space group *Pnma* with *Z* = 4. The gold and phospho­rus atoms, together with the carbon atoms C1, C2, C11 and C21, lie in the mirror plane at *y* = 0.25. One *tert*-butyl group per phospho­rus atom has been lost from the starting material. The atoms Au1, P1, S1, Br1, S1′ and Br1′ are approximately coplanar (r.m.s. deviation 0.04 Å).

**Figure 6:**
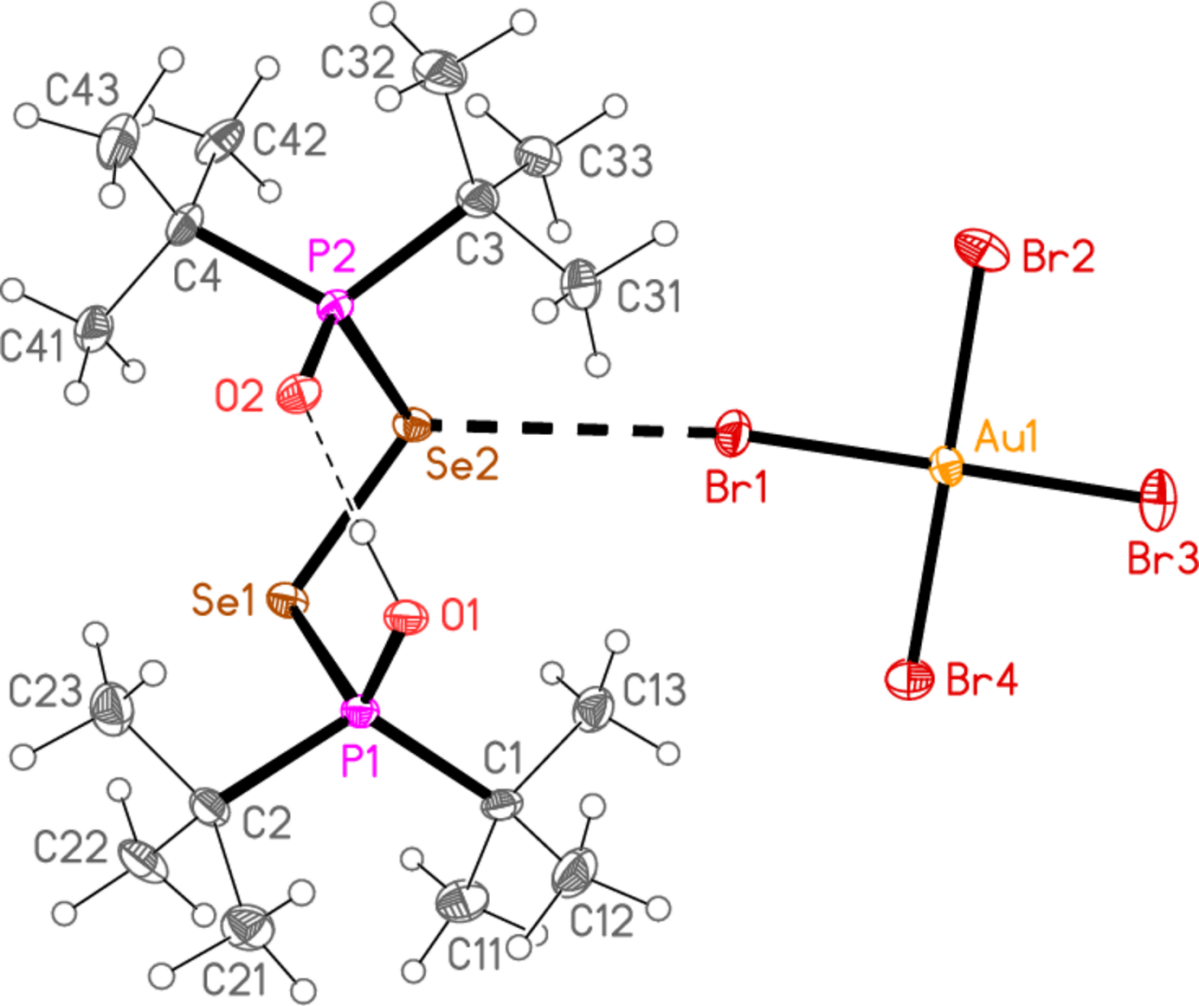
The structure of compound **6** in the crystal. The dashed lines indicate an Se⋯Br contact (thick) or an H⋯O hydrogen bond (thin).

Compound **6**, (bis­{(di-*tert*-but­yl)phosphine oxide} diselen­ide)hydrogen(I) tetra­bromido­aurate(III), [(^*t*^Bu_2_OPSe)_2_H][AuBr_4_], was a minor hydrolysis product in the synthesis of [(^*t*^Bu_3_PSe)_2_Au][AuBr_4_] (Upmann *et al.*, 2024*d* ▸). It crystallizes in space group *P*2_1_/*n* with *Z* = 4 (Fig. 6 ▸). Again, one *tert*-butyl group per phospho­rus atom has been lost from the starting material. The cation has a central diselenide unit, with Se1—Se2 = 2.3314 (6) Å and torsion angles P1—Se1—Se2—P2 = −71.86 (5), O1—P1—Se1—Se2 = 32.74 (14) and O2—P2—Se2—Se1 = 39.99 (14) °. It is uncertain if the intra­cationic hydrogen bond is symmetric, disordered or localized (see *Refinement*), but the P—O bond lengths are exactly equal.

## 3. Supra­molecular features

Tables 7 ▸–12 ▸ ▸ ▸ ▸ ▸ list short contacts that might be inter­preted as ‘weak’ hydrogen bonds; these include some borderline cases that are not further discussed. In the packing diagrams, the labelling denotes atoms of the asymmetric unit. Hydrogen atoms (and in some cases entire methyl groups) not involved in secondary inter­actions have been omitted for clarity.

**Figure 7:**
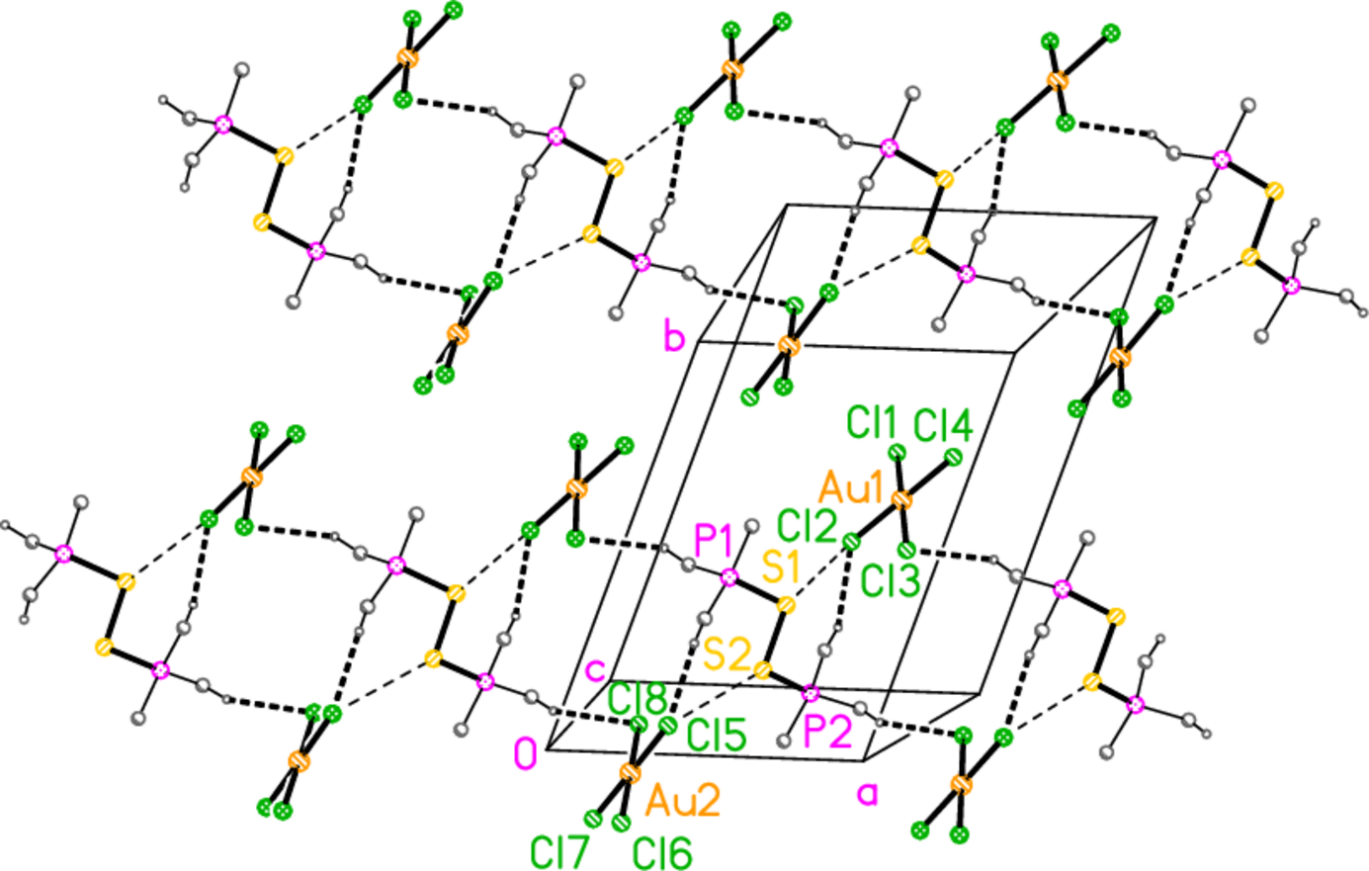
The packing of compound **1** viewed perpendicular to the *ab* plane in the region *z* ≃ 0.25, showing two ribbons parallel to the *a* axis. Dashed lines indicate hydrogen bonds H⋯Cl (thick) or S⋯Cl contacts (thin). Methyl groups are omitted for clarity.

In this series of publications, we have observed that the methine hydrogen atoms of isopropyl groups often act as donors in ‘weak’ hydrogen bonds of the type C—H⋯*X*. This is also the case for compound **1**, in which the atoms H2, H3, H5 and H6 form short contacts to the chlorine atoms of the anions, with H⋯Cl distances as short as 2.59 Å (Table 7 ▸); two of these, within the asymmetric unit, are shown in Fig. 1 ▸. There are also two short S⋯Cl contacts within the asymmetric unit (not shown explicitly in Fig. 1 ▸) namely S1⋯Cl2 = 3.757 (2) and S2⋯Cl5 = 3.691 (2) Å, with angles P1—S1⋯Cl2 = 120.15 (7), P2—S2⋯Cl5 = 132.16 (7), S1⋯Cl2—Au1 = 110.59 (6) and S2⋯Cl5—Au2 = 115.69 (6)°. These contacts combine to form infinite ribbons of residues parallel to the *a* axis (Fig. 7 ▸). The contact H6⋯Cl8′(1 + *x*, *y*, *z*) is also supported by H6⋯Au2′ (3.25 Å, same operator), which might be inter­preted as a C—H⋯Au hydrogen bond (Schmidbaur, 2019 ▸; Schmidbaur *et al.*, 2014 ▸).

**Figure 8:**
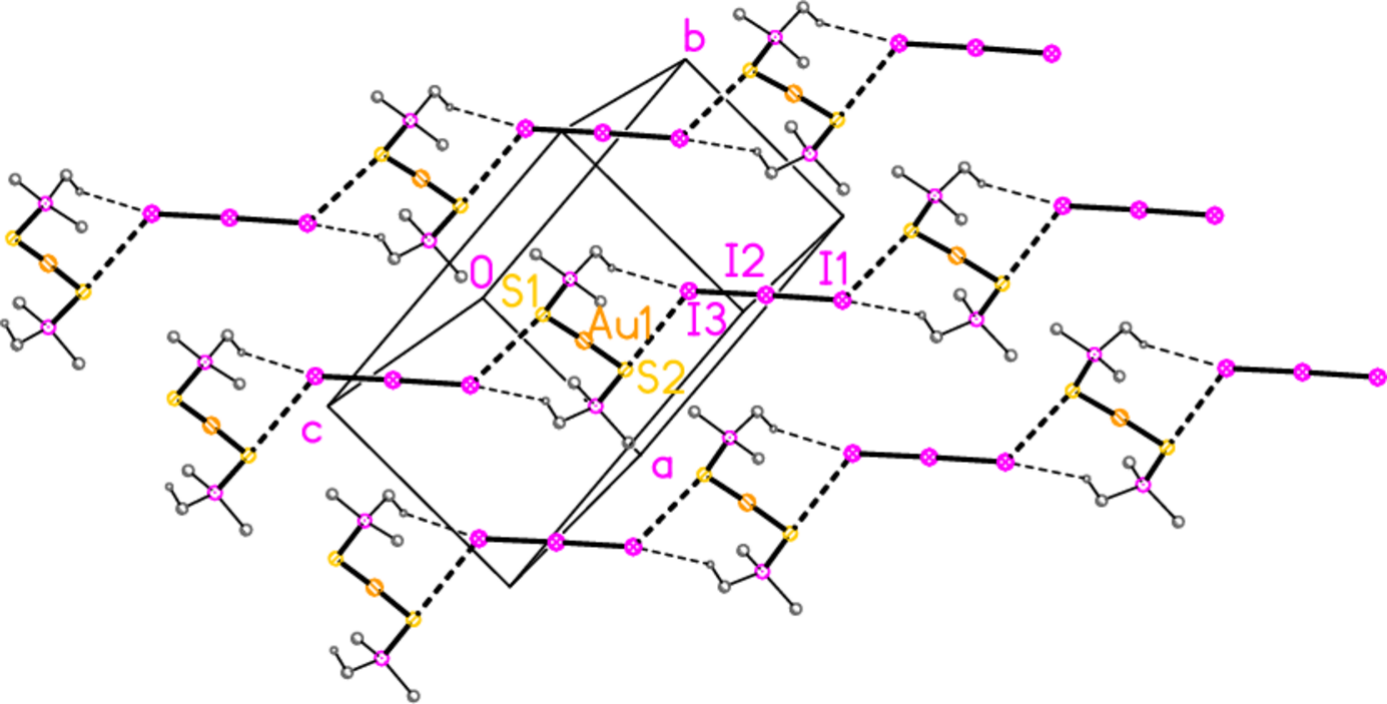
The packing of compound **2** viewed perpendicular to the *ab* plane in the region *z* ≃ 0.25, showing three chains of residues parallel to [110]. Dashed lines indicate S⋯I (thick) or H⋯I contacts (thin). Methyl groups are omitted for clarity.

**Figure 9:**
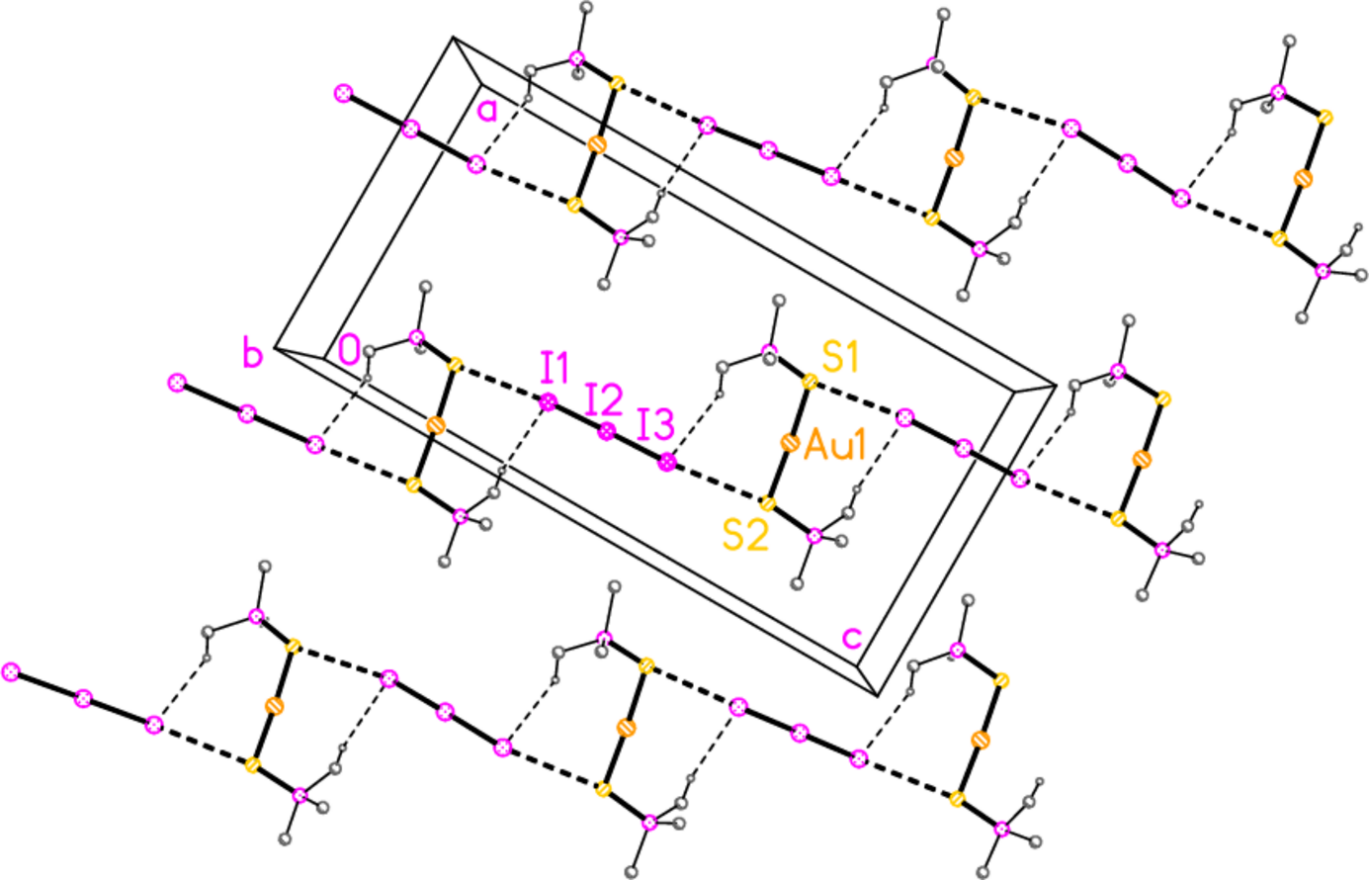
The packing of compound **3** viewed parallel to the *b* axis in the region *y* ≃ 0.25, showing three chains of residues parallel to [101]. Dashed lines indicate S⋯I (thick) or H⋯I contacts (thin). Methyl groups are omitted for clarity.

In the packing of compound **2**, infinite chains of alternating anions and cations parallel to [110] are formed *via* the contacts S2⋯I3 = 3.9230 (7) and S1⋯I1(−1 + *x*, −1 + *y*, *z*) = 3.9765 (7) Å, with angles P2—S2⋯I3 = 161.05 (3), P1—S1⋯I1′ = 161.16 (3), S2⋯I3—I2 = 125.84 (3) and S2⋯I1′—I2′ = 130.88 (3)°. Further support is provided by two C—H_methine_⋯I contacts (Fig. 8 ▸). The packing of compound **3** is similar, with chains parallel to [101] linked by S1⋯I1(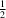 + *x*, 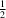 − *y*, 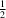 + *z*) = 3.9037 (11) and S2⋯I3 = 3.8366 (19) Å, with angles P1—S1⋯I1′ = 123.98 (4), P2—S2⋯I3 = 164.07 (5)°, S1⋯I1—I2′ = 120.95 (2) and S2⋯I3—I2 = 146.68 (5)°, also reinforced by two C—H_methine_⋯I contacts (Fig. 9 ▸).

**Figure 10:**
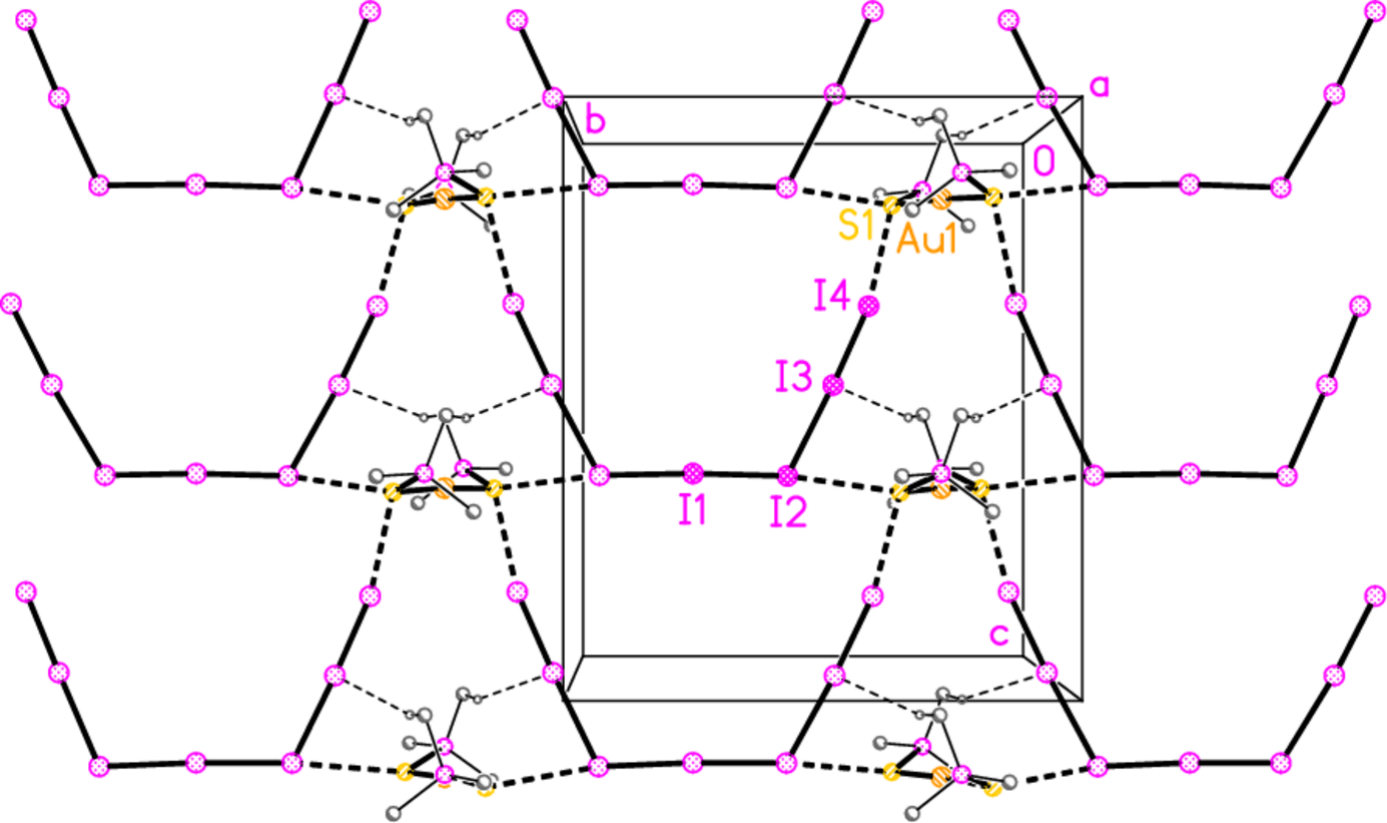
The packing of compound **4** viewed parallel to the *a* axis in the region *x* ≃ 0.75. Dashed lines indicate S⋯I (thick) or H⋯I contacts (thin). All I⋯I contacts are drawn as full bonds. Methyl groups are omitted for clarity.

The packing of compound **4** involves layers parallel to the *bc* plane (Fig. 10 ▸). Cations and anions are linked by the contacts S1⋯I4 = 3.3795 (7) and S1⋯I2(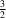 − *x*, *y*, −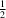 + *z*) = 3.6513 (7) Å, with angles P1—S1⋯I4 = 126.52 (3), P1—S1⋯I2′ = 136.64 (3), S1⋯I4—I3 = 168.85 (1), S1⋯I2′—I1′ = 165.01 (1), S1⋯I2′—I3′ = 78.91 (1)° and by one C—H_methine_⋯I contact.

The classification of short *E*⋯*X* contacts presents a problem: are they a particular type *X^D^*⋯*E^A^* of the well-known ‘halogen bonds’ *X^D^*⋯*A* (*D* = donor, *A* = acceptor; for a review see Metrangolo *et al.*, 2008 ▸), or a particular type *E^D^*⋯*X^A^* of the less well-known ‘chalcogen bonds’ *E^D^*⋯*A* (Aakeroy *et al.*, 2019 ▸; Vogel *et al.*, 2019 ▸)? The former involve halogens as donor atoms. The originally studied and perhaps best-known types are of the form C^*D*^—*X^D^*⋯*X^A^*—C^*A*^ (Pedireddi *et al.*, 1994 ▸), but the acceptor does not have to be a halogen. They have been explained in terms of inter­action between a positive hole (associated with the σ* orbital) in the extension of the C^*D*^—*X^D^* bond, and excess electron density at the acceptor *A* (which thus acts as a Lewis base); the C^*D*^—*X^D^*⋯*A* angles are *ca*. 180°. The latter involve chalcogens as donors, with a corresponding positive hole at the chalcogen atom, and, in the concrete case of phosphane chalcogenide adducts, should result in a P—*E*⋯*A* angle of *ca*. 180°. Thus Hasija & Chopra (2020 ▸), in their analysis of five adducts/co-crystals of phosphane sulfides with iodo­benzenes, found that four of these displayed halogen bonds C—I⋯S—P (where the order of the atoms implies that the first group is the donor and the second group the acceptor), all with C—I⋯S angles approximately linear and I⋯S—P angles approximately tetra­hedral, whereas one contained two chalcogen bonds of the type P—S⋯I—C, with P—S⋯I angles approximately linear and S⋯I—C angles approximately tetra­hedral. It is inter­esting that the directionality of phosphine sulfides as halogen-bond acceptors corresponds roughly to the lone-pair directions at the sulfur atom in the simple Lewis formulae. Similarly, Ishigaki *et al.* (2022 ▸), in their studies of nine 4,7-dihalobenzo[*c*]chalcogena­diazo­les, divided these into halogen-bond- or chalcogen-bond-dominated structures. We used similar angle criteria, in particular the linearity at *E*, to suggest chalcogen bonding P—*E*⋯Cl—Au in three chloro­chalcogenyl­phospho­nium tetra­chloro­aurates(III) (*R*^1^*R*^2^*R*^3^P*E*Cl)^+^ [AuCl_4_]^−^ (Upmann *et al.*, 2024*c* ▸). The angle criteria for **1** are not clear-cut. For **2** and **3** three of four P—S⋯I are linear but S⋯I—I are not, suggesting chalcogen bonding, and for **4** the S⋯I—I angles are linear, suggesting halogen bonding. However, it should be noted that the contact distances are much greater for **2** and **3**, so that it may not be appropriate to seek definite categories for these contacts.

Resnati and co-workers have introduced the term ‘coinage bond’ to describe inter­actions between classical donor atoms *L*, bearing lone pairs, and Au^III^ centres; these inter­actions involve short contacts *L*⋯Au to the gold atom at positions that complete a distorted octa­hedral geometry (*i.e.* the additional contacts lie above and below the gold atom, perpend­ic­ular to the ligand plane, at distances much longer than normal bonds). Previously these were often, somewhat simplistically, rationalized as contacts facilitated by the easy steric access in those directions. Daolio *et al.* (2021 ▸) and Pizzi *et al.* (2022 ▸) calculated the presence of π-holes at the gold atom at precisely these positions, which can then inter­act with excess negative charge at the atoms *L*, giving rise to the additional contacts. In this type of inter­action the gold atom is the coinage-bond donor, whereas the atom *L* is the coinage bond acceptor. The same model was used to explain the short Au⋯Cl contacts between neighbouring tetra­chloro­aurate(III) anions, as observed in numerous structures. This shows, however, that the behaviour of the halogen atoms *X* in [Au*X*_4_]^−^ ions, when these form short contacts involving their halogen atoms, could theoretically be of two alternative and opposite types: (i) a coinage or chalcogen bond (or other related types), in which the atom *X* is an electron donor and coinage/chalcogen bond acceptor, or (ii) a halogen bond, in which the atom *X* is a halogen bond donor and electron acceptor. Detailed calculations might be necessary to determine the nature of the inter­action(s) for any particular case.

**Figure 11:**
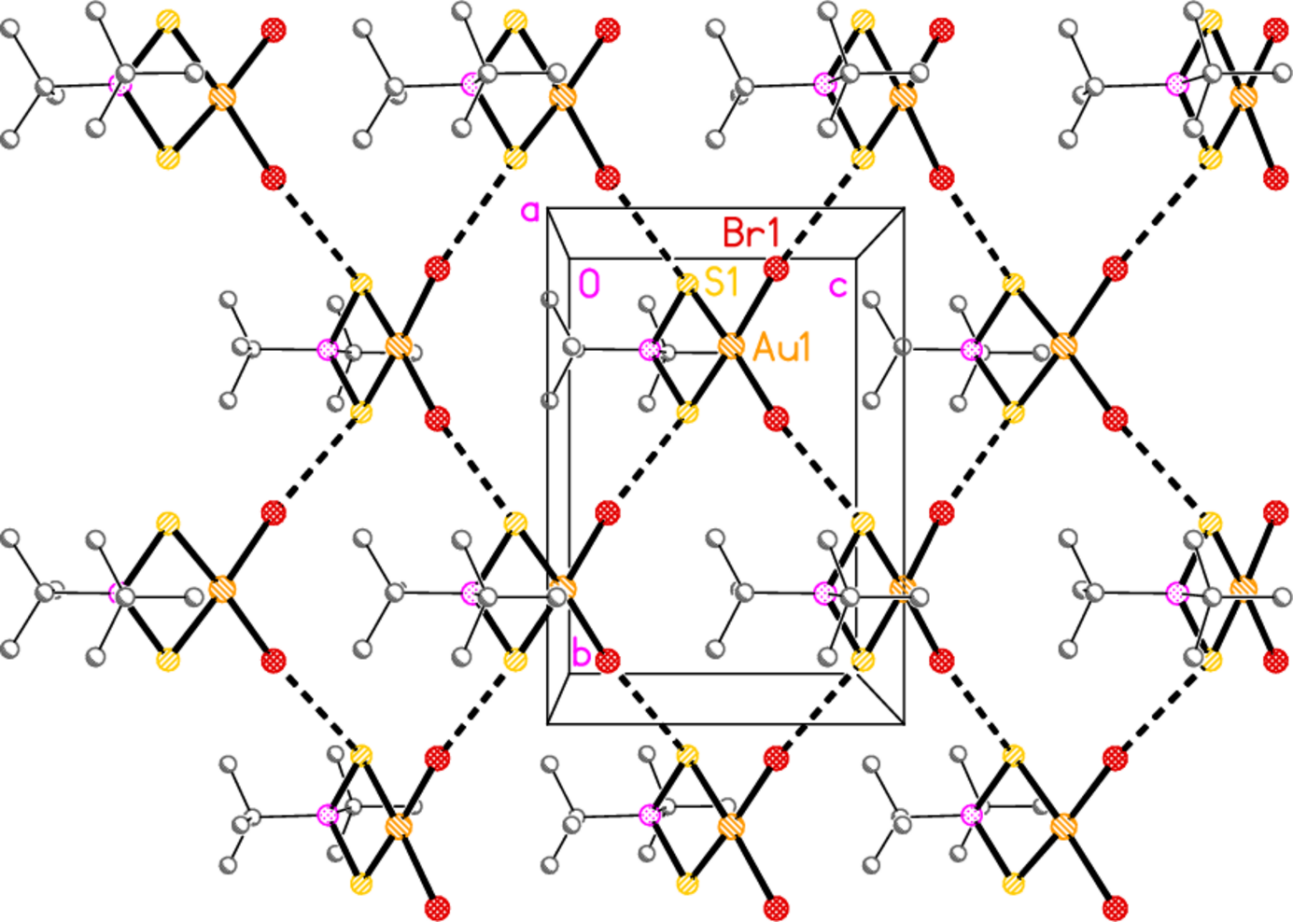
The packing of compound **5** viewed parallel to the *a* axis in the region *x* ≃ 0.75. Dashed lines indicate S⋯Br contacts.

**Figure 12:**
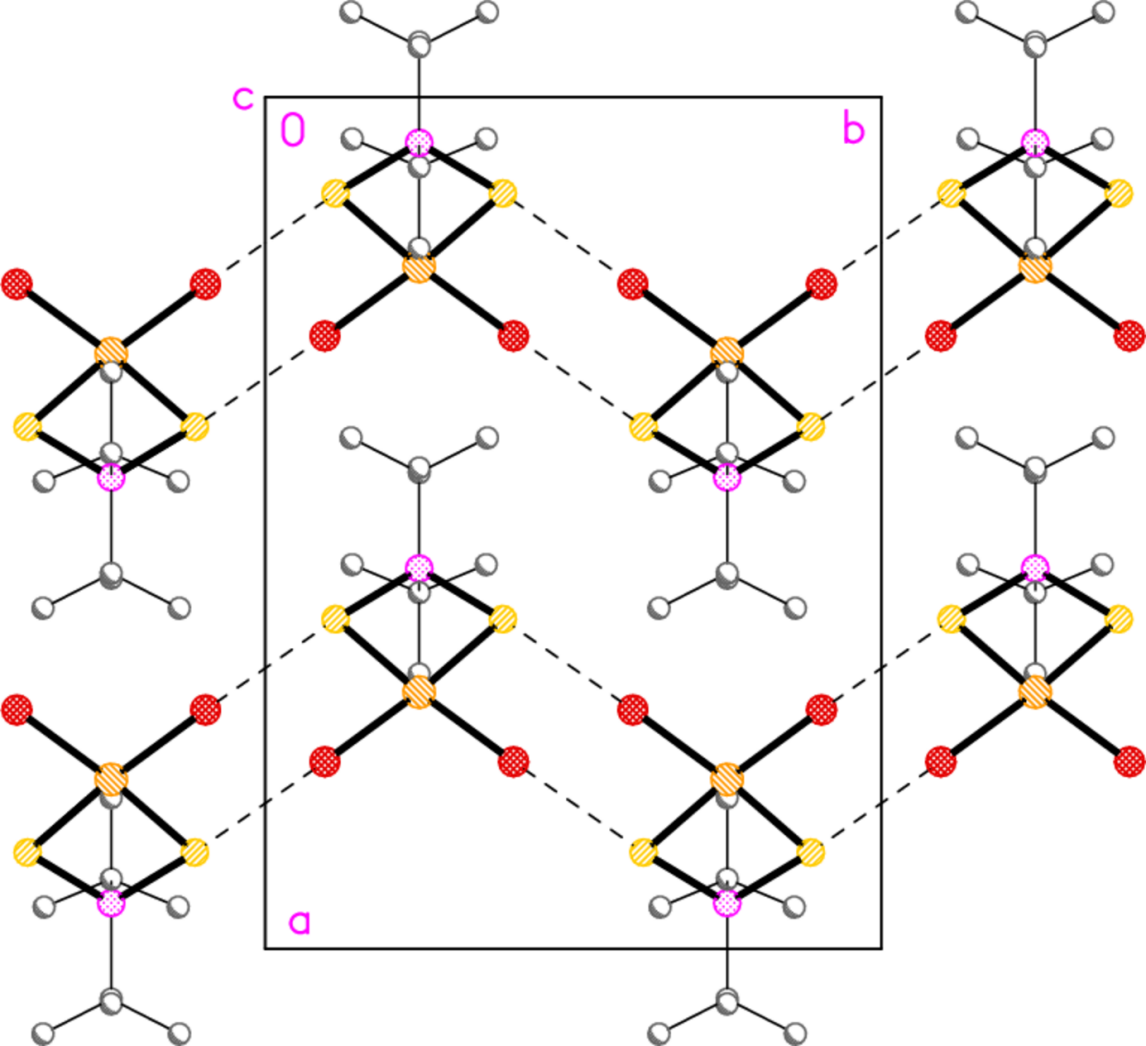
The packing of compound **5** projected parallel to the *c* axis to show the corrugation of the layers. Dashed lines indicate S⋯Br contacts.

In compound **5**, the contact S1⋯Br1(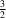 − *x*, −*y*, −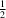 + *z*) = 3.5555 (12) Å, with angles P1—S1⋯Br1′ = 120.15 (6) and S1⋯Br1′—Au1′ = 172.02 (3)°, links the mol­ecules to form corrugated layers of mol­ecules parallel to the *bc* plane (Fig. 11 ▸). The angles strongly suggest that the contact is a halogen bond. The corrugation is shown clearly in the projection parallel to the *c* axis (Fig. 12 ▸).

**Figure 13:**
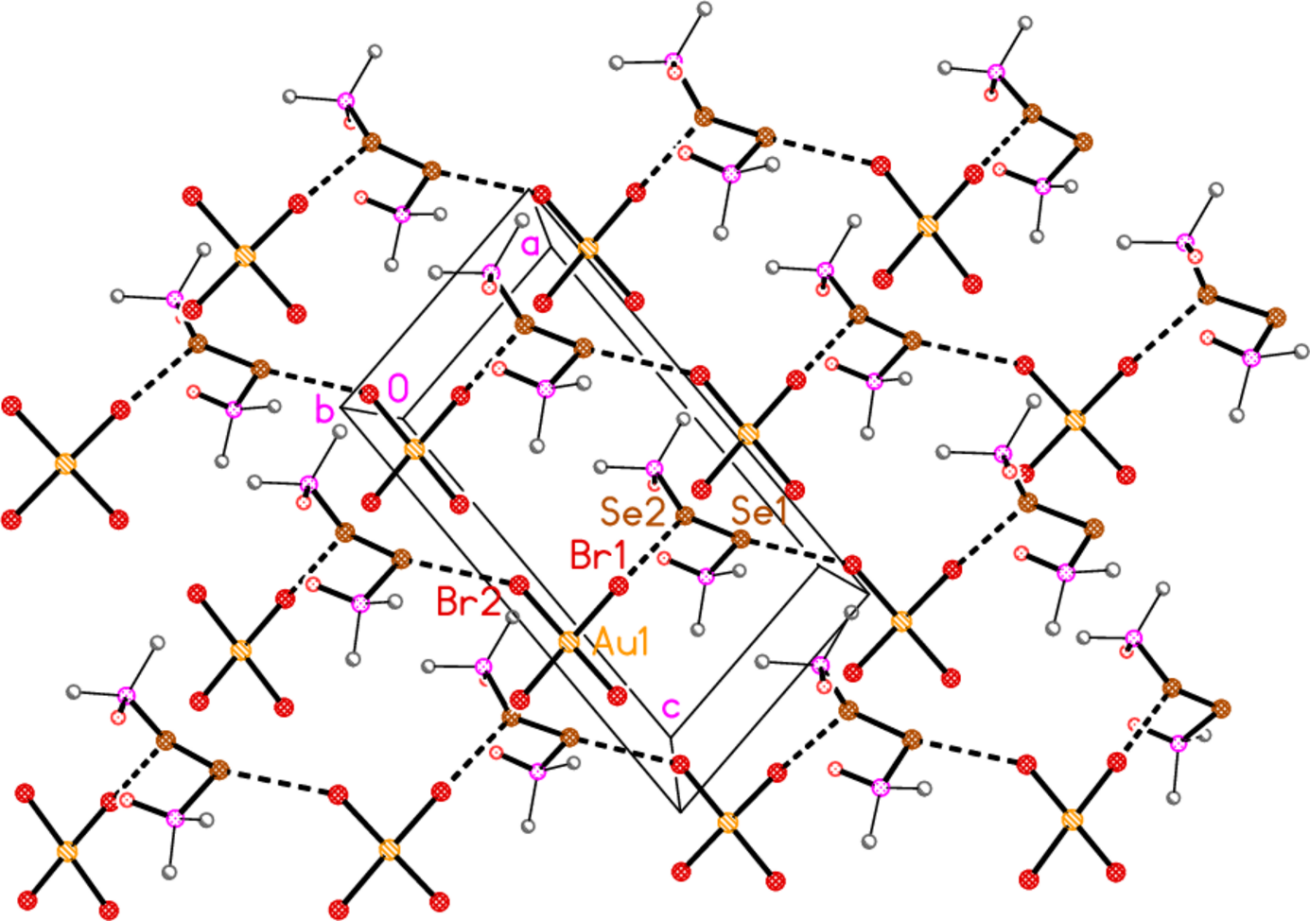
The packing of compound **6** viewed parallel to the *b* axis in the region *y* ≃ 0.75. Dashed lines indicate Se⋯Br contacts. Methyl groups and intra­cationic hydrogen bonds are omitted for clarity. The four zigzag chains of alternating cations and anions run horizontally.

In compound **6**, the contacts Se1⋯Br2(1 + *x*, 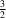 − *y*, 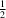 + *z*) = 3.6631 (7) and Se2⋯Br1 = 3.6047 (6) Å, with corresponding P—Se⋯Br angles of 115.34 (4) and 119.61 (3)° and Se⋯Br—Au angles of 167.06 (2) and 143.08 (2)°, combine to form zigzag chains of alternating cations and anions parallel to [201] (Fig. 13 ▸).

## 4. Database survey

The searches employed the routine ConQuest (Bruno *et al.*, 2002 ▸), part of Version 2024.1.0 of the Cambridge Database (Groom *et al.*, 2016 ▸).

A search for bis-phospho­nium disulfanes analogous to **1** gave no hits, but one analogous tris­ulfane was found, namely 1,3-bis­(triiso­butyl­phospho­nium)­tris­ulfane bis­[nona­chloro­oxodiniobium(IV)] (Stumpf *et al.*, 1999 ▸; refcode GOQZAN). This has bond lengths P—S = 2.123 (3) and 2.011 (3), S—S = 2.064 (3) and 2.107 (3) Å, with appreciable differences between chemically equivalent bond lengths, and bond angles P—S—S = 103.79 (11) and 105.00 (14), S—S—S = 107.19 (15)°.

One of the S⋯I contacts in compound **4** is markedly short at 3.3795 (7) Å. A search for short contacts P—S⋯I (with the S atom defined as 1-coordinate) found the shortest value to be 3.163 (2) Å for the 1:1 complex between Ph_3_PS and 1,3,5-tri­fluoro­tri­iodo­benzene (Hasija & Chopra, 2020 ▸; RUWVEN), whereas the shortest contact involving diiodine (or polyiodides) is 3.204 (6) Å for {(Ph_2_P(=S)}_2_CH_2_·I_2_ (Apperley *et al.*, 2001 ▸; NENRON).

A search for the triiodide anion gave 1734 hits, with bond lengths (excluding one extreme outlier) 2.741–3.312 Å, av. 2.92 (5) Å. The review of polyiodides by Svensson & Kloo (2003 ▸) can be consulted for a more extensive overview. More recently, Savastano *et al.* (2022 ▸) have presented a detailed analysis of polyiodides in the database, and recommended extending the ‘Coppens limit’ of 3.3 Å (Coppens, 1982 ▸), the long-accepted dividing value between I—I bonds and I⋯I contacts, to the range 3.4–3.5 Å. For comparison, the shortest non-bonded distance in elemental diiodine is 3.5010 (2) Å (Bertolotti *et al.*, 2014 ▸).

There are several hepta­iodide structures in the literature, generally inter­pretable, as in compound **4**, as associations of a triiodide ion with two diiodine mol­ecules; a typical example (Walbaum *et al.*, 2010 ▸; refcode XAGJUM) involves a strontium crown ether complex as cation. Its hepta­iodide bond lengths and angles are similar to those of **4**, but the absolute torsion angle corresponding to the 44.04 (1)° for I3—I2⋯I2′—I3′ in **4** is 89.6°. Furthermore, the hepta­iodides are further linked by I⋯I contacts of 3.5–3.6 Å, whereas those of **4** are isolated (except for H⋯I contacts).

**Figure 14:**
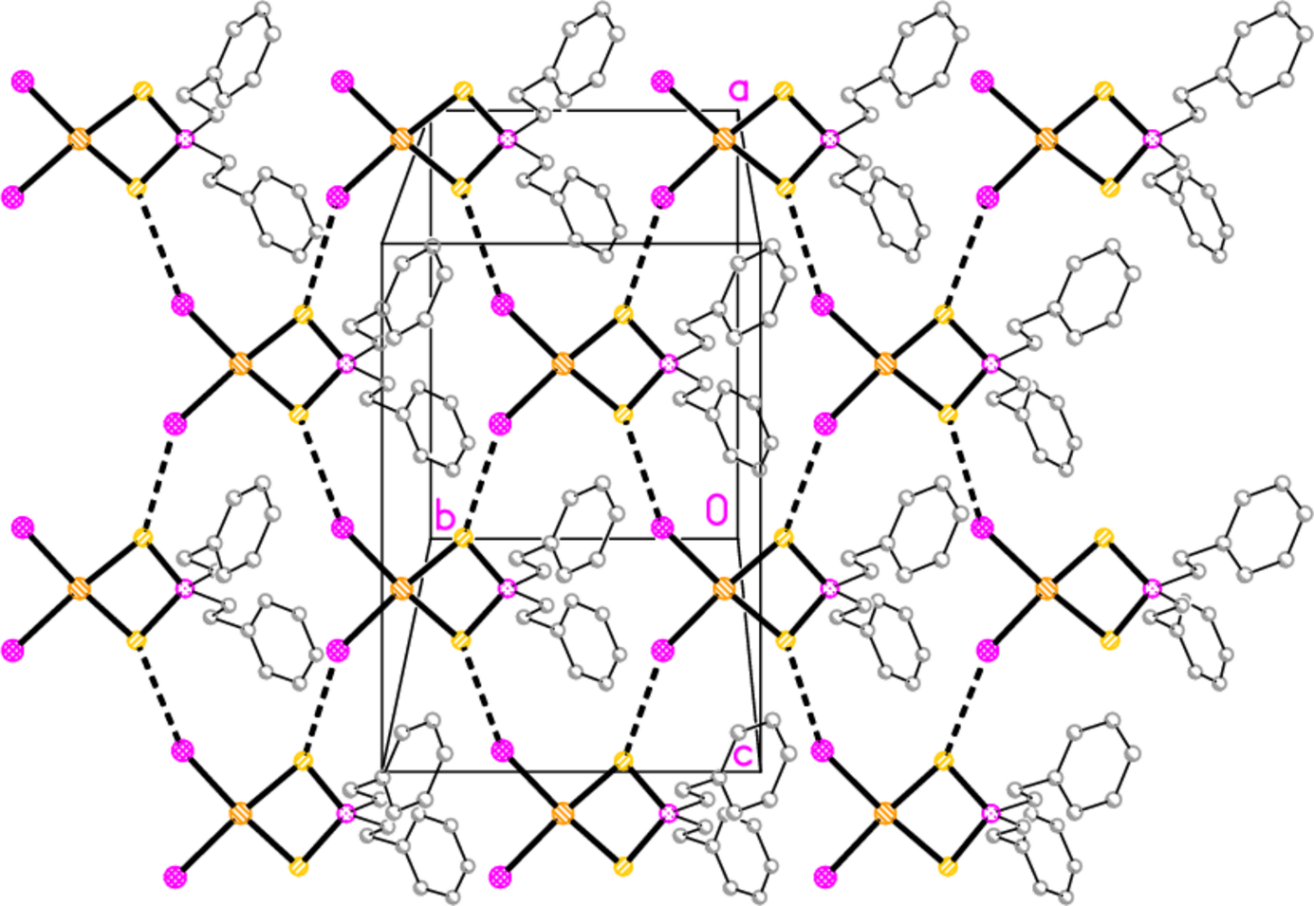
The packing of di­iodido­{bis­(phenyl­eth­yl)di­thio­phosphato-κ^2^*S*,*S*′}gold(III) (NIMMED01; Lee *et al.*, 2014 ▸) viewed perpendicular to the *ab* plane in the region *z* ≃ 0.25. Dashed lines indicate S⋯I contacts. The diagram was drawn using the deposited coordinates, translated by (−0.5, −0.5, −0.5).

A search for the grouping C_2_P*E*_2_Au*X*_2_ with the same general structure as **5** (involving a four-membered chelate ring) gave only two hits: the homologous, but non-isotypic, compounds di­iodido­{bis­(phenyl­eth­yl)di­thio­phosphato-κ^2^*S*,*S*′}gold(III) and di­iodido­{bis­(phenyl­eth­yl)di­seleno­phosphato-κ^2^*Se*,*Se*′}gold(III), produced by the action of elemental iodine on the dimeric gold(I) precursors (NIMMED01 and WIRGUB; Lee *et al.*, 2014 ▸). Clearly the special nature of these gold(I) dimers in some way facilitates their oxidation by iodine. They are also well-known for their ready oxidation to dinuclear gold(II) derivatives, see *e.g.* BIBPIL (Fackler & Basil, 1982 ▸) or EMPLAU (Schmidbaur *et al.*, 1976 ▸), some of the earliest examples of this type of compound. The packing of the selenium derivative involves one-dimensional association of the mol­ecules via Se⋯Se and Se⋯I contacts, and a corresponding packing diagram was presented. However, the packing of the sulfur derivative was not analysed, so we present it here. The compound crystallizes in *C*2/*c* with imposed twofold symmetry. The S⋯I′ contact of 3.714 Å (with angles P—S⋯I′ = 109.7 and S⋯I′—Au′ = 135.3°) links the mol­ecules to form layers parallel to the *ab* plane (Fig. 14 ▸).

A search for compounds of the form (*O*,*C*,*C*)PSeSeP(*C*,*C*,*O*) analogous to **6** was unsuccessful. Allowing any chalcogen *E′* rather than just oxygen gave six hits. Refcode ETPOSE (Husebye, 1966 ▸) has twofold symmetry, *E′* = S and four ethyl groups; KIHGIT (a 1:1 adduct/co-crystal with the monoselenide) and XETSUM (the diselenide alone) with *E′* = Se and four cyclo­hexyl groups (Artem’ev *et al.*, 2013*a* ▸,*b* ▸); NEJNIA (Nguyen *et al.*, 2006 ▸) with twofold symmetry, *E′* = Se and four isopropyl groups; NOSVIA (Potrzebowski *et al.*, 1997 ▸) with twofold symmetry, *E′* = S, two phenyl and two *tert*-butyl groups (N.B. in the original paper, the intra­molecular symmetry operator is given incorrectly as an inversion; it should be 1 − *x*, *y*, 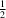 − *z*); and XETTAT with *E′* = S and four phenyl­ethyl groups (Artem’ev *et al.*, 2013*b* ▸). For the selenium derivatives, the Se—Se bond lengths lie in the range 2.275–2.384 Å and the central absolute torsion angles in the wide range 91.99° (NOSVIA) to 146.34° (XETTAT).

## 5. Synthesis and crystallization

Most of the compounds arose as minor products. Compound **1** was identified as a few crystals of different habit during the synthesis of [(^*t*^Bu^*i*^Pr_2_PS)_2_Au][AuCl_4_] (Upmann *et al.*, 2024*d* ▸). The polyiodides **2**–**4** were synthesized in pilot experiments by the addition of small qu­anti­ties of elemental iodine to the iodido­gold(I) precursors in di­chloro­methane, followed by overlayering with *n*-pentane; unsurprisingly, no oxidation of the gold centres resulted, and the experiments were not followed up. Reactions did however take place, and the products were structurally inter­esting. Compound **5** arose as a crystalline impurity in the synthesis of ^*t*^Bu_3_PSAuBr (Upmann *et al.*, 2024*a* ▸). Compound **6**, a hydrolysis product, was a crystalline impurity in the synthesis of [(^*t*^Bu_3_PSe)_2_Au][AuBr_4_] (Upmann *et al.*, 2024*d* ▸).

## 6. Refinement

Details of the measurements and refinements are given in Table 13 ▸. Structures were refined anisotropically on *F*^2^. The OH hydrogen atom of compound **6** was refined freely, but it has a rather high *U* value of 0.10 (3). This, together with the equal P—O bond lengths (which should be significantly different for localized P—OH and P=O groups), may indicate some disorder of this hydrogen atom, possibly involving some contribution with the hydrogen atom equidistant between the two oxygen atoms. Methine hydrogens were included at calculated positions and refined using a riding model with C—H 1.00 Å and *U*(H) = 1.2 × *U*_eq_(C). Methyl groups were refined, using the command ‘AFIX 137′, as idealized rigid groups allowed to rotate but not tip, with C—H = 0.98 Å, H—C—H = 109.5° and *U*(H) = 1.5 × *U*_eq_(C). This procedure is less reliable for heavy-atom structures, so that any postulated hydrogen bonds involving methyl hydrogen atoms should be inter­preted with caution.

*Exceptions and special features*: The structure of compound **2**, initially thought to be a simple triiodide salt, could not at first be refined satisfactorily; the *R* values were high, and the triiodide region contained appreciable residual electron density. This was inter­preted as a small amount of di­iodido­aurate(I) overlaid on the (approximately isosteric) triiodide anion site, and a disorder model was accordingly refined. The occupation factor of the di­iodido­aurate(I) component refined to 0.0948 (15). The structure of compound **3** was treated as a pseudo-merohedral twin (by 180° rotation about the *a* axis); the relative volume of the smaller twin component refined to 0.1602 (3). A similar disorder to that of **2** was observed and was treated in the same way; the occupation factor of the di­iodido­aurate(I) component refined to 0.1252 (15). The dimensions of disordered groups should always be inter­preted with caution, and we do not discuss the minor disorder components further, nor are they included in the Figures. For compound **4**, an extinction correction (Sheldrick, 2015 ▸) was applied; the extinction parameter refined to 0.000131 (6). For compound **5**, where the methyl carbon atoms C11 and C21 lie in mirror planes, the ‘AFIX 137’ command for these atoms placed one methyl hydrogen atom close to, but not in, the mirror plane, which would imply slightly disordered methyl groups (maxima for the hydrogens at C11 were clearly recognisable, but for C21 less so). For this reason, the program *XP* (Bruker, 1998 ▸) was used to generate idealized hydrogen positions at C11 and C21 with the ‘HADD 3’ command; these were close to the hydrogen positions found using ‘HFIX 137’. Two methyl hydrogens (one in the mirror plane, one general) at C11 and C21 were then refined with ‘AFIX 3’, riding on their methyl carbon atoms.

## Supplementary Material

Crystal structure: contains datablock(s) 1, 2, 3, 4, 5, 6, global. DOI: 10.1107/S2056989024010788/yz2060sup1.cif

Structure factors: contains datablock(s) 1. DOI: 10.1107/S2056989024010788/yz20601sup2.hkl

Structure factors: contains datablock(s) 2. DOI: 10.1107/S2056989024010788/yz20602sup3.hkl

Structure factors: contains datablock(s) 3. DOI: 10.1107/S2056989024010788/yz20603sup4.hkl

Structure factors: contains datablock(s) 4. DOI: 10.1107/S2056989024010788/yz20604sup5.hkl

Structure factors: contains datablock(s) 5. DOI: 10.1107/S2056989024010788/yz20605sup6.hkl

Structure factors: contains datablock(s) 6. DOI: 10.1107/S2056989024010788/yz20606sup7.hkl

CCDC references: 2156794, 2401167, 2156891, 2401166, 2156889, 2401165

Additional supporting information:  crystallographic information; 3D view; checkCIF report

## Figures and Tables

**Table 1:** Selected geometric parameters (Å, °) for **1**[Chem scheme1]

S1—P1	2.095 (2)	S2—P2	2.097 (2)
S1—S2	2.121 (2)		
			
P1—S1—S2	99.30 (8)	P2—S2—S1	100.59 (8)
			
P1—S1—S2—P2	165.51 (8)	S1—S2—P2—C5	−38.6 (2)
S2—S1—P1—C2	39.1 (2)	S1—S2—P2—C6	78.7 (2)
S2—S1—P1—C3	−78.5 (2)	S1—S2—P2—C4	−162.7 (2)
S2—S1—P1—C1	162.1 (2)		

**Table 2:** Selected geometric parameters (Å, °) for **2**[Chem scheme1]

Au1—S1	2.2928 (6)	P2—S2	2.0444 (8)
Au1—S2	2.2990 (7)	I1—I2	2.9238 (11)
P1—S1	2.0350 (8)	I2—I3	2.9213 (11)
			
S1—Au1—S2	177.53 (2)	P2—S2—Au1	104.09 (3)
P1—S1—Au1	104.94 (3)	I3—I2—I1	178.44 (5)
			
C3—P1—S1—Au1	−51.13 (9)	C6—P2—S2—Au1	36.45 (9)
C1—P1—S1—Au1	−171.42 (8)	C4—P2—S2—Au1	157.30 (9)
C2—P1—S1—Au1	67.11 (9)	C5—P2—S2—Au1	−80.90 (9)

**Table 3:** Selected geometric parameters (Å, °) for **3**[Chem scheme1]

Au1—S1	2.2920 (9)	S2—P2	2.0308 (13)
Au1—S2	2.2924 (9)	I1—I2	2.8755 (7)
S1—P1	2.0251 (13)	I2—I3	2.9347 (17)
			
S1—Au1—S2	177.07 (4)	P2—S2—Au1	104.82 (5)
P1—S1—Au1	101.43 (4)	I1—I2—I3	178.46 (4)
			
Au1—S1—P1—C2	−51.38 (13)	Au1—S2—P2—C5	42.46 (13)
Au1—S1—P1—C3	66.44 (13)	Au1—S2—P2—C6	−73.81 (13)
Au1—S1—P1—C1	−175.01 (13)	Au1—S2—P2—C4	166.94 (12)

**Table 4:** Selected geometric parameters (Å, °) for **4**[Chem scheme1]

Au1—S1	2.2818 (6)	I2—I3	3.2674 (2)
P1—S1	2.0441 (9)	I3—I4	2.7501 (3)
I1—I2	2.9204 (2)		
			
S1—Au1—S1^i^	176.40 (3)	I1—I2—I3	112.048 (7)
P1—S1—Au1	107.14 (3)	I4—I3—I2	173.271 (9)
I2^ii^—I1—I2	177.495 (11)		
			
C3—P1—S1—Au1	−58.76 (10)	C1—P1—S1—Au1	59.64 (9)
C2—P1—S1—Au1	−178.28 (9)		

Symmetry codes: (i) 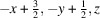; (ii) 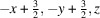.

**Table 5:** Selected geometric parameters (Å, °) for **5**[Chem scheme1]

Au1—S1	2.3336 (11)	P1—S1	2.0594 (15)
Au1—Br1	2.4357 (5)		
			
S1—Au1—S1^i^	84.71 (5)	Br1^i^—Au1—Br1	93.53 (2)
S1—Au1—Br1^i^	175.54 (3)	S1^i^—P1—S1	99.53 (9)
S1—Au1—Br1	90.88 (3)	P1—S1—Au1	87.68 (5)
			
C1—P1—S1—Au1	−120.49 (19)	S1^i^—Au1—S1—P1	4.50 (7)
C2—P1—S1—Au1	108.93 (17)	Br1—Au1—S1—P1	−176.12 (6)
S1^i^—P1—S1—Au1	−5.15 (8)		

Symmetry code: (i) 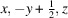.

**Table 6:** Selected geometric parameters (Å, °) for **6**[Chem scheme1]

Au1—Br4	2.4185 (6)	O2—P2	1.528 (3)
Au1—Br1	2.4235 (5)	P1—Se1	2.2743 (12)
Au1—Br2	2.4291 (6)	P2—Se2	2.2653 (13)
Au1—Br3	2.4301 (5)	Se1—Se2	2.3314 (6)
O1—P1	1.528 (3)		
			
Br4—Au1—Br1	89.397 (19)	Br2—Au1—Br3	90.99 (2)
Br4—Au1—Br2	177.20 (2)	O1—P1—Se1	111.02 (13)
Br1—Au1—Br2	89.539 (19)	O2—P2—Se2	111.23 (13)
Br4—Au1—Br3	90.15 (2)	P1—Se1—Se2	100.44 (4)
Br1—Au1—Br3	178.07 (2)	P2—Se2—Se1	102.12 (4)
			
O1—P1—Se1—Se2	32.74 (14)	C4—P2—Se2—Se1	−78.79 (17)
C1—P1—Se1—Se2	−85.24 (17)	C3—P2—Se2—Se1	158.03 (15)
C2—P1—Se1—Se2	150.45 (16)	P1—Se1—Se2—P2	−71.86 (5)
O2—P2—Se2—Se1	39.99 (14)		

**Table 7:** Hydrogen-bond geometry (Å, °) for **1**[Chem scheme1]

*D*—H⋯*A*	*D*—H	H⋯*A*	*D*⋯*A*	*D*—H⋯*A*
C5—H5⋯S1	1.00	2.85	3.358 (6)	112
C13—H13*B*⋯S1	0.98	2.66	3.099 (6)	108
C21—H21*B*⋯S1	0.98	2.85	3.468 (6)	122
C62—H62*A*⋯S1	0.98	2.86	3.535 (7)	127
C2—H2⋯S2	1.00	2.80	3.273 (6)	110
C43—H43*B*⋯S2	0.98	2.57	3.105 (6)	114
C41—H41*A*⋯Cl1^i^	0.98	2.95	3.879 (6)	159
C5—H5⋯Cl2	1.00	2.72	3.521 (6)	138
C51—H51*A*⋯Cl2	0.98	2.89	3.567 (6)	127
C3—H3⋯Cl3^ii^	1.00	2.75	3.640 (6)	149
C11—H11*B*⋯Cl4^ii^	0.98	2.85	3.619 (6)	136
C2—H2⋯Cl5	1.00	2.58	3.439 (6)	143
C6—H6⋯Cl8^iii^	1.00	2.73	3.600 (6)	146
C6—H6⋯Au2^iii^	1.00	3.25	3.851 (6)	121

Symmetry codes: (i) 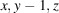; (ii) 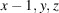; (iii) 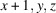.

**Table 8:** Hydrogen-bond geometry (Å, °) for **2**)

*D*—H⋯*A*	*D*—H	H⋯*A*	*D*⋯*A*	*D*—H⋯*A*
C23—H23*B*⋯Au1	0.98	2.72	3.616 (3)	152
C53—H53*B*⋯Au1	0.98	2.90	3.747 (3)	145
C22—H22*C*⋯S1	0.98	2.82	3.299 (3)	111
C11—H11*C*⋯S1	0.98	2.68	3.194 (3)	113
C52—H52*B*⋯S2	0.98	2.77	3.292 (3)	114
C41—H41*B*⋯S2	0.98	2.70	3.214 (3)	113
C3—H3⋯I3	1.00	3.29	4.202 (3)	152
C6—H6⋯I1^i^	1.00	3.21	3.993 (3)	136
C51—H51*B*⋯I2^ii^	0.98	3.25	4.055 (3)	140
C61—H61*A*⋯I2^iii^	0.98	3.26	4.003 (3)	134

Symmetry codes: (i) 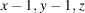; (ii) 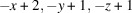; (iii) 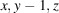.

**Table 9:** Hydrogen-bond geometry (Å, °) for **3**[Chem scheme1]

*D*—H⋯*A*	*D*—H	H⋯*A*	*D*⋯*A*	*D*—H⋯*A*
C5—H5⋯I1^i^	1.00	3.01	3.877 (4)	146
C11—H11*B*⋯I1^ii^	0.98	3.21	4.160 (4)	163
C43—H43*C*⋯I1^iii^	0.98	3.16	4.130 (4)	169
C3—H3⋯I2^iv^	1.00	3.21	3.979 (4)	135
C2—H2⋯I3	1.00	3.04	3.878 (4)	142
C13—H13*A*⋯I3^v^	0.98	3.19	4.154 (4)	170
C61—H61*C*⋯I3^vi^	0.98	3.19	4.123 (4)	159
C32—H32*A*⋯Au1	0.98	2.77	3.520 (4)	134
C62—H62*C*⋯Au1	0.98	2.88	3.675 (5)	139

Symmetry codes: (i) 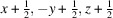; (ii) 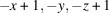; (iii) 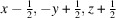; (iv) 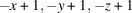; (v) 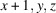; (vi) 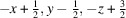.

**Table 10:** Hydrogen-bond geometry (Å, °) for **4**[Chem scheme1]

*D*—H⋯*A*	*D*—H	H⋯*A*	*D*⋯*A*	*D*—H⋯*A*
C13—H13*A*⋯Au1	0.98	2.70	3.594 (3)	152
C21—H21*C*⋯S1	0.98	2.64	3.170 (3)	114
C31—H31*B*⋯I3^iii^	0.98	3.25	4.102 (3)	146
C3—H3⋯I3^iv^	1.00	3.18	4.059 (3)	148
C21—H21*A*⋯I3^v^	0.98	3.28	4.119 (3)	145
C22—H22*C*⋯I3^v^	0.98	3.18	3.962 (3)	138

Symmetry codes: (iii) 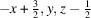; (iv) 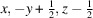; (v) 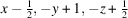.

**Table 11:** Hydrogen-bond geometry (Å, °) for **5**[Chem scheme1]

*D*—H⋯*A*	*D*—H	H⋯*A*	*D*⋯*A*	*D*—H⋯*A*
C11—H11*B*⋯S1	0.96	2.90	3.417 (7)	115
C12—H12*B*⋯S1	0.98	2.99	3.573 (5)	119
C12—H12*C*⋯Br1^ii^	0.98	3.13	3.995 (6)	147

Symmetry code: (ii) 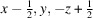.

**Table 12:** Hydrogen-bond geometry (Å, °) for **6**[Chem scheme1]

*D*—H⋯*A*	*D*—H	H⋯*A*	*D*⋯*A*	*D*—H⋯*A*
C42—H42*A*⋯Au1^i^	0.98	2.85	3.666 (5)	141
C21—H21*C*⋯Br1^ii^	0.98	3.02	3.899 (5)	149
C22—H22*B*⋯Br2^iii^	0.98	3.04	3.717 (5)	128
C33—H33*B*⋯Br1	0.98	3.07	3.956 (5)	151
C42—H42*B*⋯Br4^iv^	0.98	2.89	3.532 (5)	124
O1—H01⋯O2	0.95 (8)	1.51 (8)	2.450 (4)	169 (8)

Symmetry codes: (i) 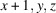; (ii) 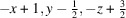; (iii) 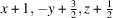; (iv) 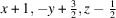.

**Table:** 

	**1**	**2**	**3**
Crystal data			
Chemical formula	(C_20_H_46_P_2_S_2_)[AuCl_4_]_2_	[Au(C_11_H_25_PS)_2_][AuI_2_]_0.095_(I_3_)_0.905_	[Au(C_10_H_23_PS)_2_][AuI_2_]_0.125_(I_3_)_0.875_
*M*_r_	1090.16	1025.00	999.05
Crystal system, space group	Triclinic, *P* 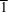	Triclinic, *P* 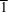	Monoclinic, *P*2_1_/*n*
Temperature (K)	100	100	100
*a*, *b*, *c* (Å)	10.0655 (5), 13.8440 (6), 14.1212 (6)	8.7116 (3), 12.2732 (4), 16.2730 (4)	11.6180 (2), 11.8894 (3), 22.5768 (5)
α, β, γ (°)	73.579 (4), 80.850 (4), 71.620 (4)	110.531 (3), 90.770 (3), 94.514 (3)	90, 90.090 (2), 90
*V* (Å^3^)	1785.90 (15)	1622.76 (9)	3118.54 (12)
*Z*	2	2	4
Radiation type	Mo *K*α	Mo *K*α	Mo *K*α
μ (mm^−1^)	9.02	7.96	8.39
Crystal size (mm)	0.2 × 0.03 × 0.01	0.4 × 0.2 × 0.04	0.15 × 0.08 × 0.05

Data collection			
Diffractometer	Oxford Diffraction Xcalibur, Eos	Oxford Diffraction Xcalibur, Eos	Oxford Diffraction Xcalibur, Eos
Absorption correction	Multi-scan (*CrysAlis PRO*; Rigaku OD, 2015 ▸)	Multi-scan (*CrysAlis PRO*; Rigaku OD, 2015 ▸)	Multi-scan (*CrysAlis PRO*; Rigaku OD, 2015 ▸)
*T*_min_, *T*_max_	0.764, 1.000	0.150, 0.750	0.565, 1.000
No. of measured, independent and observed [*I* > 2σ(*I*)] reflections	68998, 9104, 6753	126632, 9377, 8340	142434, 7725, 7218
*R*_int_	0.079	0.045	0.072
θ values (°)	θ_max_ = 29.4, θ_min_ = 2.3	θ_max_ = 30.0, θ_min_ = 2.4	θ_max_ = 28.3, θ_min_ = 2.5
(sin θ/λ)_max_ (Å^−1^)	0.690	0.704	0.667
Range of *h*, *k*, *l*	*h* = −13→13, *k* = −18→18, *l* = −18→19	*h* = −12→12, *k* = −17→17, *l* = −22→22	*h* = −15→15, *k* = −15→15, *l* = −30→30

Refinement			
*R*[*F*^2^ > 2σ(*F*^2^)], *wR*(*F*^2^), *S*	0.042, 0.079, 1.03	0.023, 0.042, 1.05	0.022, 0.041, 1.08
No. of reflections	9104	9377	7725
No. of parameters	321	298	281
No. of restraints	0	2	7
H-atom treatment	H-atom parameters constrained	H-atom parameters constrained	H-atom parameters constrained
Δρ_max_, Δρ_min_ (e Å^−3^)	1.90, −2.15	1.32, −0.86	0.65, −0.79

**Table:** 

	**4**	**5**	**6**
Crystal data			
Chemical formula	[Au(C_11_H_25_PS)_2_]I_3_·2I_2_	[AuBr_2_(C_8_H_18_PS_2_)]	(C_16_H_37_O_2_P_2_Se_2_)[AuBr_4_]
*M*_r_	1525.94	566.10	997.92
Crystal system, space group	Orthorhombic, *Pccn*	Orthorhombic, *Pnma*	Monoclinic, *P*2_1_/*c*
Temperature (K)	100	100	100
*a*, *b*, *c* (Å)	15.0435 (3), 15.2629 (3), 17.7574 (3)	16.0199 (6), 11.5880 (4), 8.0211 (3)	8.4102 (2), 22.2796 (7), 15.4285 (5)
α, β, γ (°)	90, 90, 90	90, 90, 90	90, 99.221 (3), 90
*V* (Å^3^)	4077.24 (13)	1489.03 (9)	2853.57 (16)
*Z*	4	4	4
Radiation type	Mo *K*α	Mo *K*α	Mo *K*α
μ (mm^−1^)	9.10	15.60	13.43
Crystal size (mm)	0.2 × 0.08 × 0.03	0.13 × 0.05 × 0.04	0.2 × 0.02 × 0.02

Data collection			
Diffractometer	Oxford Diffraction Xcalibur, Eos	Oxford Diffraction Xcalibur, Eos	Oxford Diffraction Xcalibur, Eos
Absorption correction	Multi-scan (*CrysAlis PRO*; Rigaku OD, 2015 ▸)	Multi-scan (*CrysAlis PRO*; Rigaku OD, 2015 ▸)	Multi-scan (*CrysAlis PRO*; Rigaku OD, 2015 ▸)
*T*_min_, *T*_max_	0.263, 0.772	0.635, 1.000	0.514, 1.000
No. of measured, independent and observed [*I* > 2σ(*I*)] reflections	112897, 5958, 5144	35299, 2061, 1712	133232, 7081, 5668
*R*_int_	0.053	0.076	0.116
θ values (°)	θ_max_ = 30.0, θ_min_ = 2.2	θ_max_ = 29.3, θ_min_ = 2.5	θ_max_ = 28.3, θ_min_ = 2.3
(sin θ/λ)_max_ (Å^−1^)	0.704	0.689	0.667
Range of *h*, *k*, *l*	*h* = −20→21, *k* = −21→21, *l* = −25→25	*h* = −21→21, *k* = −15→15, *l* = −10→11	*h* = −11→11, *k* = −29→29, *l* = −20→20

Refinement			
*R*[*F*^2^ > 2σ(*F*^2^)], *wR*(*F*^2^), *S*	0.021, 0.034, 1.11	0.029, 0.056, 1.06	0.036, 0.056, 1.05
No. of reflections	5958	2061	7081
No. of parameters	164	75	260
No. of restraints	0	0	0
H-atom treatment	H-atom parameters constrained	H-atom parameters constrained	H atoms treated by a mixture of independent and constrained refinement
Δρ_max_, Δρ_min_ (e Å^−3^)	0.95, −1.39	1.88, −1.03	0.98, −1.23

Computer programs: *CrysAlis PRO* (Rigaku OD, 2015 ▸), *SHELXS97* (Sheldrick, 2008 ▸), *SHELXL2019/3* (Sheldrick, 2015 ▸) and *XP* (Bruker, 1998 ▸).

## supplementary crystallographic information

### (Disulfane-1,2-diyl)bis(tert-butyldiisopropylphosphonium) bis[tetrachloridoaurate(III)] (1) Crystal data

**Table:**

(C_20_H_46_P_2_S_2_)[AuCl_4_]_2_	*Z* = 2
*M**_r_* = 1090.16	*F*(000) = 1044
Triclinic, *P*1	*D*_x_ = 2.027 Mg m^−^^3^
*a* = 10.0655 (5) Å	Mo *K*α radiation, λ = 0.71073 Å
*b* = 13.8440 (6) Å	Cell parameters from 9063 reflections
*c* = 14.1212 (6) Å	θ = 2.3–29.3°
α = 73.579 (4)°	µ = 9.02 mm^−^^1^
β = 80.850 (4)°	*T* = 100 K
γ = 71.620 (4)°	Needle, yellow
*V* = 1785.90 (15) Å^3^	0.2 × 0.03 × 0.01 mm

### (Disulfane-1,2-diyl)bis(tert-butyldiisopropylphosphonium) bis[tetrachloridoaurate(III)] (1) Data collection

**Table:**

Oxford Diffraction Xcalibur, Eos diffractometer	9104 independent reflections
Radiation source: fine-focus sealed tube	6753 reflections with *I* > 2σ(*I*)
Detector resolution: 16.1419 pixels mm^-1^	*R*_int_ = 0.079
ω scan	θ_max_ = 29.4°, θ_min_ = 2.3°
Absorption correction: multi-scan (CrysAlisPro; Rigaku OD, 2015)	*h* = −13→13
*T*_min_ = 0.764, *T*_max_ = 1.000	*k* = −18→18
68998 measured reflections	*l* = −18→19

### (Disulfane-1,2-diyl)bis(tert-butyldiisopropylphosphonium) bis[tetrachloridoaurate(III)] (1) Refinement

**Table:**

Refinement on *F*^2^	Primary atom site location: structure-invariant direct methods
Least-squares matrix: full	Secondary atom site location: difference Fourier map
*R*[*F*^2^ > 2σ(*F*^2^)] = 0.042	Hydrogen site location: inferred from neighbouring sites
*wR*(*F*^2^) = 0.079	H-atom parameters constrained
*S* = 1.02	*w* = 1/[σ^2^(*F*_o_^2^) + (0.0245*P*)^2^ + 3.847*P*] where *P* = (*F*_o_^2^ + 2*F*_c_^2^)/3
9104 reflections	(Δ/σ)_max_ = 0.001
321 parameters	Δρ_max_ = 1.90 e Å^−^^3^
0 restraints	Δρ_min_ = −2.15 e Å^−^^3^

### (Disulfane-1,2-diyl)bis(tert-butyldiisopropylphosphonium) bis[tetrachloridoaurate(III)] (1) Fractional atomic coordinates and isotropic or equivalent isotropic displacement parameters (Å^2^)

**Table:**

	*x*	*y*	*z*	*U*_iso_*/*U*_eq_	
S1	0.52654 (16)	0.31308 (12)	0.28469 (11)	0.0241 (3)	
S2	0.53274 (16)	0.16309 (12)	0.27077 (11)	0.0246 (3)	
P1	0.31753 (15)	0.36141 (11)	0.34119 (10)	0.0157 (3)	
P2	0.71921 (16)	0.12892 (12)	0.18029 (10)	0.0170 (3)	
C1	0.3194 (6)	0.4692 (5)	0.3937 (4)	0.0199 (13)	
C2	0.2769 (6)	0.2472 (4)	0.4287 (4)	0.0188 (12)	
H2	0.278979	0.198315	0.387868	0.023*	
C3	0.1945 (6)	0.4079 (5)	0.2444 (4)	0.0217 (13)	
H3	0.099500	0.437578	0.275834	0.026*	
C4	0.7020 (6)	0.0215 (4)	0.1330 (4)	0.0200 (13)	
C5	0.7333 (6)	0.2485 (4)	0.0875 (4)	0.0197 (13)	
H5	0.739470	0.297157	0.126079	0.024*	
C6	0.8691 (6)	0.0847 (5)	0.2561 (4)	0.0213 (13)	
H6	0.955132	0.060900	0.212645	0.026*	
C11	0.1682 (6)	0.5395 (5)	0.4037 (5)	0.0252 (14)	
H11A	0.107621	0.496379	0.441581	0.038*	
H11B	0.134583	0.574742	0.337720	0.038*	
H11C	0.166032	0.592343	0.438229	0.038*	
C12	0.3757 (7)	0.4226 (5)	0.4968 (4)	0.0271 (14)	
H12A	0.383501	0.479167	0.522618	0.041*	
H12B	0.468486	0.372100	0.491988	0.041*	
H12C	0.311227	0.386962	0.541526	0.041*	
C13	0.4131 (6)	0.5340 (5)	0.3264 (4)	0.0242 (13)	
H13A	0.385462	0.555483	0.258332	0.036*	
H13B	0.511446	0.491217	0.328121	0.036*	
H13C	0.402213	0.596515	0.349732	0.036*	
C21	0.3845 (6)	0.1840 (5)	0.5058 (4)	0.0242 (14)	
H21A	0.364132	0.216249	0.561984	0.036*	
H21B	0.479175	0.183950	0.475612	0.036*	
H21C	0.378807	0.111612	0.529033	0.036*	
C22	0.1254 (6)	0.2753 (5)	0.4771 (4)	0.0258 (14)	
H22A	0.097471	0.210843	0.506794	0.039*	
H22B	0.061462	0.321518	0.426583	0.039*	
H22C	0.121208	0.311334	0.528528	0.039*	
C31	0.2263 (7)	0.4963 (5)	0.1599 (4)	0.0344 (17)	
H31A	0.322347	0.472422	0.130903	0.052*	
H31B	0.216988	0.557257	0.185356	0.052*	
H31C	0.160059	0.516174	0.109056	0.052*	
C32	0.1841 (7)	0.3191 (5)	0.2041 (4)	0.0293 (15)	
H32A	0.109627	0.346501	0.158282	0.044*	
H32B	0.162241	0.263387	0.259114	0.044*	
H32C	0.273820	0.290449	0.169043	0.044*	
C41	0.8494 (6)	−0.0407 (5)	0.0999 (4)	0.0240 (14)	
H41A	0.841976	−0.096248	0.072497	0.036*	
H41B	0.906553	−0.072244	0.156927	0.036*	
H41C	0.893673	0.006768	0.049167	0.036*	
C42	0.6109 (7)	0.0694 (5)	0.0442 (4)	0.0276 (14)	
H42A	0.659777	0.109850	−0.010536	0.041*	
H42B	0.521102	0.115972	0.063547	0.041*	
H42C	0.593806	0.013109	0.023030	0.041*	
C43	0.6303 (7)	−0.0508 (5)	0.2137 (4)	0.0286 (15)	
H43A	0.621480	−0.106747	0.187581	0.043*	
H43B	0.536767	−0.009655	0.234000	0.043*	
H43C	0.686962	−0.081872	0.271025	0.043*	
C51	0.6051 (6)	0.3082 (5)	0.0270 (4)	0.0244 (14)	
H51A	0.603333	0.382206	0.001495	0.037*	
H51B	0.519144	0.303369	0.069343	0.037*	
H51C	0.611019	0.277330	−0.028551	0.037*	
C52	0.8728 (6)	0.2284 (5)	0.0234 (4)	0.0235 (14)	
H52A	0.870440	0.185975	−0.021044	0.035*	
H52B	0.950306	0.190741	0.066156	0.035*	
H52C	0.886766	0.295665	−0.015949	0.035*	
C61	0.8625 (7)	−0.0097 (5)	0.3439 (4)	0.0315 (16)	
H61A	0.938022	−0.024430	0.386509	0.047*	
H61B	0.873578	−0.071406	0.319132	0.047*	
H61C	0.771539	0.006479	0.382090	0.047*	
C62	0.8875 (7)	0.1747 (5)	0.2917 (5)	0.0288 (15)	
H62A	0.806779	0.198239	0.336963	0.043*	
H62B	0.893801	0.233350	0.234613	0.043*	
H62C	0.973668	0.149661	0.326320	0.043*	
Au1	0.77846 (2)	0.59195 (2)	0.17918 (2)	0.02045 (7)	
Cl1	0.73799 (19)	0.73588 (13)	0.04884 (11)	0.0345 (4)	
Cl2	0.68846 (19)	0.50729 (13)	0.10043 (11)	0.0350 (4)	
Cl3	0.81987 (16)	0.44619 (12)	0.30933 (11)	0.0279 (3)	
Cl4	0.8680 (2)	0.67693 (13)	0.25853 (13)	0.0388 (4)	
Au2	0.24397 (2)	−0.09207 (2)	0.30906 (2)	0.01939 (7)	
Cl5	0.28990 (18)	0.00673 (12)	0.39762 (11)	0.0289 (4)	
Cl6	0.25095 (17)	−0.22473 (12)	0.44956 (10)	0.0269 (3)	
Cl7	0.1957 (2)	−0.18989 (13)	0.22072 (12)	0.0372 (4)	
Cl8	0.23425 (17)	0.04326 (12)	0.16984 (11)	0.0280 (3)	

### (Disulfane-1,2-diyl)bis(tert-butyldiisopropylphosphonium) bis[tetrachloridoaurate(III)] (1) Atomic displacement parameters (Å^2^)

**Table:**

	*U*^11^	*U*^22^	*U*^33^	*U*^12^	*U*^13^	*U*^23^
S1	0.0243 (8)	0.0186 (8)	0.0309 (8)	−0.0099 (6)	0.0073 (7)	−0.0094 (6)
S2	0.0273 (9)	0.0181 (8)	0.0295 (8)	−0.0103 (7)	0.0107 (7)	−0.0103 (6)
P1	0.0201 (8)	0.0146 (7)	0.0131 (7)	−0.0080 (6)	−0.0004 (6)	−0.0016 (6)
P2	0.0212 (8)	0.0149 (7)	0.0153 (7)	−0.0085 (6)	0.0026 (6)	−0.0030 (6)
C1	0.019 (3)	0.019 (3)	0.024 (3)	−0.007 (3)	−0.003 (2)	−0.007 (2)
C2	0.024 (3)	0.015 (3)	0.016 (3)	−0.008 (2)	−0.003 (2)	0.003 (2)
C3	0.030 (3)	0.023 (3)	0.015 (3)	−0.010 (3)	−0.004 (2)	−0.004 (2)
C4	0.024 (3)	0.017 (3)	0.021 (3)	−0.009 (3)	−0.001 (2)	−0.006 (2)
C5	0.023 (3)	0.017 (3)	0.019 (3)	−0.009 (3)	−0.001 (2)	−0.001 (2)
C6	0.024 (3)	0.023 (3)	0.017 (3)	−0.013 (3)	0.000 (2)	0.000 (2)
C11	0.021 (3)	0.022 (3)	0.031 (3)	−0.003 (3)	−0.001 (3)	−0.008 (3)
C12	0.031 (4)	0.029 (4)	0.024 (3)	−0.011 (3)	−0.002 (3)	−0.009 (3)
C13	0.027 (3)	0.020 (3)	0.027 (3)	−0.010 (3)	0.001 (3)	−0.006 (3)
C21	0.032 (4)	0.017 (3)	0.022 (3)	−0.010 (3)	−0.002 (3)	0.000 (2)
C22	0.028 (4)	0.028 (4)	0.020 (3)	−0.011 (3)	0.001 (3)	0.000 (3)
C31	0.050 (5)	0.032 (4)	0.016 (3)	−0.014 (3)	−0.011 (3)	0.010 (3)
C32	0.038 (4)	0.033 (4)	0.019 (3)	−0.016 (3)	−0.010 (3)	0.001 (3)
C41	0.029 (3)	0.019 (3)	0.027 (3)	−0.008 (3)	0.007 (3)	−0.012 (3)
C42	0.033 (4)	0.026 (4)	0.032 (3)	−0.015 (3)	−0.003 (3)	−0.015 (3)
C43	0.040 (4)	0.023 (3)	0.029 (3)	−0.019 (3)	0.009 (3)	−0.011 (3)
C51	0.033 (4)	0.014 (3)	0.024 (3)	−0.005 (3)	−0.004 (3)	−0.003 (2)
C52	0.026 (3)	0.024 (3)	0.019 (3)	−0.011 (3)	0.000 (3)	0.003 (2)
C61	0.035 (4)	0.040 (4)	0.019 (3)	−0.014 (3)	−0.005 (3)	0.002 (3)
C62	0.028 (4)	0.036 (4)	0.029 (3)	−0.013 (3)	0.000 (3)	−0.014 (3)
Au1	0.02575 (14)	0.01772 (13)	0.01936 (12)	−0.00711 (10)	−0.00538 (10)	−0.00388 (9)
Cl1	0.0566 (11)	0.0248 (9)	0.0261 (8)	−0.0202 (8)	−0.0147 (8)	0.0034 (7)
Cl2	0.0605 (12)	0.0284 (9)	0.0250 (8)	−0.0225 (8)	−0.0144 (8)	−0.0035 (7)
Cl3	0.0315 (9)	0.0219 (8)	0.0266 (8)	−0.0068 (7)	−0.0087 (7)	0.0023 (6)
Cl4	0.0550 (12)	0.0266 (9)	0.0423 (10)	−0.0133 (8)	−0.0273 (9)	−0.0055 (7)
Au2	0.02334 (13)	0.01737 (13)	0.01839 (12)	−0.00646 (10)	−0.00476 (9)	−0.00345 (9)
Cl5	0.0458 (10)	0.0230 (8)	0.0238 (7)	−0.0160 (7)	−0.0049 (7)	−0.0066 (6)
Cl6	0.0402 (9)	0.0211 (8)	0.0209 (7)	−0.0128 (7)	−0.0073 (7)	−0.0005 (6)
Cl7	0.0600 (12)	0.0290 (9)	0.0308 (8)	−0.0166 (8)	−0.0192 (8)	−0.0074 (7)
Cl8	0.0329 (9)	0.0273 (8)	0.0208 (7)	−0.0105 (7)	−0.0044 (6)	0.0019 (6)

### (Disulfane-1,2-diyl)bis(tert-butyldiisopropylphosphonium) bis[tetrachloridoaurate(III)] (1) Geometric parameters (Å, º)

**Table:**

S1—P1	2.095 (2)	C22—H22A	0.9800
S1—S2	2.121 (2)	C22—H22B	0.9800
S2—P2	2.097 (2)	C22—H22C	0.9800
P1—C2	1.826 (5)	C31—H31A	0.9800
P1—C3	1.832 (6)	C31—H31B	0.9800
P1—C1	1.851 (6)	C31—H31C	0.9800
P2—C5	1.827 (5)	C32—H32A	0.9800
P2—C6	1.832 (6)	C32—H32B	0.9800
P2—C4	1.858 (6)	C32—H32C	0.9800
C1—C12	1.535 (8)	C41—H41A	0.9800
C1—C11	1.536 (8)	C41—H41B	0.9800
C1—C13	1.538 (7)	C41—H41C	0.9800
C2—C21	1.537 (8)	C42—H42A	0.9800
C2—C22	1.545 (8)	C42—H42B	0.9800
C2—H2	1.0000	C42—H42C	0.9800
C3—C31	1.525 (7)	C43—H43A	0.9800
C3—C32	1.530 (8)	C43—H43B	0.9800
C3—H3	1.0000	C43—H43C	0.9800
C4—C41	1.537 (8)	C51—H51A	0.9800
C4—C42	1.537 (8)	C51—H51B	0.9800
C4—C43	1.540 (7)	C51—H51C	0.9800
C5—C51	1.535 (8)	C52—H52A	0.9800
C5—C52	1.537 (8)	C52—H52B	0.9800
C5—H5	1.0000	C52—H52C	0.9800
C6—C61	1.538 (7)	C61—H61A	0.9800
C6—C62	1.539 (8)	C61—H61B	0.9800
C6—H6	1.0000	C61—H61C	0.9800
C11—H11A	0.9800	C62—H62A	0.9800
C11—H11B	0.9800	C62—H62B	0.9800
C11—H11C	0.9800	C62—H62C	0.9800
C12—H12A	0.9800	Au1—Cl2	2.2711 (16)
C12—H12B	0.9800	Au1—Cl1	2.2753 (15)
C12—H12C	0.9800	Au1—Cl4	2.2772 (16)
C13—H13A	0.9800	Au1—Cl3	2.2891 (14)
C13—H13B	0.9800	Au2—Cl7	2.2779 (16)
C13—H13C	0.9800	Au2—Cl5	2.2800 (15)
C21—H21A	0.9800	Au2—Cl6	2.2841 (14)
C21—H21B	0.9800	Au2—Cl8	2.2908 (14)
C21—H21C	0.9800		
			
P1—S1—S2	99.30 (8)	H21B—C21—H21C	109.5
P2—S2—S1	100.59 (8)	C2—C22—H22A	109.5
C2—P1—C3	107.1 (3)	C2—C22—H22B	109.5
C2—P1—C1	116.4 (3)	H22A—C22—H22B	109.5
C3—P1—C1	111.6 (3)	C2—C22—H22C	109.5
C2—P1—S1	107.7 (2)	H22A—C22—H22C	109.5
C3—P1—S1	112.1 (2)	H22B—C22—H22C	109.5
C1—P1—S1	101.84 (19)	C3—C31—H31A	109.5
C5—P2—C6	107.5 (3)	C3—C31—H31B	109.5
C5—P2—C4	116.4 (3)	H31A—C31—H31B	109.5
C6—P2—C4	111.8 (3)	C3—C31—H31C	109.5
C5—P2—S2	109.4 (2)	H31A—C31—H31C	109.5
C6—P2—S2	109.10 (19)	H31B—C31—H31C	109.5
C4—P2—S2	102.46 (19)	C3—C32—H32A	109.5
C12—C1—C11	109.0 (5)	C3—C32—H32B	109.5
C12—C1—C13	109.4 (5)	H32A—C32—H32B	109.5
C11—C1—C13	110.1 (5)	C3—C32—H32C	109.5
C12—C1—P1	109.4 (4)	H32A—C32—H32C	109.5
C11—C1—P1	108.5 (4)	H32B—C32—H32C	109.5
C13—C1—P1	110.5 (4)	C4—C41—H41A	109.5
C21—C2—C22	112.0 (4)	C4—C41—H41B	109.5
C21—C2—P1	115.5 (4)	H41A—C41—H41B	109.5
C22—C2—P1	111.8 (4)	C4—C41—H41C	109.5
C21—C2—H2	105.5	H41A—C41—H41C	109.5
C22—C2—H2	105.5	H41B—C41—H41C	109.5
P1—C2—H2	105.5	C4—C42—H42A	109.5
C31—C3—C32	110.4 (5)	C4—C42—H42B	109.5
C31—C3—P1	112.8 (4)	H42A—C42—H42B	109.5
C32—C3—P1	112.7 (4)	C4—C42—H42C	109.5
C31—C3—H3	106.8	H42A—C42—H42C	109.5
C32—C3—H3	106.8	H42B—C42—H42C	109.5
P1—C3—H3	106.8	C4—C43—H43A	109.5
C41—C4—C42	109.7 (5)	C4—C43—H43B	109.5
C41—C4—C43	110.7 (5)	H43A—C43—H43B	109.5
C42—C4—C43	108.4 (5)	C4—C43—H43C	109.5
C41—C4—P2	108.4 (4)	H43A—C43—H43C	109.5
C42—C4—P2	109.1 (4)	H43B—C43—H43C	109.5
C43—C4—P2	110.4 (4)	C5—C51—H51A	109.5
C51—C5—C52	113.4 (5)	C5—C51—H51B	109.5
C51—C5—P2	115.7 (4)	H51A—C51—H51B	109.5
C52—C5—P2	111.1 (4)	C5—C51—H51C	109.5
C51—C5—H5	105.2	H51A—C51—H51C	109.5
C52—C5—H5	105.2	H51B—C51—H51C	109.5
P2—C5—H5	105.2	C5—C52—H52A	109.5
C61—C6—C62	110.7 (5)	C5—C52—H52B	109.5
C61—C6—P2	112.8 (4)	H52A—C52—H52B	109.5
C62—C6—P2	112.0 (4)	C5—C52—H52C	109.5
C61—C6—H6	107.0	H52A—C52—H52C	109.5
C62—C6—H6	107.0	H52B—C52—H52C	109.5
P2—C6—H6	107.0	C6—C61—H61A	109.5
C1—C11—H11A	109.5	C6—C61—H61B	109.5
C1—C11—H11B	109.5	H61A—C61—H61B	109.5
H11A—C11—H11B	109.5	C6—C61—H61C	109.5
C1—C11—H11C	109.5	H61A—C61—H61C	109.5
H11A—C11—H11C	109.5	H61B—C61—H61C	109.5
H11B—C11—H11C	109.5	C6—C62—H62A	109.5
C1—C12—H12A	109.5	C6—C62—H62B	109.5
C1—C12—H12B	109.5	H62A—C62—H62B	109.5
H12A—C12—H12B	109.5	C6—C62—H62C	109.5
C1—C12—H12C	109.5	H62A—C62—H62C	109.5
H12A—C12—H12C	109.5	H62B—C62—H62C	109.5
H12B—C12—H12C	109.5	Cl2—Au1—Cl1	90.20 (6)
C1—C13—H13A	109.5	Cl2—Au1—Cl4	179.79 (7)
C1—C13—H13B	109.5	Cl1—Au1—Cl4	89.86 (6)
H13A—C13—H13B	109.5	Cl2—Au1—Cl3	89.42 (6)
C1—C13—H13C	109.5	Cl1—Au1—Cl3	179.48 (6)
H13A—C13—H13C	109.5	Cl4—Au1—Cl3	90.53 (6)
H13B—C13—H13C	109.5	Cl7—Au2—Cl5	179.42 (6)
C2—C21—H21A	109.5	Cl7—Au2—Cl6	90.55 (6)
C2—C21—H21B	109.5	Cl5—Au2—Cl6	89.54 (5)
H21A—C21—H21B	109.5	Cl7—Au2—Cl8	90.21 (6)
C2—C21—H21C	109.5	Cl5—Au2—Cl8	89.69 (6)
H21A—C21—H21C	109.5	Cl6—Au2—Cl8	178.88 (6)
			
P1—S1—S2—P2	165.51 (8)	C2—P1—C3—C32	−47.9 (5)
S2—S1—P1—C2	39.1 (2)	C1—P1—C3—C32	−176.5 (4)
S2—S1—P1—C3	−78.5 (2)	S1—P1—C3—C32	70.0 (4)
S2—S1—P1—C1	162.1 (2)	C5—P2—C4—C41	82.6 (4)
S1—S2—P2—C5	−38.6 (2)	C6—P2—C4—C41	−41.4 (4)
S1—S2—P2—C6	78.7 (2)	S2—P2—C4—C41	−158.1 (3)
S1—S2—P2—C4	−162.7 (2)	C5—P2—C4—C42	−36.8 (5)
C2—P1—C1—C12	35.0 (5)	C6—P2—C4—C42	−160.9 (4)
C3—P1—C1—C12	158.3 (4)	S2—P2—C4—C42	82.4 (4)
S1—P1—C1—C12	−81.9 (4)	C5—P2—C4—C43	−155.9 (4)
C2—P1—C1—C11	−83.8 (4)	C6—P2—C4—C43	80.0 (5)
C3—P1—C1—C11	39.6 (5)	S2—P2—C4—C43	−36.7 (5)
S1—P1—C1—C11	159.4 (3)	C6—P2—C5—C51	−170.5 (4)
C2—P1—C1—C13	155.4 (4)	C4—P2—C5—C51	63.3 (5)
C3—P1—C1—C13	−81.2 (5)	S2—P2—C5—C51	−52.2 (5)
S1—P1—C1—C13	38.6 (4)	C6—P2—C5—C52	58.3 (5)
C3—P1—C2—C21	169.5 (4)	C4—P2—C5—C52	−67.9 (5)
C1—P1—C2—C21	−64.8 (5)	S2—P2—C5—C52	176.6 (4)
S1—P1—C2—C21	48.7 (5)	C5—P2—C6—C61	174.4 (4)
C3—P1—C2—C22	−60.8 (5)	C4—P2—C6—C61	−56.7 (5)
C1—P1—C2—C22	64.9 (5)	S2—P2—C6—C61	55.9 (5)
S1—P1—C2—C22	178.4 (4)	C5—P2—C6—C62	48.7 (5)
C2—P1—C3—C31	−173.9 (4)	C4—P2—C6—C62	177.6 (4)
C1—P1—C3—C31	57.6 (5)	S2—P2—C6—C62	−69.8 (4)
S1—P1—C3—C31	−55.9 (5)		

### (Disulfane-1,2-diyl)bis(tert-butyldiisopropylphosphonium) bis[tetrachloridoaurate(III)] (1) Hydrogen-bond geometry (Å, º)

**Table:**

*D*—H···*A*	*D*—H	H···*A*	*D*···*A*	*D*—H···*A*
C5—H5···S1	1.00	2.85	3.358 (6)	112
C13—H13*B*···S1	0.98	2.66	3.099 (6)	108
C21—H21*B*···S1	0.98	2.85	3.468 (6)	122
C62—H62*A*···S1	0.98	2.86	3.535 (7)	127
C2—H2···S2	1.00	2.80	3.273 (6)	110
C43—H43*B*···S2	0.98	2.57	3.105 (6)	114
C41—H41*A*···Cl1^i^	0.98	2.95	3.879 (6)	159
C5—H5···Cl2	1.00	2.72	3.521 (6)	138
C51—H51*A*···Cl2	0.98	2.89	3.567 (6)	127
C3—H3···Cl3^ii^	1.00	2.75	3.640 (6)	149
C11—H11*B*···Cl4^ii^	0.98	2.85	3.619 (6)	136
C2—H2···Cl5	1.00	2.58	3.439 (6)	143
C6—H6···Cl8^iii^	1.00	2.73	3.600 (6)	146
C6—H6···Au2^iii^	1.00	3.25	3.851 (6)	121

Symmetry codes: (i) *x*, *y*−1, *z*; (ii) *x*−1, *y*, *z*; (iii) *x*+1, *y*, *z*.

### Bis(di-tert-butylisopropylphosphine sulfide-κS)gold(I) triiodide/diiodidoaurate(I)(0.905/0.095) (2) Crystal data

**Table:**

[Au(C_11_H_25_PS)_2_][AuI_2_]_0.095_(I_3_)_0.905_	*Z* = 2
*M**_r_* = 1025.00	*F*(000) = 969
Triclinic, *P*1	*D*_x_ = 2.098 Mg m^−^^3^
*a* = 8.7116 (3) Å	Mo *K*α radiation, λ = 0.71073 Å
*b* = 12.2732 (4) Å	Cell parameters from 26959 reflections
*c* = 16.2730 (4) Å	θ = 2.3–30.8°
α = 110.531 (3)°	µ = 7.96 mm^−^^1^
β = 90.770 (3)°	*T* = 100 K
γ = 94.514 (3)°	Plate, dichroic yellow/orange
*V* = 1622.76 (9) Å^3^	0.4 × 0.2 × 0.04 mm

### Bis(di-tert-butylisopropylphosphine sulfide-κS)gold(I) triiodide/diiodidoaurate(I)(0.905/0.095) (2) Data collection

**Table:**

Oxford Diffraction Xcalibur, Eos diffractometer	9377 independent reflections
Radiation source: fine-focus sealed tube	8340 reflections with *I* > 2σ(*I*)
Graphite monochromator	*R*_int_ = 0.045
Detector resolution: 16.1419 pixels mm^-1^	θ_max_ = 30.0°, θ_min_ = 2.4°
ω scans	*h* = −12→12
Absorption correction: multi-scan (CrysAlisPro; Rigaku OD, 2015)	*k* = −17→17
*T*_min_ = 0.150, *T*_max_ = 0.750	*l* = −22→22
126632 measured reflections	

### Bis(di-tert-butylisopropylphosphine sulfide-κS)gold(I) triiodide/diiodidoaurate(I)(0.905/0.095) (2) Refinement

**Table:**

Refinement on *F*^2^	Primary atom site location: structure-invariant direct methods
Least-squares matrix: full	Secondary atom site location: difference Fourier map
*R*[*F*^2^ > 2σ(*F*^2^)] = 0.023	Hydrogen site location: inferred from neighbouring sites
*wR*(*F*^2^) = 0.042	H-atom parameters constrained
*S* = 1.05	*w* = 1/[σ^2^(*F*_o_^2^) + (0.0125*P*)^2^ + 2.2996*P*] where *P* = (*F*_o_^2^ + 2*F*_c_^2^)/3
9377 reflections	(Δ/σ)_max_ = 0.001
298 parameters	Δρ_max_ = 1.32 e Å^−^^3^
2 restraints	Δρ_min_ = −0.86 e Å^−^^3^

### Bis(di-tert-butylisopropylphosphine sulfide-κS)gold(I) triiodide/diiodidoaurate(I)(0.905/0.095) (2) Special details

**Table:**

Geometry. All esds (except the esd in the dihedral angle between two l.s. planes) are estimated using the full covariance matrix. The cell esds are taken into account individually in the estimation of esds in distances, angles and torsion angles; correlations between esds in cell parameters are only used when they are defined by crystal symmetry. An approximate (isotropic) treatment of cell esds is used for estimating esds involving l.s. planes.

### Bis(di-tert-butylisopropylphosphine sulfide-κS)gold(I) triiodide/diiodidoaurate(I)(0.905/0.095) (2) Fractional atomic coordinates and isotropic or equivalent isotropic displacement parameters (Å^2^)

**Table:**

	*x*	*y*	*z*	*U*_iso_*/*U*_eq_	Occ. (<1)
Au1	0.50373 (2)	0.25698 (2)	0.24984 (2)	0.01671 (3)	
P1	0.25872 (7)	0.28505 (5)	0.09266 (4)	0.01014 (11)	
P2	0.74234 (7)	0.21812 (5)	0.40465 (4)	0.01133 (11)	
S1	0.28030 (7)	0.18234 (5)	0.16611 (4)	0.01590 (12)	
S2	0.73098 (8)	0.32379 (6)	0.33205 (4)	0.01921 (13)	
C1	0.0611 (3)	0.2381 (2)	0.03802 (16)	0.0140 (5)	
C2	0.4164 (3)	0.2634 (2)	0.01171 (16)	0.0140 (5)	
C3	0.2788 (3)	0.4396 (2)	0.16651 (16)	0.0156 (5)	
H3	0.382485	0.451579	0.197043	0.019*	
C4	0.9522 (3)	0.2253 (2)	0.43563 (17)	0.0159 (5)	
C5	0.6188 (3)	0.2727 (2)	0.50218 (16)	0.0168 (5)	
C6	0.6627 (3)	0.0689 (2)	0.33718 (16)	0.0166 (5)	
H6	0.554657	0.077551	0.320433	0.020*	
C11	−0.0546 (3)	0.2196 (2)	0.10396 (18)	0.0206 (5)	
H11A	−0.157254	0.195041	0.074875	0.031*	
H11B	−0.058100	0.292833	0.153770	0.031*	
H11C	−0.021949	0.159041	0.125072	0.031*	
C12	0.0041 (3)	0.3307 (2)	0.00355 (17)	0.0195 (5)	
H12A	0.083851	0.352531	−0.031180	0.029*	
H12B	−0.017496	0.399994	0.053311	0.029*	
H12C	−0.090092	0.298493	−0.033373	0.029*	
C13	0.0634 (3)	0.1205 (2)	−0.03795 (17)	0.0196 (5)	
H13A	0.109107	0.064376	−0.016607	0.029*	
H13B	0.124738	0.131430	−0.084949	0.029*	
H13C	−0.042230	0.090662	−0.060705	0.029*	
C21	0.3751 (3)	0.2972 (2)	−0.06809 (16)	0.0166 (5)	
H21A	0.352971	0.379306	−0.047848	0.025*	
H21B	0.284130	0.247760	−0.100196	0.025*	
H21C	0.462025	0.286065	−0.106962	0.025*	
C22	0.4547 (3)	0.1350 (2)	−0.02038 (17)	0.0196 (5)	
H22A	0.536933	0.124123	−0.062301	0.029*	
H22B	0.362600	0.083924	−0.049113	0.029*	
H22C	0.488982	0.115234	0.029880	0.029*	
C23	0.5632 (3)	0.3387 (2)	0.05888 (17)	0.0188 (5)	
H23A	0.648855	0.321835	0.019198	0.028*	
H23B	0.588313	0.320544	0.111294	0.028*	
H23C	0.545850	0.421498	0.076196	0.028*	
C31	0.1634 (3)	0.4645 (2)	0.23959 (18)	0.0236 (6)	
H31A	0.190894	0.542305	0.283457	0.035*	
H31B	0.165803	0.405732	0.267569	0.035*	
H31C	0.059497	0.461354	0.214498	0.035*	
C32	0.2801 (3)	0.5325 (2)	0.12362 (18)	0.0211 (5)	
H32A	0.176262	0.533646	0.100183	0.032*	
H32B	0.351779	0.514124	0.075709	0.032*	
H32C	0.313120	0.609304	0.167509	0.032*	
C41	1.0466 (3)	0.2156 (3)	0.35397 (19)	0.0278 (6)	
H41A	1.156304	0.216421	0.368599	0.042*	
H41B	1.030721	0.281864	0.335385	0.042*	
H41C	1.012700	0.142513	0.306155	0.042*	
C42	0.9882 (3)	0.1252 (2)	0.46698 (18)	0.0195 (5)	
H42A	1.096259	0.136446	0.488027	0.029*	
H42B	0.969912	0.050530	0.418066	0.029*	
H42C	0.921310	0.124748	0.514831	0.029*	
C43	1.0022 (3)	0.3436 (2)	0.5072 (2)	0.0270 (6)	
H43A	0.954964	0.347168	0.562308	0.040*	
H43B	0.968906	0.406580	0.489182	0.040*	
H43C	1.114691	0.352518	0.515839	0.040*	
C51	0.6605 (3)	0.2279 (2)	0.57614 (16)	0.0183 (5)	
H51A	0.589942	0.255736	0.624005	0.027*	
H51B	0.766554	0.257170	0.598275	0.027*	
H51C	0.651552	0.142307	0.553280	0.027*	
C52	0.6331 (4)	0.4070 (2)	0.53822 (19)	0.0274 (6)	
H52A	0.564911	0.434864	0.587267	0.041*	
H52B	0.603669	0.435682	0.491623	0.041*	
H52C	0.739931	0.436163	0.558727	0.041*	
C53	0.4496 (3)	0.2314 (3)	0.47060 (18)	0.0266 (6)	
H53A	0.435719	0.145845	0.449274	0.040*	
H53B	0.423155	0.258898	0.422923	0.040*	
H53C	0.382287	0.263132	0.519520	0.040*	
C61	0.7416 (4)	0.0194 (2)	0.24994 (17)	0.0262 (6)	
H61A	0.844467	−0.001093	0.260923	0.039*	
H61B	0.750922	0.078131	0.221762	0.039*	
H61C	0.680021	−0.050582	0.211308	0.039*	
C62	0.6471 (3)	−0.0223 (2)	0.38232 (18)	0.0231 (6)	
H62A	0.596151	−0.095629	0.341325	0.035*	
H62B	0.585472	0.006545	0.434284	0.035*	
H62C	0.749663	−0.035814	0.400115	0.035*	
I1	1.24962 (4)	0.90557 (3)	0.24034 (3)	0.02342 (7)	0.9052 (15)
I2	0.98122 (13)	0.75765 (10)	0.25759 (8)	0.01456 (8)	0.9052 (15)
I3	0.71555 (5)	0.60471 (3)	0.27064 (2)	0.02255 (7)	0.9052 (15)
I1'	1.2208 (6)	0.8932 (4)	0.2456 (3)	0.0290 (6)*	0.0948 (15)
Au2	0.9862 (10)	0.7607 (7)	0.2573 (6)	0.0290 (6)*	0.0948 (15)
I3'	0.7616 (5)	0.6281 (3)	0.26689 (19)	0.0290 (6)*	0.0948 (15)

### Bis(di-tert-butylisopropylphosphine sulfide-κS)gold(I) triiodide/diiodidoaurate(I)(0.905/0.095) (2) Atomic displacement parameters (Å^2^)

**Table:**

	*U*^11^	*U*^22^	*U*^33^	*U*^12^	*U*^13^	*U*^23^
Au1	0.01840 (5)	0.02176 (5)	0.01299 (5)	0.00597 (4)	−0.00120 (3)	0.00906 (4)
P1	0.0110 (3)	0.0108 (3)	0.0092 (3)	0.0018 (2)	0.0004 (2)	0.0040 (2)
P2	0.0110 (3)	0.0137 (3)	0.0106 (3)	0.0011 (2)	−0.0003 (2)	0.0059 (2)
S1	0.0183 (3)	0.0178 (3)	0.0156 (3)	0.0018 (2)	−0.0006 (2)	0.0107 (2)
S2	0.0204 (3)	0.0219 (3)	0.0207 (3)	0.0000 (2)	−0.0036 (2)	0.0148 (3)
C1	0.0121 (11)	0.0169 (11)	0.0138 (12)	0.0004 (9)	−0.0014 (9)	0.0065 (10)
C2	0.0146 (11)	0.0165 (11)	0.0126 (11)	0.0041 (9)	0.0044 (9)	0.0065 (10)
C3	0.0189 (12)	0.0130 (11)	0.0131 (12)	0.0009 (9)	0.0017 (9)	0.0023 (9)
C4	0.0106 (11)	0.0205 (12)	0.0191 (13)	−0.0003 (9)	−0.0024 (9)	0.0105 (10)
C5	0.0143 (11)	0.0240 (13)	0.0126 (12)	0.0064 (10)	0.0016 (9)	0.0060 (10)
C6	0.0184 (12)	0.0175 (12)	0.0120 (12)	−0.0015 (9)	−0.0023 (9)	0.0037 (10)
C11	0.0161 (12)	0.0262 (14)	0.0203 (13)	−0.0012 (10)	0.0027 (10)	0.0097 (11)
C12	0.0171 (12)	0.0249 (13)	0.0192 (13)	0.0043 (10)	−0.0003 (10)	0.0105 (11)
C13	0.0185 (12)	0.0207 (13)	0.0168 (13)	−0.0034 (10)	−0.0020 (10)	0.0042 (11)
C21	0.0181 (12)	0.0194 (12)	0.0142 (12)	0.0031 (10)	0.0027 (9)	0.0080 (10)
C22	0.0239 (13)	0.0184 (12)	0.0174 (13)	0.0079 (10)	0.0045 (10)	0.0060 (10)
C23	0.0130 (11)	0.0246 (13)	0.0202 (13)	0.0019 (10)	0.0025 (10)	0.0094 (11)
C31	0.0331 (15)	0.0180 (13)	0.0170 (13)	0.0048 (11)	0.0087 (11)	0.0019 (11)
C32	0.0269 (14)	0.0145 (12)	0.0217 (14)	0.0028 (10)	0.0033 (11)	0.0058 (11)
C41	0.0158 (13)	0.0470 (18)	0.0287 (16)	0.0034 (12)	0.0048 (11)	0.0232 (14)
C42	0.0176 (12)	0.0228 (13)	0.0203 (13)	0.0065 (10)	−0.0007 (10)	0.0093 (11)
C43	0.0204 (14)	0.0230 (14)	0.0372 (17)	−0.0049 (11)	−0.0094 (12)	0.0121 (13)
C51	0.0174 (12)	0.0273 (13)	0.0116 (12)	0.0039 (10)	0.0010 (9)	0.0080 (11)
C52	0.0372 (17)	0.0251 (14)	0.0205 (14)	0.0170 (12)	0.0006 (12)	0.0058 (12)
C53	0.0153 (13)	0.0469 (18)	0.0176 (14)	0.0074 (12)	0.0020 (10)	0.0104 (13)
C61	0.0390 (17)	0.0222 (14)	0.0138 (13)	0.0059 (12)	0.0021 (12)	0.0010 (11)
C62	0.0299 (15)	0.0169 (12)	0.0224 (14)	−0.0052 (11)	−0.0029 (11)	0.0085 (11)
I1	0.02075 (16)	0.02565 (14)	0.02560 (13)	−0.00244 (11)	−0.00245 (11)	0.01228 (10)
I2	0.01842 (14)	0.01515 (13)	0.00992 (10)	0.00573 (9)	−0.00115 (8)	0.00336 (8)
I3	0.02188 (15)	0.02522 (12)	0.01982 (11)	−0.00236 (10)	−0.00075 (9)	0.00805 (9)

### Bis(di-tert-butylisopropylphosphine sulfide-κS)gold(I) triiodide/diiodidoaurate(I)(0.905/0.095) (2) Geometric parameters (Å, º)

**Table:**

Au1—S1	2.2928 (6)	C22—H22A	0.9800
Au1—S2	2.2990 (7)	C22—H22B	0.9800
P1—C3	1.848 (2)	C22—H22C	0.9800
P1—C1	1.879 (2)	C23—H23A	0.9800
P1—C2	1.885 (2)	C23—H23B	0.9800
P1—S1	2.0350 (8)	C23—H23C	0.9800
P2—C6	1.846 (3)	C31—H31A	0.9800
P2—C4	1.877 (2)	C31—H31B	0.9800
P2—C5	1.881 (2)	C31—H31C	0.9800
P2—S2	2.0444 (8)	C32—H32A	0.9800
C1—C13	1.538 (4)	C32—H32B	0.9800
C1—C12	1.544 (3)	C32—H32C	0.9800
C1—C11	1.544 (3)	C41—H41A	0.9800
C2—C23	1.539 (3)	C41—H41B	0.9800
C2—C21	1.542 (3)	C41—H41C	0.9800
C2—C22	1.543 (3)	C42—H42A	0.9800
C3—C32	1.531 (3)	C42—H42B	0.9800
C3—C31	1.531 (3)	C42—H42C	0.9800
C3—H3	1.0000	C43—H43A	0.9800
C4—C43	1.535 (4)	C43—H43B	0.9800
C4—C42	1.537 (3)	C43—H43C	0.9800
C4—C41	1.546 (4)	C51—H51A	0.9800
C5—C51	1.538 (3)	C51—H51B	0.9800
C5—C52	1.538 (4)	C51—H51C	0.9800
C5—C53	1.541 (4)	C52—H52A	0.9800
C6—C61	1.531 (4)	C52—H52B	0.9800
C6—C62	1.538 (3)	C52—H52C	0.9800
C6—H6	1.0000	C53—H53A	0.9800
C11—H11A	0.9800	C53—H53B	0.9800
C11—H11B	0.9800	C53—H53C	0.9800
C11—H11C	0.9800	C61—H61A	0.9800
C12—H12A	0.9800	C61—H61B	0.9800
C12—H12B	0.9800	C61—H61C	0.9800
C12—H12C	0.9800	C62—H62A	0.9800
C13—H13A	0.9800	C62—H62B	0.9800
C13—H13B	0.9800	C62—H62C	0.9800
C13—H13C	0.9800	I1—I2	2.9238 (11)
C21—H21A	0.9800	I2—I3	2.9213 (11)
C21—H21B	0.9800	I1'—Au2	2.561 (9)
C21—H21C	0.9800	Au2—I3'	2.488 (9)
			
S1—Au1—S2	177.53 (2)	C2—C22—H22A	109.5
C3—P1—C1	112.59 (11)	C2—C22—H22B	109.5
C3—P1—C2	108.03 (11)	H22A—C22—H22B	109.5
C1—P1—C2	112.88 (11)	C2—C22—H22C	109.5
C3—P1—S1	108.65 (8)	H22A—C22—H22C	109.5
C1—P1—S1	104.29 (8)	H22B—C22—H22C	109.5
C2—P1—S1	110.30 (8)	C2—C23—H23A	109.5
C6—P2—C4	112.49 (12)	C2—C23—H23B	109.5
C6—P2—C5	107.84 (12)	H23A—C23—H23B	109.5
C4—P2—C5	113.30 (11)	C2—C23—H23C	109.5
C6—P2—S2	108.92 (8)	H23A—C23—H23C	109.5
C4—P2—S2	105.29 (8)	H23B—C23—H23C	109.5
C5—P2—S2	108.89 (8)	C3—C31—H31A	109.5
P1—S1—Au1	104.94 (3)	C3—C31—H31B	109.5
P2—S2—Au1	104.09 (3)	H31A—C31—H31B	109.5
C13—C1—C12	110.1 (2)	C3—C31—H31C	109.5
C13—C1—C11	107.3 (2)	H31A—C31—H31C	109.5
C12—C1—C11	108.4 (2)	H31B—C31—H31C	109.5
C13—C1—P1	109.70 (17)	C3—C32—H32A	109.5
C12—C1—P1	111.27 (17)	C3—C32—H32B	109.5
C11—C1—P1	110.06 (17)	H32A—C32—H32B	109.5
C23—C2—C21	108.54 (19)	C3—C32—H32C	109.5
C23—C2—C22	107.1 (2)	H32A—C32—H32C	109.5
C21—C2—C22	109.4 (2)	H32B—C32—H32C	109.5
C23—C2—P1	108.77 (17)	C4—C41—H41A	109.5
C21—C2—P1	113.15 (16)	C4—C41—H41B	109.5
C22—C2—P1	109.72 (16)	H41A—C41—H41B	109.5
C32—C3—C31	110.3 (2)	C4—C41—H41C	109.5
C32—C3—P1	117.22 (18)	H41A—C41—H41C	109.5
C31—C3—P1	112.25 (17)	H41B—C41—H41C	109.5
C32—C3—H3	105.3	C4—C42—H42A	109.5
C31—C3—H3	105.3	C4—C42—H42B	109.5
P1—C3—H3	105.3	H42A—C42—H42B	109.5
C43—C4—C42	110.2 (2)	C4—C42—H42C	109.5
C43—C4—C41	108.0 (2)	H42A—C42—H42C	109.5
C42—C4—C41	108.2 (2)	H42B—C42—H42C	109.5
C43—C4—P2	109.77 (18)	C4—C43—H43A	109.5
C42—C4—P2	112.05 (17)	C4—C43—H43B	109.5
C41—C4—P2	108.55 (17)	H43A—C43—H43B	109.5
C51—C5—C52	109.4 (2)	C4—C43—H43C	109.5
C51—C5—C53	109.3 (2)	H43A—C43—H43C	109.5
C52—C5—C53	107.4 (2)	H43B—C43—H43C	109.5
C51—C5—P2	112.75 (17)	C5—C51—H51A	109.5
C52—C5—P2	109.88 (18)	C5—C51—H51B	109.5
C53—C5—P2	107.95 (17)	H51A—C51—H51B	109.5
C61—C6—C62	110.2 (2)	C5—C51—H51C	109.5
C61—C6—P2	113.15 (18)	H51A—C51—H51C	109.5
C62—C6—P2	117.28 (18)	H51B—C51—H51C	109.5
C61—C6—H6	105.0	C5—C52—H52A	109.5
C62—C6—H6	105.0	C5—C52—H52B	109.5
P2—C6—H6	105.0	H52A—C52—H52B	109.5
C1—C11—H11A	109.5	C5—C52—H52C	109.5
C1—C11—H11B	109.5	H52A—C52—H52C	109.5
H11A—C11—H11B	109.5	H52B—C52—H52C	109.5
C1—C11—H11C	109.5	C5—C53—H53A	109.5
H11A—C11—H11C	109.5	C5—C53—H53B	109.5
H11B—C11—H11C	109.5	H53A—C53—H53B	109.5
C1—C12—H12A	109.5	C5—C53—H53C	109.5
C1—C12—H12B	109.5	H53A—C53—H53C	109.5
H12A—C12—H12B	109.5	H53B—C53—H53C	109.5
C1—C12—H12C	109.5	C6—C61—H61A	109.5
H12A—C12—H12C	109.5	C6—C61—H61B	109.5
H12B—C12—H12C	109.5	H61A—C61—H61B	109.5
C1—C13—H13A	109.5	C6—C61—H61C	109.5
C1—C13—H13B	109.5	H61A—C61—H61C	109.5
H13A—C13—H13B	109.5	H61B—C61—H61C	109.5
C1—C13—H13C	109.5	C6—C62—H62A	109.5
H13A—C13—H13C	109.5	C6—C62—H62B	109.5
H13B—C13—H13C	109.5	H62A—C62—H62B	109.5
C2—C21—H21A	109.5	C6—C62—H62C	109.5
C2—C21—H21B	109.5	H62A—C62—H62C	109.5
H21A—C21—H21B	109.5	H62B—C62—H62C	109.5
C2—C21—H21C	109.5	I3—I2—I1	178.44 (5)
H21A—C21—H21C	109.5	I3'—Au2—I1'	178.7 (5)
H21B—C21—H21C	109.5		
			
C3—P1—S1—Au1	−51.13 (9)	C1—P1—C3—C31	59.1 (2)
C1—P1—S1—Au1	−171.42 (8)	C2—P1—C3—C31	−175.59 (18)
C2—P1—S1—Au1	67.11 (9)	S1—P1—C3—C31	−55.9 (2)
C6—P2—S2—Au1	36.45 (9)	C6—P2—C4—C43	−167.64 (17)
C4—P2—S2—Au1	157.30 (9)	C5—P2—C4—C43	−45.0 (2)
C5—P2—S2—Au1	−80.90 (9)	S2—P2—C4—C43	73.87 (18)
C3—P1—C1—C13	166.40 (16)	C6—P2—C4—C42	−44.9 (2)
C2—P1—C1—C13	43.75 (19)	C5—P2—C4—C42	77.7 (2)
S1—P1—C1—C13	−75.99 (16)	S2—P2—C4—C42	−163.40 (16)
C3—P1—C1—C12	44.3 (2)	C6—P2—C4—C41	74.6 (2)
C2—P1—C1—C12	−78.30 (19)	C5—P2—C4—C41	−162.80 (18)
S1—P1—C1—C12	161.96 (15)	S2—P2—C4—C41	−43.91 (19)
C3—P1—C1—C11	−75.81 (19)	C6—P2—C5—C51	81.5 (2)
C2—P1—C1—C11	161.54 (16)	C4—P2—C5—C51	−43.7 (2)
S1—P1—C1—C11	41.80 (18)	S2—P2—C5—C51	−160.47 (16)
C3—P1—C2—C23	36.60 (19)	C6—P2—C5—C52	−156.24 (18)
C1—P1—C2—C23	161.77 (16)	C4—P2—C5—C52	78.59 (19)
S1—P1—C2—C23	−82.01 (16)	S2—P2—C5—C52	−38.20 (19)
C3—P1—C2—C21	−84.10 (19)	C6—P2—C5—C53	−39.4 (2)
C1—P1—C2—C21	41.1 (2)	C4—P2—C5—C53	−164.53 (18)
S1—P1—C2—C21	157.29 (15)	S2—P2—C5—C53	78.68 (19)
C3—P1—C2—C22	153.42 (17)	C4—P2—C6—C61	−61.0 (2)
C1—P1—C2—C22	−81.42 (19)	C5—P2—C6—C61	173.38 (18)
S1—P1—C2—C22	34.80 (19)	S2—P2—C6—C61	55.4 (2)
C1—P1—C3—C32	−70.1 (2)	C4—P2—C6—C62	69.0 (2)
C2—P1—C3—C32	55.2 (2)	C5—P2—C6—C62	−56.7 (2)
S1—P1—C3—C32	174.88 (17)	S2—P2—C6—C62	−174.68 (18)

### Bis(di-tert-butylisopropylphosphine sulfide-κS)gold(I) triiodide/diiodidoaurate(I)(0.905/0.095) (2) Hydrogen-bond geometry (Å, º)

**Table:**

*D*—H···*A*	*D*—H	H···*A*	*D*···*A*	*D*—H···*A*
C23—H23*B*···Au1	0.98	2.72	3.616 (3)	152
C53—H53*B*···Au1	0.98	2.90	3.747 (3)	145
C22—H22*C*···S1	0.98	2.82	3.299 (3)	111
C11—H11*C*···S1	0.98	2.68	3.194 (3)	113
C52—H52*B*···S2	0.98	2.77	3.292 (3)	114
C41—H41*B*···S2	0.98	2.70	3.214 (3)	113
C3—H3···I3	1.00	3.29	4.202 (3)	152
C6—H6···I1^i^	1.00	3.21	3.993 (3)	136
C51—H51*B*···I2^ii^	0.98	3.25	4.055 (3)	140
C61—H61*A*···I2^iii^	0.98	3.26	4.003 (3)	134

Symmetry codes: (i) *x*−1, *y*−1, *z*; (ii) −*x*+2, −*y*+1, −*z*+1; (iii) *x*, *y*−1, *z*.

### Bis(tert-butyldiisopropylphosphine sulfide-κS)gold(I) triiodide/diiodidoaurate(I)(0.875/0.125) (3) Crystal data

**Table:**

[Au(C_10_H_23_PS)_2_][AuI_2_]_0.125_(I_3_)_0.875_	*F*(000) = 1877
*M**_r_* = 999.05	*D*_x_ = 2.128 Mg m^−^^3^
Monoclinic, *P*2_1_/*n*	Mo *K*α radiation, λ = 0.71073 Å
*a* = 11.6180 (2) Å	Cell parameters from 25881 reflections
*b* = 11.8894 (3) Å	θ = 2.4–29.3°
*c* = 22.5768 (5) Å	µ = 8.39 mm^−^^1^
β = 90.090 (2)°	*T* = 100 K
*V* = 3118.54 (12) Å^3^	Plate, orange
*Z* = 4	0.15 × 0.08 × 0.05 mm

### Bis(tert-butyldiisopropylphosphine sulfide-κS)gold(I) triiodide/diiodidoaurate(I)(0.875/0.125) (3) Data collection

**Table:**

Oxford Diffraction Xcalibur, Eos diffractometer	7725 independent reflections
Radiation source: fine-focus sealed tube	7218 reflections with *I* > 2σ(*I*)
Graphite monochromator	*R*_int_ = 0.072
Detector resolution: 16.1419 pixels mm^-1^	θ_max_ = 28.3°, θ_min_ = 2.5°
ω scan	*h* = −15→15
Absorption correction: multi-scan (CrysAlisPro; Rigaku OD, 2015)	*k* = −15→15
*T*_min_ = 0.565, *T*_max_ = 1.000	*l* = −30→30
142434 measured reflections	

### Bis(tert-butyldiisopropylphosphine sulfide-κS)gold(I) triiodide/diiodidoaurate(I)(0.875/0.125) (3) Refinement

**Table:**

Refinement on *F*^2^	Primary atom site location: structure-invariant direct methods
Least-squares matrix: full	Secondary atom site location: difference Fourier map
*R*[*F*^2^ > 2σ(*F*^2^)] = 0.022	Hydrogen site location: inferred from neighbouring sites
*wR*(*F*^2^) = 0.041	H-atom parameters constrained
*S* = 1.08	*w* = 1/[σ^2^(*F*_o_^2^) + (0.012*P*)^2^ + 2.6204*P*] where *P* = (*F*_o_^2^ + 2*F*_c_^2^)/3
7725 reflections	(Δ/σ)_max_ = 0.002
281 parameters	Δρ_max_ = 0.65 e Å^−^^3^
7 restraints	Δρ_min_ = −0.79 e Å^−^^3^

### Bis(tert-butyldiisopropylphosphine sulfide-κS)gold(I) triiodide/diiodidoaurate(I)(0.875/0.125) (3) Fractional atomic coordinates and isotropic or equivalent isotropic displacement parameters (Å^2^)

**Table:**

	*x*	*y*	*z*	*U*_iso_*/*U*_eq_	Occ. (<1)
Au1	0.50279 (2)	0.31391 (2)	0.72024 (2)	0.01620 (4)	
S1	0.69445 (8)	0.29078 (8)	0.69963 (4)	0.01842 (19)	
S2	0.30896 (8)	0.33250 (8)	0.73637 (4)	0.01784 (19)	
P1	0.70782 (7)	0.37066 (7)	0.62071 (4)	0.01298 (18)	
P2	0.29453 (7)	0.36368 (7)	0.82448 (4)	0.01231 (18)	
C1	0.8580 (3)	0.3484 (3)	0.59402 (18)	0.0183 (8)	
C2	0.5977 (3)	0.3180 (3)	0.56939 (16)	0.0157 (7)	
H2	0.523070	0.344337	0.586492	0.019*	
C3	0.6831 (3)	0.5237 (3)	0.62619 (17)	0.0184 (8)	
H3	0.708886	0.557766	0.587947	0.022*	
C4	0.1440 (3)	0.4127 (3)	0.83670 (17)	0.0173 (8)	
C5	0.4047 (3)	0.4637 (3)	0.84895 (17)	0.0186 (8)	
H5	0.479207	0.422739	0.843695	0.022*	
C6	0.3210 (3)	0.2363 (3)	0.86908 (18)	0.0182 (8)	
H6	0.301326	0.255156	0.911036	0.022*	
C11	0.8678 (3)	0.2328 (3)	0.56431 (19)	0.0242 (9)	
H11A	0.821607	0.232035	0.527957	0.036*	
H11B	0.839527	0.174663	0.591428	0.036*	
H11C	0.948514	0.217722	0.554571	0.036*	
C12	0.8917 (3)	0.4385 (3)	0.54867 (18)	0.0223 (8)	
H12A	0.968954	0.422496	0.533443	0.033*	
H12B	0.891374	0.512570	0.567741	0.033*	
H12C	0.836422	0.438113	0.515857	0.033*	
C13	0.9421 (3)	0.3517 (3)	0.64673 (19)	0.0249 (9)	
H13A	1.020633	0.338881	0.632532	0.037*	
H13B	0.921362	0.292996	0.675242	0.037*	
H13C	0.937713	0.425483	0.665989	0.037*	
C21	0.5861 (3)	0.1897 (3)	0.56617 (19)	0.0223 (8)	
H21A	0.511241	0.170083	0.548966	0.033*	
H21B	0.591926	0.157960	0.606126	0.033*	
H21C	0.647696	0.158990	0.541359	0.033*	
C22	0.6022 (3)	0.3709 (3)	0.50792 (17)	0.0221 (8)	
H22A	0.667878	0.340186	0.486028	0.033*	
H22B	0.610884	0.452583	0.511689	0.033*	
H22C	0.530858	0.353938	0.486524	0.033*	
C31	0.7548 (3)	0.5775 (3)	0.67531 (18)	0.0274 (9)	
H31A	0.735080	0.657390	0.678571	0.041*	
H31B	0.836729	0.569824	0.665931	0.041*	
H31C	0.738543	0.539753	0.712978	0.041*	
C32	0.5570 (3)	0.5551 (3)	0.63429 (18)	0.0217 (8)	
H32A	0.529676	0.526496	0.672473	0.033*	
H32B	0.511241	0.521909	0.602253	0.033*	
H32C	0.548912	0.637148	0.633361	0.033*	
C41	0.1075 (3)	0.3948 (3)	0.90104 (18)	0.0231 (9)	
H41A	0.161397	0.433791	0.927401	0.035*	
H41B	0.107879	0.314167	0.910108	0.035*	
H41C	0.029807	0.424898	0.906870	0.035*	
C42	0.1359 (3)	0.5382 (3)	0.8217 (2)	0.0286 (10)	
H42A	0.054917	0.561236	0.821054	0.043*	
H42B	0.170308	0.551780	0.782758	0.043*	
H42C	0.177352	0.581892	0.851756	0.043*	
C43	0.0618 (3)	0.3484 (4)	0.79507 (19)	0.0276 (9)	
H43A	0.069143	0.267450	0.802188	0.041*	
H43B	0.081701	0.365046	0.753812	0.041*	
H43C	−0.017612	0.371995	0.802714	0.041*	
C51	0.4171 (3)	0.5701 (3)	0.81099 (19)	0.0257 (9)	
H51A	0.359587	0.625680	0.823251	0.039*	
H51B	0.405273	0.551027	0.769194	0.039*	
H51C	0.494411	0.601595	0.816290	0.039*	
C52	0.4001 (4)	0.4915 (4)	0.91501 (19)	0.0302 (10)	
H52A	0.472108	0.527942	0.926986	0.045*	
H52B	0.389726	0.422049	0.937759	0.045*	
H52C	0.335405	0.542384	0.922639	0.045*	
C61	0.2431 (3)	0.1387 (3)	0.85035 (18)	0.0229 (8)	
H61A	0.253593	0.123628	0.808022	0.034*	
H61B	0.162590	0.158479	0.857895	0.034*	
H61C	0.263343	0.071334	0.873153	0.034*	
C62	0.4462 (3)	0.1986 (3)	0.8684 (2)	0.0315 (10)	
H62A	0.453948	0.128166	0.890557	0.047*	
H62B	0.494377	0.256635	0.886769	0.047*	
H62C	0.470913	0.186804	0.827367	0.047*	
I1	0.23728 (2)	0.06220 (3)	0.35268 (2)	0.02167 (11)	0.8748 (15)
I2	0.25192 (5)	0.19148 (6)	0.46007 (3)	0.01730 (9)	0.8748 (15)
I3	0.26412 (15)	0.32860 (13)	0.56798 (7)	0.0260 (4)	0.8748 (15)
I1'	0.2398 (2)	0.0894 (2)	0.37747 (17)	0.0243 (7)*	0.1252 (15)
Au2'	0.2515 (3)	0.2104 (2)	0.47095 (13)	0.0190 (6)*	0.1252 (15)
I3'	0.2642 (8)	0.3284 (6)	0.5696 (4)	0.0051 (17)*	0.1252 (15)

### Bis(tert-butyldiisopropylphosphine sulfide-κS)gold(I) triiodide/diiodidoaurate(I)(0.875/0.125) (3) Atomic displacement parameters (Å^2^)

**Table:**

	*U*^11^	*U*^22^	*U*^33^	*U*^12^	*U*^13^	*U*^23^
Au1	0.01582 (6)	0.01614 (6)	0.01666 (7)	−0.00100 (5)	0.00516 (6)	−0.00075 (5)
S1	0.0160 (4)	0.0230 (5)	0.0162 (5)	0.0022 (3)	0.0027 (4)	0.0053 (4)
S2	0.0158 (4)	0.0231 (5)	0.0147 (5)	−0.0014 (3)	0.0022 (3)	−0.0016 (4)
P1	0.0108 (4)	0.0132 (4)	0.0150 (5)	0.0011 (3)	0.0009 (3)	0.0014 (3)
P2	0.0117 (4)	0.0112 (4)	0.0140 (5)	−0.0004 (3)	0.0011 (3)	0.0010 (3)
C1	0.0133 (16)	0.0181 (18)	0.023 (2)	0.0007 (14)	0.0072 (15)	0.0050 (15)
C2	0.0131 (16)	0.0184 (18)	0.0155 (19)	0.0008 (13)	−0.0006 (14)	−0.0015 (15)
C3	0.0197 (18)	0.0144 (17)	0.021 (2)	0.0025 (14)	0.0019 (15)	0.0002 (15)
C4	0.0134 (17)	0.0187 (18)	0.020 (2)	0.0044 (13)	0.0033 (14)	0.0037 (15)
C5	0.0164 (17)	0.0152 (17)	0.024 (2)	−0.0033 (14)	0.0005 (15)	−0.0031 (15)
C6	0.0172 (17)	0.0167 (18)	0.021 (2)	0.0014 (14)	−0.0006 (15)	0.0047 (15)
C11	0.0215 (19)	0.022 (2)	0.029 (2)	0.0075 (15)	0.0080 (17)	0.0015 (17)
C12	0.0166 (18)	0.024 (2)	0.026 (2)	−0.0021 (15)	0.0065 (16)	0.0071 (17)
C13	0.0100 (17)	0.036 (2)	0.029 (2)	0.0033 (15)	0.0007 (16)	0.0076 (18)
C21	0.0220 (19)	0.0195 (19)	0.025 (2)	−0.0034 (15)	−0.0042 (16)	−0.0013 (16)
C22	0.0208 (19)	0.025 (2)	0.021 (2)	0.0048 (15)	−0.0015 (16)	0.0039 (16)
C31	0.030 (2)	0.0191 (19)	0.033 (2)	0.0008 (17)	−0.0072 (19)	−0.0061 (16)
C32	0.0229 (19)	0.0154 (18)	0.027 (2)	0.0075 (15)	0.0035 (16)	0.0016 (16)
C41	0.0203 (19)	0.027 (2)	0.022 (2)	0.0048 (16)	0.0061 (16)	0.0044 (17)
C42	0.028 (2)	0.024 (2)	0.034 (3)	0.0096 (17)	0.0079 (19)	0.0078 (18)
C43	0.0157 (18)	0.040 (2)	0.027 (2)	0.0017 (17)	−0.0030 (16)	0.0026 (19)
C51	0.024 (2)	0.0173 (19)	0.036 (3)	−0.0071 (15)	0.0073 (18)	−0.0012 (17)
C52	0.037 (2)	0.028 (2)	0.025 (2)	−0.0099 (18)	0.0016 (19)	−0.0089 (18)
C61	0.0241 (19)	0.0152 (17)	0.030 (2)	−0.0045 (15)	0.0015 (18)	0.0031 (15)
C62	0.022 (2)	0.026 (2)	0.046 (3)	0.0082 (16)	−0.0012 (19)	0.014 (2)
I1	0.01827 (14)	0.02527 (17)	0.0215 (2)	−0.00122 (12)	−0.00043 (13)	0.00139 (16)
I2	0.01559 (17)	0.0190 (2)	0.0173 (2)	−0.00070 (16)	−0.00028 (17)	0.00326 (19)
I3	0.0210 (4)	0.0270 (4)	0.0300 (5)	0.00035 (12)	0.00129 (14)	−0.00101 (14)

### Bis(tert-butyldiisopropylphosphine sulfide-κS)gold(I) triiodide/diiodidoaurate(I)(0.875/0.125) (3) Geometric parameters (Å, º)

**Table:**

Au1—S1	2.2920 (9)	C21—H21A	0.9800
Au1—S2	2.2924 (9)	C21—H21B	0.9800
S1—P1	2.0251 (13)	C21—H21C	0.9800
S2—P2	2.0308 (13)	C22—H22A	0.9800
P1—C2	1.835 (4)	C22—H22B	0.9800
P1—C3	1.847 (4)	C22—H22C	0.9800
P1—C1	1.866 (3)	C31—H31A	0.9800
P2—C5	1.832 (4)	C31—H31B	0.9800
P2—C6	1.844 (4)	C31—H31C	0.9800
P2—C4	1.865 (3)	C32—H32A	0.9800
C1—C12	1.534 (5)	C32—H32B	0.9800
C1—C11	1.534 (5)	C32—H32C	0.9800
C1—C13	1.539 (5)	C41—H41A	0.9800
C2—C22	1.525 (5)	C41—H41B	0.9800
C2—C21	1.533 (5)	C41—H41C	0.9800
C2—H2	1.0000	C42—H42A	0.9800
C3—C32	1.523 (5)	C42—H42B	0.9800
C3—C31	1.526 (5)	C42—H42C	0.9800
C3—H3	1.0000	C43—H43A	0.9800
C4—C41	1.529 (5)	C43—H43B	0.9800
C4—C42	1.533 (5)	C43—H43C	0.9800
C4—C43	1.541 (5)	C51—H51A	0.9800
C5—C52	1.529 (6)	C51—H51B	0.9800
C5—C51	1.535 (5)	C51—H51C	0.9800
C5—H5	1.0000	C52—H52A	0.9800
C6—C62	1.522 (5)	C52—H52B	0.9800
C6—C61	1.531 (5)	C52—H52C	0.9800
C6—H6	1.0000	C61—H61A	0.9800
C11—H11A	0.9800	C61—H61B	0.9800
C11—H11B	0.9800	C61—H61C	0.9800
C11—H11C	0.9800	C62—H62A	0.9800
C12—H12A	0.9800	C62—H62B	0.9800
C12—H12B	0.9800	C62—H62C	0.9800
C12—H12C	0.9800	I1—I2	2.8755 (7)
C13—H13A	0.9800	I2—I3	2.9347 (17)
C13—H13B	0.9800	I1'—Au2'	2.557 (4)
C13—H13C	0.9800	Au2'—I3'	2.636 (8)
			
S1—Au1—S2	177.07 (4)	C2—C21—H21A	109.5
P1—S1—Au1	101.43 (4)	C2—C21—H21B	109.5
P2—S2—Au1	104.82 (5)	H21A—C21—H21B	109.5
C2—P1—C3	105.67 (17)	C2—C21—H21C	109.5
C2—P1—C1	113.52 (17)	H21A—C21—H21C	109.5
C3—P1—C1	107.89 (16)	H21B—C21—H21C	109.5
C2—P1—S1	109.95 (12)	C2—C22—H22A	109.5
C3—P1—S1	113.02 (13)	C2—C22—H22B	109.5
C1—P1—S1	106.89 (13)	H22A—C22—H22B	109.5
C5—P2—C6	104.62 (17)	C2—C22—H22C	109.5
C5—P2—C4	114.09 (16)	H22A—C22—H22C	109.5
C6—P2—C4	109.35 (16)	H22B—C22—H22C	109.5
C5—P2—S2	110.80 (13)	C3—C31—H31A	109.5
C6—P2—S2	111.79 (13)	C3—C31—H31B	109.5
C4—P2—S2	106.31 (13)	H31A—C31—H31B	109.5
C12—C1—C11	108.4 (3)	C3—C31—H31C	109.5
C12—C1—C13	109.6 (3)	H31A—C31—H31C	109.5
C11—C1—C13	108.3 (3)	H31B—C31—H31C	109.5
C12—C1—P1	110.9 (2)	C3—C32—H32A	109.5
C11—C1—P1	109.8 (2)	C3—C32—H32B	109.5
C13—C1—P1	109.8 (3)	H32A—C32—H32B	109.5
C22—C2—C21	111.7 (3)	C3—C32—H32C	109.5
C22—C2—P1	114.1 (3)	H32A—C32—H32C	109.5
C21—C2—P1	115.5 (3)	H32B—C32—H32C	109.5
C22—C2—H2	104.7	C4—C41—H41A	109.5
C21—C2—H2	104.7	C4—C41—H41B	109.5
P1—C2—H2	104.7	H41A—C41—H41B	109.5
C32—C3—C31	109.5 (3)	C4—C41—H41C	109.5
C32—C3—P1	113.5 (3)	H41A—C41—H41C	109.5
C31—C3—P1	112.1 (3)	H41B—C41—H41C	109.5
C32—C3—H3	107.1	C4—C42—H42A	109.5
C31—C3—H3	107.1	C4—C42—H42B	109.5
P1—C3—H3	107.1	H42A—C42—H42B	109.5
C41—C4—C42	109.2 (3)	C4—C42—H42C	109.5
C41—C4—C43	109.8 (3)	H42A—C42—H42C	109.5
C42—C4—C43	108.1 (3)	H42B—C42—H42C	109.5
C41—C4—P2	111.0 (3)	C4—C43—H43A	109.5
C42—C4—P2	109.2 (2)	C4—C43—H43B	109.5
C43—C4—P2	109.6 (3)	H43A—C43—H43B	109.5
C52—C5—C51	111.7 (3)	C4—C43—H43C	109.5
C52—C5—P2	114.1 (3)	H43A—C43—H43C	109.5
C51—C5—P2	115.6 (3)	H43B—C43—H43C	109.5
C52—C5—H5	104.7	C5—C51—H51A	109.5
C51—C5—H5	104.7	C5—C51—H51B	109.5
P2—C5—H5	104.7	H51A—C51—H51B	109.5
C62—C6—C61	109.7 (3)	C5—C51—H51C	109.5
C62—C6—P2	113.2 (3)	H51A—C51—H51C	109.5
C61—C6—P2	112.0 (3)	H51B—C51—H51C	109.5
C62—C6—H6	107.2	C5—C52—H52A	109.5
C61—C6—H6	107.2	C5—C52—H52B	109.5
P2—C6—H6	107.2	H52A—C52—H52B	109.5
C1—C11—H11A	109.5	C5—C52—H52C	109.5
C1—C11—H11B	109.5	H52A—C52—H52C	109.5
H11A—C11—H11B	109.5	H52B—C52—H52C	109.5
C1—C11—H11C	109.5	C6—C61—H61A	109.5
H11A—C11—H11C	109.5	C6—C61—H61B	109.5
H11B—C11—H11C	109.5	H61A—C61—H61B	109.5
C1—C12—H12A	109.5	C6—C61—H61C	109.5
C1—C12—H12B	109.5	H61A—C61—H61C	109.5
H12A—C12—H12B	109.5	H61B—C61—H61C	109.5
C1—C12—H12C	109.5	C6—C62—H62A	109.5
H12A—C12—H12C	109.5	C6—C62—H62B	109.5
H12B—C12—H12C	109.5	H62A—C62—H62B	109.5
C1—C13—H13A	109.5	C6—C62—H62C	109.5
C1—C13—H13B	109.5	H62A—C62—H62C	109.5
H13A—C13—H13B	109.5	H62B—C62—H62C	109.5
C1—C13—H13C	109.5	I1—I2—I3	178.46 (4)
H13A—C13—H13C	109.5	I1'—Au2'—I3'	177.9 (2)
H13B—C13—H13C	109.5		
			
Au1—S1—P1—C2	−51.38 (13)	C2—P1—C3—C31	170.3 (3)
Au1—S1—P1—C3	66.44 (13)	C1—P1—C3—C31	−67.9 (3)
Au1—S1—P1—C1	−175.01 (13)	S1—P1—C3—C31	50.0 (3)
Au1—S2—P2—C5	42.46 (13)	C5—P2—C4—C41	−80.0 (3)
Au1—S2—P2—C6	−73.81 (13)	C6—P2—C4—C41	36.7 (3)
Au1—S2—P2—C4	166.94 (12)	S2—P2—C4—C41	157.6 (2)
C2—P1—C1—C12	80.1 (3)	C5—P2—C4—C42	40.4 (3)
C3—P1—C1—C12	−36.6 (3)	C6—P2—C4—C42	157.1 (3)
S1—P1—C1—C12	−158.5 (2)	S2—P2—C4—C42	−82.0 (3)
C2—P1—C1—C11	−39.6 (3)	C5—P2—C4—C43	158.6 (3)
C3—P1—C1—C11	−156.3 (3)	C6—P2—C4—C43	−84.7 (3)
S1—P1—C1—C11	81.8 (3)	S2—P2—C4—C43	36.1 (3)
C2—P1—C1—C13	−158.6 (2)	C6—P2—C5—C52	−59.2 (3)
C3—P1—C1—C13	84.7 (3)	C4—P2—C5—C52	60.2 (3)
S1—P1—C1—C13	−37.2 (3)	S2—P2—C5—C52	−179.8 (2)
C3—P1—C2—C22	59.2 (3)	C6—P2—C5—C51	169.2 (3)
C1—P1—C2—C22	−58.9 (3)	C4—P2—C5—C51	−71.4 (3)
S1—P1—C2—C22	−178.6 (2)	S2—P2—C5—C51	48.5 (3)
C3—P1—C2—C21	−169.3 (3)	C5—P2—C6—C62	−49.9 (3)
C1—P1—C2—C21	72.7 (3)	C4—P2—C6—C62	−172.5 (3)
S1—P1—C2—C21	−47.0 (3)	S2—P2—C6—C62	70.1 (3)
C2—P1—C3—C32	45.5 (3)	C5—P2—C6—C61	−174.6 (3)
C1—P1—C3—C32	167.3 (3)	C4—P2—C6—C61	62.8 (3)
S1—P1—C3—C32	−74.7 (3)	S2—P2—C6—C61	−54.7 (3)

### Bis(tert-butyldiisopropylphosphine sulfide-κS)gold(I) triiodide/diiodidoaurate(I)(0.875/0.125) (3) Hydrogen-bond geometry (Å, º)

**Table:**

*D*—H···*A*	*D*—H	H···*A*	*D*···*A*	*D*—H···*A*
C5—H5···I1^i^	1.00	3.01	3.877 (4)	146
C11—H11*B*···I1^ii^	0.98	3.21	4.160 (4)	163
C43—H43*C*···I1^iii^	0.98	3.16	4.130 (4)	169
C3—H3···I2^iv^	1.00	3.21	3.979 (4)	135
C2—H2···I3	1.00	3.04	3.878 (4)	142
C13—H13*A*···I3^v^	0.98	3.19	4.154 (4)	170
C61—H61*C*···I3^vi^	0.98	3.19	4.123 (4)	159
C32—H32*A*···Au1	0.98	2.77	3.520 (4)	134
C62—H62*C*···Au1	0.98	2.88	3.675 (5)	139

Symmetry codes: (i) *x*+1/2, −*y*+1/2, *z*+1/2; (ii) −*x*+1, −*y*, −*z*+1; (iii) *x*−1/2, −*y*+1/2, *z*+1/2; (iv) −*x*+1, −*y*+1, −*z*+1; (v) *x*+1, *y*, *z*; (vi) −*x*+1/2, *y*−1/2, −*z*+3/2.

### Bis(di-tert-butylisopropylphosphine sulfide-κS)gold(I) triiodide bis(iodine) (4) Crystal data

**Table:**

[Au(C_11_H_25_PS)_2_]I_3_·2I_2_	*D*_x_ = 2.486 Mg m^−^^3^
*M**_r_* = 1525.94	Mo *K*α radiation, λ = 0.71073 Å
Orthorhombic, *P**c**c**n*	Cell parameters from 25916 reflections
*a* = 15.0435 (3) Å	θ = 2.2–30.9°
*b* = 15.2629 (3) Å	µ = 9.10 mm^−^^1^
*c* = 17.7574 (3) Å	*T* = 100 K
*V* = 4077.24 (13) Å^3^	Plate, red
*Z* = 4	0.2 × 0.08 × 0.03 mm
*F*(000) = 2776	

### Bis(di-tert-butylisopropylphosphine sulfide-κS)gold(I) triiodide bis(iodine) (4) Data collection

**Table:**

Oxford Diffraction Xcalibur, Eos diffractometer	5958 independent reflections
Radiation source: fine-focus sealed tube	5144 reflections with *I* > 2σ(*I*)
Graphite monochromator	*R*_int_ = 0.053
Detector resolution: 16.1419 pixels mm^-1^	θ_max_ = 30.0°, θ_min_ = 2.2°
ω–scan	*h* = −20→21
Absorption correction: multi-scan (CrysAlisPro; Rigaku OD, 2015)	*k* = −21→21
*T*_min_ = 0.263, *T*_max_ = 0.772	*l* = −25→25
112897 measured reflections	

### Bis(di-tert-butylisopropylphosphine sulfide-κS)gold(I) triiodide bis(iodine) (4) Refinement

**Table:**

Refinement on *F*^2^	Secondary atom site location: difference Fourier map
Least-squares matrix: full	Hydrogen site location: inferred from neighbouring sites
*R*[*F*^2^ > 2σ(*F*^2^)] = 0.021	H-atom parameters constrained
*wR*(*F*^2^) = 0.034	*w* = 1/[σ^2^(*F*_o_^2^) + (0.0083*P*)^2^ + 4.2635*P*] where *P* = (*F*_o_^2^ + 2*F*_c_^2^)/3
*S* = 1.11	(Δ/σ)_max_ = 0.002
5958 reflections	Δρ_max_ = 0.95 e Å^−^^3^
164 parameters	Δρ_min_ = −1.39 e Å^−^^3^
0 restraints	Extinction correction: *SHELXL2019/3* (Sheldrick, 2015), Fc^*^=kFc[1+0.001xFc^2^λ^3^/sin(2θ)]^-1/4^
Primary atom site location: structure-invariant direct methods	Extinction coefficient: 0.000131 (6)

### Bis(di-tert-butylisopropylphosphine sulfide-κS)gold(I) triiodide bis(iodine) (4) Fractional atomic coordinates and isotropic or equivalent isotropic displacement parameters (Å^2^)

**Table:**

	*x*	*y*	*z*	*U*_iso_*/*U*_eq_	
Au1	0.750000	0.250000	0.15374 (2)	0.01526 (3)	
P1	0.52206 (4)	0.26998 (4)	0.12378 (3)	0.01213 (12)	
S1	0.62991 (4)	0.34121 (4)	0.15778 (4)	0.01652 (13)	
C1	0.50319 (17)	0.17284 (17)	0.18685 (14)	0.0174 (5)	
C2	0.42991 (17)	0.35226 (18)	0.12664 (16)	0.0215 (6)	
C3	0.54510 (17)	0.22913 (17)	0.02766 (14)	0.0177 (5)	
H3	0.601348	0.194643	0.032316	0.021*	
C11	0.40722 (18)	0.13816 (19)	0.18186 (16)	0.0240 (6)	
H11A	0.401860	0.084454	0.211760	0.036*	
H11B	0.392583	0.125481	0.129197	0.036*	
H11C	0.366141	0.182486	0.201443	0.036*	
C12	0.5237 (2)	0.1960 (2)	0.26893 (15)	0.0281 (7)	
H12A	0.485685	0.244695	0.284941	0.042*	
H12B	0.586257	0.213254	0.273436	0.042*	
H12C	0.512393	0.144926	0.300951	0.042*	
C13	0.56738 (19)	0.09862 (18)	0.16356 (16)	0.0234 (6)	
H13A	0.628659	0.120345	0.164908	0.035*	
H13B	0.552993	0.079065	0.112429	0.035*	
H13C	0.561196	0.049369	0.198581	0.035*	
C21	0.46094 (19)	0.43973 (19)	0.09287 (18)	0.0292 (7)	
H21A	0.412048	0.482113	0.094614	0.044*	
H21B	0.479192	0.430679	0.040467	0.044*	
H21C	0.511367	0.462191	0.121986	0.044*	
C22	0.34980 (19)	0.3190 (2)	0.0810 (2)	0.0360 (8)	
H22A	0.335341	0.259095	0.096461	0.054*	
H22B	0.364506	0.319708	0.027198	0.054*	
H22C	0.298520	0.357188	0.090120	0.054*	
C23	0.4025 (2)	0.3707 (2)	0.20844 (18)	0.0347 (8)	
H23A	0.454940	0.387966	0.237631	0.052*	
H23B	0.376399	0.317743	0.230446	0.052*	
H23C	0.358667	0.418198	0.209440	0.052*	
C31	0.5667 (2)	0.3027 (2)	−0.02837 (16)	0.0321 (7)	
H31A	0.595565	0.277747	−0.072941	0.048*	
H31B	0.606628	0.345132	−0.004533	0.048*	
H31C	0.511561	0.332131	−0.043403	0.048*	
C32	0.47744 (19)	0.1653 (2)	−0.00743 (15)	0.0268 (7)	
H32A	0.421008	0.195852	−0.015865	0.040*	
H32B	0.467828	0.115863	0.026819	0.040*	
H32C	0.500442	0.143610	−0.055581	0.040*	
I1	0.750000	0.750000	0.62866 (2)	0.01579 (5)	
I2	0.77863 (2)	0.56080 (2)	0.63226 (2)	0.02354 (4)	
I3	0.71724 (2)	0.46647 (2)	0.47548 (2)	0.02059 (4)	
I4	0.68484 (2)	0.39241 (2)	0.33699 (2)	0.03232 (5)	

### Bis(di-tert-butylisopropylphosphine sulfide-κS)gold(I) triiodide bis(iodine) (4) Atomic displacement parameters (Å^2^)

**Table:**

	*U*^11^	*U*^22^	*U*^33^	*U*^12^	*U*^13^	*U*^23^
Au1	0.01159 (6)	0.01993 (7)	0.01425 (7)	−0.00277 (6)	0.000	0.000
P1	0.0117 (3)	0.0120 (3)	0.0126 (3)	−0.0012 (2)	0.0003 (2)	0.0004 (2)
S1	0.0141 (3)	0.0157 (3)	0.0197 (3)	−0.0036 (2)	−0.0016 (2)	−0.0028 (3)
C1	0.0226 (13)	0.0140 (13)	0.0154 (12)	−0.0050 (10)	0.0018 (10)	0.0024 (10)
C2	0.0140 (12)	0.0152 (14)	0.0354 (16)	0.0011 (10)	0.0010 (11)	0.0025 (12)
C3	0.0204 (13)	0.0181 (14)	0.0147 (12)	−0.0035 (10)	0.0009 (10)	−0.0025 (10)
C11	0.0275 (15)	0.0192 (15)	0.0253 (14)	−0.0075 (12)	0.0058 (11)	0.0015 (12)
C12	0.0463 (18)	0.0230 (16)	0.0150 (13)	−0.0090 (14)	−0.0007 (12)	0.0045 (11)
C13	0.0272 (15)	0.0144 (14)	0.0286 (15)	−0.0010 (11)	−0.0019 (12)	0.0048 (11)
C21	0.0229 (15)	0.0171 (15)	0.0477 (19)	0.0013 (12)	−0.0015 (13)	0.0085 (14)
C22	0.0168 (14)	0.0230 (17)	0.068 (2)	0.0005 (12)	−0.0104 (14)	0.0018 (16)
C23	0.0338 (17)	0.0247 (17)	0.0457 (19)	0.0022 (14)	0.0198 (15)	−0.0070 (14)
C31	0.0461 (19)	0.0333 (18)	0.0170 (14)	−0.0106 (15)	0.0044 (13)	0.0017 (13)
C32	0.0290 (15)	0.0313 (17)	0.0201 (14)	−0.0068 (13)	−0.0038 (12)	−0.0050 (12)
I1	0.01799 (11)	0.01698 (12)	0.01240 (10)	−0.00082 (9)	0.000	0.000
I2	0.03629 (10)	0.01414 (9)	0.02018 (9)	0.00234 (8)	−0.00729 (7)	−0.00293 (7)
I3	0.02308 (9)	0.01690 (9)	0.02180 (9)	0.00011 (7)	0.00347 (7)	0.00215 (7)
I4	0.05127 (13)	0.02262 (10)	0.02306 (9)	−0.00318 (9)	0.00405 (9)	−0.00407 (8)

### Bis(di-tert-butylisopropylphosphine sulfide-κS)gold(I) triiodide bis(iodine) (4) Geometric parameters (Å, º)

**Table:**

Au1—S1	2.2818 (6)	C13—H13B	0.9800
Au1—S1^i^	2.2818 (6)	C13—H13C	0.9800
P1—C3	1.850 (3)	C21—H21A	0.9800
P1—C2	1.871 (3)	C21—H21B	0.9800
P1—C1	1.880 (3)	C21—H21C	0.9800
P1—S1	2.0441 (9)	C22—H22A	0.9800
C1—C12	1.531 (4)	C22—H22B	0.9800
C1—C11	1.540 (4)	C22—H22C	0.9800
C1—C13	1.545 (4)	C23—H23A	0.9800
C2—C23	1.536 (4)	C23—H23B	0.9800
C2—C21	1.536 (4)	C23—H23C	0.9800
C2—C22	1.539 (4)	C31—H31A	0.9800
C3—C31	1.535 (4)	C31—H31B	0.9800
C3—C32	1.540 (4)	C31—H31C	0.9800
C3—H3	1.0000	C32—H32A	0.9800
C11—H11A	0.9800	C32—H32B	0.9800
C11—H11B	0.9800	C32—H32C	0.9800
C11—H11C	0.9800	I1—I2^ii^	2.9204 (2)
C12—H12A	0.9800	I1—I2	2.9204 (2)
C12—H12B	0.9800	I2—I3	3.2674 (2)
C12—H12C	0.9800	I3—I4	2.7501 (3)
C13—H13A	0.9800		
			
S1—Au1—S1^i^	176.40 (3)	C1—C13—H13B	109.5
C3—P1—C2	112.96 (13)	H13A—C13—H13B	109.5
C3—P1—C1	108.19 (12)	C1—C13—H13C	109.5
C2—P1—C1	113.66 (12)	H13A—C13—H13C	109.5
C3—P1—S1	107.64 (9)	H13B—C13—H13C	109.5
C2—P1—S1	102.89 (9)	C2—C21—H21A	109.5
C1—P1—S1	111.31 (9)	C2—C21—H21B	109.5
P1—S1—Au1	107.14 (3)	H21A—C21—H21B	109.5
C12—C1—C11	108.9 (2)	C2—C21—H21C	109.5
C12—C1—C13	107.3 (2)	H21A—C21—H21C	109.5
C11—C1—C13	108.6 (2)	H21B—C21—H21C	109.5
C12—C1—P1	110.76 (18)	C2—C22—H22A	109.5
C11—C1—P1	112.23 (18)	C2—C22—H22B	109.5
C13—C1—P1	108.94 (17)	H22A—C22—H22B	109.5
C23—C2—C21	106.9 (2)	C2—C22—H22C	109.5
C23—C2—C22	110.4 (2)	H22A—C22—H22C	109.5
C21—C2—C22	108.6 (2)	H22B—C22—H22C	109.5
C23—C2—P1	110.3 (2)	C2—C23—H23A	109.5
C21—C2—P1	110.34 (18)	C2—C23—H23B	109.5
C22—C2—P1	110.2 (2)	H23A—C23—H23B	109.5
C31—C3—C32	109.9 (2)	C2—C23—H23C	109.5
C31—C3—P1	113.03 (19)	H23A—C23—H23C	109.5
C32—C3—P1	117.56 (18)	H23B—C23—H23C	109.5
C31—C3—H3	105.1	C3—C31—H31A	109.5
C32—C3—H3	105.1	C3—C31—H31B	109.5
P1—C3—H3	105.1	H31A—C31—H31B	109.5
C1—C11—H11A	109.5	C3—C31—H31C	109.5
C1—C11—H11B	109.5	H31A—C31—H31C	109.5
H11A—C11—H11B	109.5	H31B—C31—H31C	109.5
C1—C11—H11C	109.5	C3—C32—H32A	109.5
H11A—C11—H11C	109.5	C3—C32—H32B	109.5
H11B—C11—H11C	109.5	H32A—C32—H32B	109.5
C1—C12—H12A	109.5	C3—C32—H32C	109.5
C1—C12—H12B	109.5	H32A—C32—H32C	109.5
H12A—C12—H12B	109.5	H32B—C32—H32C	109.5
C1—C12—H12C	109.5	I2^ii^—I1—I2	177.495 (11)
H12A—C12—H12C	109.5	I1—I2—I3	112.048 (7)
H12B—C12—H12C	109.5	I4—I3—I2	173.271 (9)
C1—C13—H13A	109.5		
			
C3—P1—S1—Au1	−58.76 (10)	S1—P1—C2—C23	−74.7 (2)
C2—P1—S1—Au1	−178.28 (9)	C3—P1—C2—C21	−72.5 (2)
C1—P1—S1—Au1	59.64 (9)	C1—P1—C2—C21	163.74 (19)
C3—P1—C1—C12	155.4 (2)	S1—P1—C2—C21	43.3 (2)
C2—P1—C1—C12	−78.3 (2)	C3—P1—C2—C22	47.4 (2)
S1—P1—C1—C12	37.3 (2)	C1—P1—C2—C22	−76.4 (2)
C3—P1—C1—C11	−82.7 (2)	S1—P1—C2—C22	163.14 (19)
C2—P1—C1—C11	43.6 (2)	C2—P1—C3—C31	56.4 (2)
S1—P1—C1—C11	159.21 (16)	C1—P1—C3—C31	−176.9 (2)
C3—P1—C1—C13	37.5 (2)	S1—P1—C3—C31	−56.5 (2)
C2—P1—C1—C13	163.85 (18)	C2—P1—C3—C32	−73.3 (2)
S1—P1—C1—C13	−80.53 (18)	C1—P1—C3—C32	53.4 (2)
C3—P1—C2—C23	169.55 (19)	S1—P1—C3—C32	173.81 (19)
C1—P1—C2—C23	45.8 (2)		

Symmetry codes: (i) −*x*+3/2, −*y*+1/2, *z*; (ii) −*x*+3/2, −*y*+3/2, *z*.

### Bis(di-tert-butylisopropylphosphine sulfide-κS)gold(I) triiodide bis(iodine) (4) Hydrogen-bond geometry (Å, º)

**Table:**

*D*—H···*A*	*D*—H	H···*A*	*D*···*A*	*D*—H···*A*
C13—H13*A*···Au1	0.98	2.70	3.594 (3)	152
C21—H21*C*···S1	0.98	2.64	3.170 (3)	114
C31—H31*B*···I3^iii^	0.98	3.25	4.102 (3)	146
C3—H3···I3^iv^	1.00	3.18	4.059 (3)	148
C21—H21*A*···I3^v^	0.98	3.28	4.119 (3)	145
C22—H22*C*···I3^v^	0.98	3.18	3.962 (3)	138

Symmetry codes: (iii) −*x*+3/2, *y*, *z*−1/2; (iv) *x*, −*y*+1/2, *z*−1/2; (v) *x*−1/2, −*y*+1, −*z*+1/2.

### Dibromido(di-tert-butyldithiophosphato-κ2S,S')gold(III) (5) Crystal data

**Table:**

[AuBr_2_(C_8_H_18_PS_2_)]	*D*_x_ = 2.525 Mg m^−^^3^
*M**_r_* = 566.10	Mo *K*α radiation, λ = 0.71073 Å
Orthorhombic, *P**n**m**a*	Cell parameters from 5608 reflections
*a* = 16.0199 (6) Å	θ = 2.2–29.2°
*b* = 11.5880 (4) Å	µ = 15.60 mm^−^^1^
*c* = 8.0211 (3) Å	*T* = 100 K
*V* = 1489.03 (9) Å^3^	Block, orange
*Z* = 4	0.13 × 0.05 × 0.04 mm
*F*(000) = 1048	

### Dibromido(di-tert-butyldithiophosphato-κ2S,S')gold(III) (5) Data collection

**Table:**

Oxford Diffraction Xcalibur, Eos diffractometer	2061 independent reflections
Radiation source: fine-focus sealed tube	1712 reflections with *I* > 2σ(*I*)
Graphite monochromator	*R*_int_ = 0.076
Detector resolution: 16.1419 pixels mm^-1^	θ_max_ = 29.3°, θ_min_ = 2.5°
ω scan	*h* = −21→21
Absorption correction: multi-scan (CrysAlisPro; Rigaku OD, 2015)	*k* = −15→15
*T*_min_ = 0.635, *T*_max_ = 1.000	*l* = −10→11
35299 measured reflections	

### Dibromido(di-tert-butyldithiophosphato-κ2S,S')gold(III) (5) Refinement

**Table:**

Refinement on *F*^2^	Primary atom site location: structure-invariant direct methods
Least-squares matrix: full	Secondary atom site location: difference Fourier map
*R*[*F*^2^ > 2σ(*F*^2^)] = 0.029	Hydrogen site location: mixed
*wR*(*F*^2^) = 0.056	H-atom parameters constrained
*S* = 1.06	*w* = 1/[σ^2^(*F*_o_^2^) + (0.0213*P*)^2^ + 4.1938*P*] where *P* = (*F*_o_^2^ + 2*F*_c_^2^)/3
2061 reflections	(Δ/σ)_max_ = 0.001
75 parameters	Δρ_max_ = 1.88 e Å^−^^3^
0 restraints	Δρ_min_ = −1.03 e Å^−^^3^

### Dibromido(di-tert-butyldithiophosphato-κ2S,S')gold(III) (5) Fractional atomic coordinates and isotropic or equivalent isotropic displacement parameters (Å^2^)

**Table:**

	*x*	*y*	*z*	*U*_iso_*/*U*_eq_	
Au1	0.69861 (2)	0.250000	0.53055 (3)	0.01406 (8)	
Br1	0.78044 (3)	0.09686 (4)	0.65922 (6)	0.02188 (12)	
P1	0.55355 (10)	0.250000	0.2844 (2)	0.0134 (3)	
S1	0.61299 (7)	0.11433 (9)	0.40022 (15)	0.0177 (2)	
C1	0.4409 (4)	0.250000	0.3401 (8)	0.0183 (14)	
C2	0.5817 (4)	0.250000	0.0578 (7)	0.0140 (13)	
C11	0.4353 (5)	0.250000	0.5312 (9)	0.0327 (18)	
H11A	0.377742	0.250000	0.564559	0.049*	
H11B	0.462352	0.182380	0.574289	0.049*	
C12	0.3999 (3)	0.1394 (4)	0.2736 (7)	0.0306 (12)	
H12A	0.345171	0.129215	0.326056	0.046*	
H12B	0.435443	0.072933	0.299805	0.046*	
H12C	0.393055	0.145348	0.152515	0.046*	
C21	0.6774 (4)	0.250000	0.0489 (9)	0.0239 (16)	
H21A	0.694889	0.250000	−0.065730	0.036*	
H21B	0.698819	0.317640	0.103340	0.036*	
C22	0.5488 (3)	0.1413 (4)	−0.0271 (6)	0.0250 (11)	
H22A	0.574317	0.133728	−0.137638	0.037*	
H22B	0.488033	0.146697	−0.038855	0.037*	
H22C	0.562906	0.073642	0.040551	0.037*	

### Dibromido(di-tert-butyldithiophosphato-κ2S,S')gold(III) (5) Atomic displacement parameters (Å^2^)

**Table:**

	*U*^11^	*U*^22^	*U*^33^	*U*^12^	*U*^13^	*U*^23^
Au1	0.01107 (12)	0.01622 (12)	0.01489 (13)	0.000	−0.00084 (10)	0.000
Br1	0.0198 (2)	0.0214 (2)	0.0244 (3)	0.00509 (18)	−0.00556 (19)	0.00058 (19)
P1	0.0107 (8)	0.0134 (8)	0.0161 (8)	0.000	−0.0006 (7)	0.000
S1	0.0180 (6)	0.0139 (5)	0.0212 (6)	0.0003 (4)	−0.0047 (5)	0.0007 (5)
C1	0.007 (3)	0.023 (3)	0.025 (4)	0.000	0.004 (3)	0.000
C2	0.015 (3)	0.014 (3)	0.013 (3)	0.000	0.000 (2)	0.000
C11	0.029 (4)	0.046 (5)	0.023 (4)	0.000	0.014 (3)	0.000
C12	0.014 (2)	0.033 (3)	0.045 (3)	−0.008 (2)	0.009 (2)	−0.007 (3)
C21	0.016 (3)	0.029 (4)	0.027 (4)	0.000	0.009 (3)	0.000
C22	0.031 (3)	0.025 (3)	0.020 (2)	−0.008 (2)	0.000 (2)	−0.006 (2)

### Dibromido(di-tert-butyldithiophosphato-κ2S,S')gold(III) (5) Geometric parameters (Å, º)

**Table:**

Au1—S1	2.3336 (11)	C2—C21	1.535 (9)
Au1—S1^i^	2.3336 (11)	C11—H11A	0.9599
Au1—Br1^i^	2.4357 (5)	C11—H11B	0.9598
Au1—Br1	2.4357 (5)	C11—H11B^i^	0.9598
P1—C1	1.859 (6)	C12—H12A	0.9800
P1—C2	1.873 (6)	C12—H12B	0.9800
P1—S1^i^	2.0594 (15)	C12—H12C	0.9800
P1—S1	2.0594 (15)	C21—H21A	0.9609
C1—C11	1.535 (10)	C21—H21B	0.9605
C1—C12^i^	1.536 (6)	C21—H21B^i^	0.9605
C1—C12	1.536 (6)	C22—H22A	0.9800
C2—C22	1.526 (6)	C22—H22B	0.9800
C2—C22^i^	1.526 (6)	C22—H22C	0.9800
			
S1—Au1—S1^i^	84.71 (5)	C1—C11—H11A	109.5
S1—Au1—Br1^i^	175.54 (3)	C1—C11—H11B	109.5
S1^i^—Au1—Br1^i^	90.88 (3)	H11A—C11—H11B	109.5
S1—Au1—Br1	90.88 (3)	C1—C11—H11B^i^	109.45 (19)
S1^i^—Au1—Br1	175.54 (3)	H11A—C11—H11B^i^	109.5
Br1^i^—Au1—Br1	93.53 (2)	H11B—C11—H11B^i^	109.4
C1—P1—C2	117.8 (3)	C1—C12—H12A	109.5
C1—P1—S1^i^	109.91 (14)	C1—C12—H12B	109.5
C2—P1—S1^i^	109.04 (13)	H12A—C12—H12B	109.5
C1—P1—S1	109.91 (14)	C1—C12—H12C	109.5
C2—P1—S1	109.04 (13)	H12A—C12—H12C	109.5
S1^i^—P1—S1	99.53 (9)	H12B—C12—H12C	109.5
P1—S1—Au1	87.68 (5)	C2—C21—H21A	109.6
C11—C1—C12^i^	108.8 (4)	C2—C21—H21B	109.6
C11—C1—C12	108.8 (4)	H21A—C21—H21B	109.3
C12^i^—C1—C12	113.2 (6)	C2—C21—H21B^i^	109.58 (18)
C11—C1—P1	107.2 (5)	H21A—C21—H21B^i^	109.3
C12^i^—C1—P1	109.3 (3)	H21B—C21—H21B^i^	109.4
C12—C1—P1	109.3 (3)	C2—C22—H22A	109.5
C22—C2—C22^i^	111.3 (5)	C2—C22—H22B	109.5
C22—C2—C21	108.9 (4)	H22A—C22—H22B	109.5
C22^i^—C2—C21	108.9 (4)	C2—C22—H22C	109.5
C22—C2—P1	110.5 (3)	H22A—C22—H22C	109.5
C22^i^—C2—P1	110.5 (3)	H22B—C22—H22C	109.5
C21—C2—P1	106.6 (4)		
			
C1—P1—S1—Au1	−120.49 (19)	S1^i^—P1—C1—C12	−172.1 (3)
C2—P1—S1—Au1	108.93 (17)	S1—P1—C1—C12	−63.5 (4)
S1^i^—P1—S1—Au1	−5.15 (8)	C1—P1—C2—C22	−61.8 (4)
S1^i^—Au1—S1—P1	4.50 (7)	S1^i^—P1—C2—C22	172.1 (3)
Br1—Au1—S1—P1	−176.12 (6)	S1—P1—C2—C22	64.4 (4)
C2—P1—C1—C11	180.0	C1—P1—C2—C22^i^	61.8 (4)
S1^i^—P1—C1—C11	−54.28 (9)	S1^i^—P1—C2—C22^i^	−64.4 (4)
S1—P1—C1—C11	54.28 (9)	S1—P1—C2—C22^i^	−172.1 (3)
C2—P1—C1—C12^i^	−62.2 (4)	C1—P1—C2—C21	180.0
S1^i^—P1—C1—C12^i^	63.5 (4)	S1^i^—P1—C2—C21	53.86 (8)
S1—P1—C1—C12^i^	172.1 (3)	S1—P1—C2—C21	−53.86 (8)
C2—P1—C1—C12	62.2 (4)		

Symmetry code: (i) *x*, −*y*+1/2, *z*.

### Dibromido(di-tert-butyldithiophosphato-κ2S,S')gold(III) (5) Hydrogen-bond geometry (Å, º)

**Table:**

*D*—H···*A*	*D*—H	H···*A*	*D*···*A*	*D*—H···*A*
C11—H11*B*···S1	0.96	2.90	3.417 (7)	115
C12—H12*B*···S1	0.98	2.99	3.573 (5)	119
C12—H12*C*···Br1^ii^	0.98	3.13	3.995 (6)	147

Symmetry code: (ii) *x*−1/2, *y*, −*z*+1/2.

### Di-tert-butyl{[di-tert-butyl(hydroxy)phosphanyl]diselanyl}phosphine oxide tetrabromidoaurate(III) (6) Crystal data

**Table:**

(C_16_H_37_O_2_P_2_Se_2_)[AuBr_4_]	*F*(000) = 1864
*M**_r_* = 997.92	*D*_x_ = 2.323 Mg m^−^^3^
Monoclinic, *P*2_1_/*c*	Mo *K*α radiation, λ = 0.71073 Å
*a* = 8.4102 (2) Å	Cell parameters from 11700 reflections
*b* = 22.2796 (7) Å	θ = 2.3–30.8°
*c* = 15.4285 (5) Å	µ = 13.43 mm^−^^1^
β = 99.221 (3)°	*T* = 100 K
*V* = 2853.57 (16) Å^3^	Needle, red
*Z* = 4	0.2 × 0.02 × 0.02 mm

### Di-tert-butyl{[di-tert-butyl(hydroxy)phosphanyl]diselanyl}phosphine oxide tetrabromidoaurate(III) (6) Data collection

**Table:**

Oxford Diffraction Xcalibur, Eos diffractometer	7081 independent reflections
Radiation source: fine-focus sealed tube	5668 reflections with *I* > 2σ(*I*)
Graphite monochromator	*R*_int_ = 0.116
Detector resolution: 16.1419 pixels mm^-1^	θ_max_ = 28.3°, θ_min_ = 2.3°
ω scan	*h* = −11→11
Absorption correction: multi-scan (CrysAlisPro; Rigaku OD, 2015)	*k* = −29→29
*T*_min_ = 0.514, *T*_max_ = 1.000	*l* = −20→20
133232 measured reflections	

### Di-tert-butyl{[di-tert-butyl(hydroxy)phosphanyl]diselanyl}phosphine oxide tetrabromidoaurate(III) (6) Refinement

**Table:**

Refinement on *F*^2^	Primary atom site location: structure-invariant direct methods
Least-squares matrix: full	Secondary atom site location: difference Fourier map
*R*[*F*^2^ > 2σ(*F*^2^)] = 0.036	Hydrogen site location: mixed
*wR*(*F*^2^) = 0.056	H atoms treated by a mixture of independent and constrained refinement
*S* = 1.05	*w* = 1/[σ^2^(*F*_o_^2^) + (0.0158*P*)^2^ + 4.8488*P*] where *P* = (*F*_o_^2^ + 2*F*_c_^2^)/3
7081 reflections	(Δ/σ)_max_ = 0.001
260 parameters	Δρ_max_ = 0.98 e Å^−^^3^
0 restraints	Δρ_min_ = −1.23 e Å^−^^3^

### Di-tert-butyl{[di-tert-butyl(hydroxy)phosphanyl]diselanyl}phosphine oxide tetrabromidoaurate(III) (6) Fractional atomic coordinates and isotropic or equivalent isotropic displacement parameters (Å^2^)

**Table:**

	*x*	*y*	*z*	*U*_iso_*/*U*_eq_	
Au1	0.05652 (2)	0.81535 (2)	0.63141 (2)	0.01639 (5)	
Br1	0.32579 (6)	0.77573 (2)	0.63472 (4)	0.02534 (12)	
Br2	0.05468 (7)	0.84795 (2)	0.48100 (4)	0.02980 (13)	
Br3	−0.20963 (6)	0.85787 (3)	0.63120 (4)	0.03423 (14)	
Br4	0.05827 (6)	0.77788 (3)	0.77868 (3)	0.03090 (13)	
O1	0.5129 (4)	0.54434 (14)	0.6781 (2)	0.0171 (7)	
O2	0.6712 (4)	0.55674 (14)	0.5591 (2)	0.0170 (7)	
H01	0.570 (9)	0.544 (3)	0.630 (5)	0.10 (3)*	
P1	0.59386 (14)	0.55943 (5)	0.77147 (8)	0.0137 (2)	
P2	0.71014 (13)	0.62122 (5)	0.53602 (8)	0.0131 (2)	
Se1	0.79131 (5)	0.62905 (2)	0.77042 (3)	0.01654 (10)	
Se2	0.68353 (5)	0.68495 (2)	0.64720 (3)	0.01594 (10)	
C1	0.4362 (5)	0.5899 (2)	0.8296 (3)	0.0179 (10)	
C2	0.7096 (5)	0.4945 (2)	0.8223 (3)	0.0171 (10)	
C3	0.5561 (5)	0.6514 (2)	0.4476 (3)	0.0181 (10)	
C4	0.9198 (5)	0.6217 (2)	0.5140 (3)	0.0184 (10)	
C11	0.5020 (6)	0.6009 (2)	0.9269 (3)	0.0288 (12)	
H11A	0.602879	0.623591	0.931992	0.043*	
H11B	0.522035	0.562303	0.957086	0.043*	
H11C	0.423043	0.623833	0.953643	0.043*	
C12	0.2893 (5)	0.5468 (2)	0.8181 (4)	0.0273 (12)	
H12A	0.318166	0.509949	0.851539	0.041*	
H12B	0.258168	0.536986	0.755785	0.041*	
H12C	0.198899	0.566373	0.839721	0.041*	
C13	0.3808 (6)	0.6501 (2)	0.7856 (3)	0.0226 (11)	
H13A	0.291760	0.666283	0.812351	0.034*	
H13B	0.344618	0.643650	0.722724	0.034*	
H13C	0.470805	0.678527	0.793851	0.034*	
C21	0.5922 (6)	0.4479 (2)	0.8498 (3)	0.0230 (11)	
H21A	0.508311	0.438923	0.799766	0.034*	
H21B	0.542358	0.464126	0.898128	0.034*	
H21C	0.651095	0.411119	0.869199	0.034*	
C22	0.8337 (6)	0.5129 (2)	0.9026 (3)	0.0266 (12)	
H22A	0.777984	0.531999	0.946445	0.040*	
H22B	0.911066	0.541136	0.884195	0.040*	
H22C	0.890810	0.477112	0.928169	0.040*	
C23	0.7979 (6)	0.4679 (2)	0.7520 (3)	0.0240 (11)	
H23A	0.863855	0.433873	0.776674	0.036*	
H23B	0.867312	0.498614	0.732084	0.036*	
H23C	0.719100	0.454214	0.702145	0.036*	
C31	0.3933 (5)	0.6296 (2)	0.4700 (3)	0.0270 (12)	
H31A	0.305298	0.646371	0.427545	0.041*	
H31B	0.382288	0.642982	0.529238	0.041*	
H31C	0.389010	0.585703	0.467339	0.041*	
C32	0.5779 (6)	0.6264 (2)	0.3577 (3)	0.0269 (12)	
H32A	0.588314	0.582671	0.361247	0.040*	
H32B	0.675177	0.643655	0.340212	0.040*	
H32C	0.484012	0.637045	0.314134	0.040*	
C33	0.5555 (6)	0.7204 (2)	0.4448 (3)	0.0247 (12)	
H33A	0.658113	0.734763	0.430186	0.037*	
H33B	0.540983	0.736200	0.502313	0.037*	
H33C	0.466950	0.734275	0.400114	0.037*	
C41	1.0289 (5)	0.6047 (2)	0.5996 (3)	0.0245 (12)	
H41A	0.993672	0.566263	0.620921	0.037*	
H41B	1.022544	0.635872	0.643777	0.037*	
H41C	1.140343	0.601134	0.589066	0.037*	
C42	0.9628 (6)	0.6851 (2)	0.4869 (3)	0.0253 (11)	
H42A	0.932398	0.714290	0.528964	0.038*	
H42B	0.904422	0.694038	0.428147	0.038*	
H42C	1.078965	0.687650	0.486451	0.038*	
C43	0.9384 (6)	0.5757 (2)	0.4427 (4)	0.0269 (12)	
H43A	1.053055	0.569000	0.441198	0.040*	
H43B	0.886097	0.590918	0.385539	0.040*	
H43C	0.887828	0.537819	0.455537	0.040*	

### Di-tert-butyl{[di-tert-butyl(hydroxy)phosphanyl]diselanyl}phosphine oxide tetrabromidoaurate(III) (6) Atomic displacement parameters (Å^2^)

**Table:**

	*U*^11^	*U*^22^	*U*^33^	*U*^12^	*U*^13^	*U*^23^
Au1	0.01627 (9)	0.01422 (9)	0.01811 (10)	0.00158 (7)	0.00101 (7)	−0.00194 (8)
Br1	0.0205 (2)	0.0253 (3)	0.0315 (3)	0.0076 (2)	0.0079 (2)	0.0037 (2)
Br2	0.0392 (3)	0.0253 (3)	0.0236 (3)	0.0003 (2)	0.0008 (2)	0.0092 (2)
Br3	0.0199 (3)	0.0354 (3)	0.0466 (4)	0.0098 (2)	0.0030 (2)	−0.0010 (3)
Br4	0.0302 (3)	0.0465 (3)	0.0167 (3)	0.0043 (2)	0.0061 (2)	0.0009 (2)
O1	0.0168 (17)	0.0209 (17)	0.0135 (18)	−0.0023 (14)	0.0026 (14)	0.0038 (14)
O2	0.0164 (16)	0.0176 (17)	0.0177 (18)	−0.0028 (13)	0.0050 (14)	0.0008 (14)
P1	0.0137 (6)	0.0148 (6)	0.0126 (6)	−0.0015 (5)	0.0019 (5)	0.0019 (5)
P2	0.0127 (6)	0.0140 (6)	0.0130 (6)	−0.0010 (5)	0.0034 (5)	0.0000 (5)
Se1	0.0163 (2)	0.0180 (2)	0.0145 (2)	−0.00416 (18)	−0.00021 (18)	0.00199 (19)
Se2	0.0209 (2)	0.0134 (2)	0.0140 (2)	0.00058 (19)	0.00425 (18)	0.0006 (2)
C1	0.022 (2)	0.018 (2)	0.016 (3)	0.0031 (19)	0.010 (2)	0.004 (2)
C2	0.016 (2)	0.017 (2)	0.017 (3)	0.0004 (19)	−0.0003 (19)	0.005 (2)
C3	0.017 (2)	0.023 (3)	0.014 (2)	−0.001 (2)	0.0011 (19)	0.000 (2)
C4	0.012 (2)	0.024 (3)	0.021 (3)	−0.0001 (19)	0.0074 (19)	−0.002 (2)
C11	0.036 (3)	0.038 (3)	0.015 (3)	0.005 (3)	0.010 (2)	−0.002 (2)
C12	0.018 (3)	0.028 (3)	0.039 (3)	−0.002 (2)	0.012 (2)	0.006 (3)
C13	0.022 (3)	0.019 (3)	0.028 (3)	0.004 (2)	0.009 (2)	0.000 (2)
C21	0.026 (3)	0.017 (2)	0.026 (3)	−0.003 (2)	0.005 (2)	0.003 (2)
C22	0.029 (3)	0.025 (3)	0.023 (3)	0.002 (2)	−0.003 (2)	0.011 (2)
C23	0.022 (3)	0.019 (3)	0.030 (3)	0.004 (2)	0.004 (2)	0.002 (2)
C31	0.012 (2)	0.043 (3)	0.024 (3)	0.001 (2)	−0.001 (2)	0.004 (3)
C32	0.027 (3)	0.036 (3)	0.016 (3)	0.000 (2)	0.000 (2)	0.000 (2)
C33	0.029 (3)	0.027 (3)	0.019 (3)	0.006 (2)	0.005 (2)	0.003 (2)
C41	0.013 (2)	0.037 (3)	0.024 (3)	0.003 (2)	0.003 (2)	0.001 (2)
C42	0.022 (2)	0.028 (3)	0.030 (3)	−0.006 (2)	0.016 (2)	−0.002 (2)
C43	0.019 (3)	0.032 (3)	0.032 (3)	0.005 (2)	0.009 (2)	−0.004 (2)

### Di-tert-butyl{[di-tert-butyl(hydroxy)phosphanyl]diselanyl}phosphine oxide tetrabromidoaurate(III) (6) Geometric parameters (Å, º)

**Table:**

Au1—Br4	2.4185 (6)	C12—H12C	0.9800
Au1—Br1	2.4235 (5)	C13—H13A	0.9800
Au1—Br2	2.4291 (6)	C13—H13B	0.9800
Au1—Br3	2.4301 (5)	C13—H13C	0.9800
O1—P1	1.528 (3)	C21—H21A	0.9800
O1—H01	0.95 (8)	C21—H21B	0.9800
O2—P2	1.528 (3)	C21—H21C	0.9800
P1—C1	1.845 (5)	C22—H22A	0.9800
P1—C2	1.846 (5)	C22—H22B	0.9800
P1—Se1	2.2743 (12)	C22—H22C	0.9800
P2—C4	1.848 (4)	C23—H23A	0.9800
P2—C3	1.851 (5)	C23—H23B	0.9800
P2—Se2	2.2653 (13)	C23—H23C	0.9800
Se1—Se2	2.3314 (6)	C31—H31A	0.9800
C1—C11	1.534 (7)	C31—H31B	0.9800
C1—C13	1.540 (6)	C31—H31C	0.9800
C1—C12	1.552 (6)	C32—H32A	0.9800
C2—C23	1.529 (7)	C32—H32B	0.9800
C2—C21	1.538 (6)	C32—H32C	0.9800
C2—C22	1.541 (6)	C33—H33A	0.9800
C3—C32	1.533 (6)	C33—H33B	0.9800
C3—C33	1.538 (6)	C33—H33C	0.9800
C3—C31	1.543 (6)	C41—H41A	0.9800
C4—C43	1.529 (7)	C41—H41B	0.9800
C4—C41	1.530 (7)	C41—H41C	0.9800
C4—C42	1.534 (6)	C42—H42A	0.9800
C11—H11A	0.9800	C42—H42B	0.9800
C11—H11B	0.9800	C42—H42C	0.9800
C11—H11C	0.9800	C43—H43A	0.9800
C12—H12A	0.9800	C43—H43B	0.9800
C12—H12B	0.9800	C43—H43C	0.9800
			
Br4—Au1—Br1	89.397 (19)	H13A—C13—H13B	109.5
Br4—Au1—Br2	177.20 (2)	C1—C13—H13C	109.5
Br1—Au1—Br2	89.539 (19)	H13A—C13—H13C	109.5
Br4—Au1—Br3	90.15 (2)	H13B—C13—H13C	109.5
Br1—Au1—Br3	178.07 (2)	C2—C21—H21A	109.5
Br2—Au1—Br3	90.99 (2)	C2—C21—H21B	109.5
P1—O1—H01	122 (5)	H21A—C21—H21B	109.5
O1—P1—C1	107.0 (2)	C2—C21—H21C	109.5
O1—P1—C2	110.7 (2)	H21A—C21—H21C	109.5
C1—P1—C2	116.9 (2)	H21B—C21—H21C	109.5
O1—P1—Se1	111.02 (13)	C2—C22—H22A	109.5
C1—P1—Se1	109.55 (16)	C2—C22—H22B	109.5
C2—P1—Se1	101.62 (15)	H22A—C22—H22B	109.5
O2—P2—C4	107.1 (2)	C2—C22—H22C	109.5
O2—P2—C3	111.2 (2)	H22A—C22—H22C	109.5
C4—P2—C3	116.0 (2)	H22B—C22—H22C	109.5
O2—P2—Se2	111.23 (13)	C2—C23—H23A	109.5
C4—P2—Se2	110.42 (16)	C2—C23—H23B	109.5
C3—P2—Se2	100.82 (16)	H23A—C23—H23B	109.5
P1—Se1—Se2	100.44 (4)	C2—C23—H23C	109.5
P2—Se2—Se1	102.12 (4)	H23A—C23—H23C	109.5
C11—C1—C13	109.2 (4)	H23B—C23—H23C	109.5
C11—C1—C12	111.5 (4)	C3—C31—H31A	109.5
C13—C1—C12	108.0 (4)	C3—C31—H31B	109.5
C11—C1—P1	111.1 (3)	H31A—C31—H31B	109.5
C13—C1—P1	107.3 (3)	C3—C31—H31C	109.5
C12—C1—P1	109.7 (3)	H31A—C31—H31C	109.5
C23—C2—C21	109.9 (4)	H31B—C31—H31C	109.5
C23—C2—C22	109.2 (4)	C3—C32—H32A	109.5
C21—C2—C22	109.8 (4)	C3—C32—H32B	109.5
C23—C2—P1	106.6 (3)	H32A—C32—H32B	109.5
C21—C2—P1	109.1 (3)	C3—C32—H32C	109.5
C22—C2—P1	112.1 (3)	H32A—C32—H32C	109.5
C32—C3—C33	109.7 (4)	H32B—C32—H32C	109.5
C32—C3—C31	109.1 (4)	C3—C33—H33A	109.5
C33—C3—C31	108.8 (4)	C3—C33—H33B	109.5
C32—C3—P2	111.3 (3)	H33A—C33—H33B	109.5
C33—C3—P2	112.5 (3)	C3—C33—H33C	109.5
C31—C3—P2	105.2 (3)	H33A—C33—H33C	109.5
C43—C4—C41	109.9 (4)	H33B—C33—H33C	109.5
C43—C4—C42	111.5 (4)	C4—C41—H41A	109.5
C41—C4—C42	109.1 (4)	C4—C41—H41B	109.5
C43—C4—P2	109.8 (3)	H41A—C41—H41B	109.5
C41—C4—P2	107.3 (3)	C4—C41—H41C	109.5
C42—C4—P2	109.1 (3)	H41A—C41—H41C	109.5
C1—C11—H11A	109.5	H41B—C41—H41C	109.5
C1—C11—H11B	109.5	C4—C42—H42A	109.5
H11A—C11—H11B	109.5	C4—C42—H42B	109.5
C1—C11—H11C	109.5	H42A—C42—H42B	109.5
H11A—C11—H11C	109.5	C4—C42—H42C	109.5
H11B—C11—H11C	109.5	H42A—C42—H42C	109.5
C1—C12—H12A	109.5	H42B—C42—H42C	109.5
C1—C12—H12B	109.5	C4—C43—H43A	109.5
H12A—C12—H12B	109.5	C4—C43—H43B	109.5
C1—C12—H12C	109.5	H43A—C43—H43B	109.5
H12A—C12—H12C	109.5	C4—C43—H43C	109.5
H12B—C12—H12C	109.5	H43A—C43—H43C	109.5
C1—C13—H13A	109.5	H43B—C43—H43C	109.5
C1—C13—H13B	109.5		
			
O1—P1—Se1—Se2	32.74 (14)	O1—P1—C2—C22	162.0 (3)
C1—P1—Se1—Se2	−85.24 (17)	C1—P1—C2—C22	−75.1 (4)
C2—P1—Se1—Se2	150.45 (16)	Se1—P1—C2—C22	44.1 (3)
O2—P2—Se2—Se1	39.99 (14)	O2—P2—C3—C32	−76.6 (4)
C4—P2—Se2—Se1	−78.79 (17)	C4—P2—C3—C32	46.1 (4)
C3—P2—Se2—Se1	158.03 (15)	Se2—P2—C3—C32	165.4 (3)
P1—Se1—Se2—P2	−71.86 (5)	O2—P2—C3—C33	159.7 (3)
O1—P1—C1—C11	174.5 (3)	C4—P2—C3—C33	−77.5 (4)
C2—P1—C1—C11	49.8 (4)	Se2—P2—C3—C33	41.7 (3)
Se1—P1—C1—C11	−65.0 (4)	O2—P2—C3—C31	41.5 (4)
O1—P1—C1—C13	−66.2 (4)	C4—P2—C3—C31	164.2 (3)
C2—P1—C1—C13	169.1 (3)	Se2—P2—C3—C31	−76.6 (3)
Se1—P1—C1—C13	54.2 (3)	O2—P2—C4—C43	55.4 (4)
O1—P1—C1—C12	50.8 (4)	C3—P2—C4—C43	−69.5 (4)
C2—P1—C1—C12	−73.9 (4)	Se2—P2—C4—C43	176.6 (3)
Se1—P1—C1—C12	171.3 (3)	O2—P2—C4—C41	−64.1 (4)
O1—P1—C2—C23	42.5 (4)	C3—P2—C4—C41	171.1 (3)
C1—P1—C2—C23	165.4 (3)	Se2—P2—C4—C41	57.2 (3)
Se1—P1—C2—C23	−75.4 (3)	O2—P2—C4—C42	177.8 (3)
O1—P1—C2—C21	−76.1 (4)	C3—P2—C4—C42	52.9 (4)
C1—P1—C2—C21	46.7 (4)	Se2—P2—C4—C42	−60.9 (4)
Se1—P1—C2—C21	165.9 (3)		

### Di-tert-butyl{[di-tert-butyl(hydroxy)phosphanyl]diselanyl}phosphine oxide tetrabromidoaurate(III) (6) Hydrogen-bond geometry (Å, º)

**Table:**

*D*—H···*A*	*D*—H	H···*A*	*D*···*A*	*D*—H···*A*
C42—H42*A*···Au1^i^	0.98	2.85	3.666 (5)	141
C21—H21*C*···Br1^ii^	0.98	3.02	3.899 (5)	149
C22—H22*B*···Br2^iii^	0.98	3.04	3.717 (5)	128
C33—H33*B*···Br1	0.98	3.07	3.956 (5)	151
C42—H42*B*···Br4^iv^	0.98	2.89	3.532 (5)	124
O1—H01···O2	0.95 (8)	1.51 (8)	2.450 (4)	169 (8)

Symmetry codes: (i) *x*+1, *y*, *z*; (ii) −*x*+1, *y*−1/2, −*z*+3/2; (iii) *x*+1, −*y*+3/2, *z*+1/2; (iv) *x*+1, −*y*+3/2, *z*−1/2.
